# Prevalence of posttraumatic stress disorder in paediatric patients following orthopaedic trauma: A systematic review

**DOI:** 10.1177/18632521261419773

**Published:** 2026-02-18

**Authors:** Janne L Punski-Hoogervorst, Lotje A Hoogervorst, Jan W Schoones, Avi Avital, Pieter Bas de Witte

**Affiliations:** 1 Department of Occupational Therapy, Faculty of Social Welfare and Health Sciences, University of Haifa, Haifa, Israel; 2 Department of Orthopaedics, Leiden University Medical Center, Leiden, the Netherlands; 3 Research Policy Directorate, Leiden University Medical Center, Leiden, the Netherlands; 4 Erasmus MC Sophia Children Hospital, Pediatric Orthopaedics, Rotterdam, the Netherlands

**Keywords:** Posttraumatic stress disorder, orthopaedic trauma, paediatric, systematic review

## Abstract

**Purpose:**

Posttraumatic stress disorder (PTSD) is a psychiatric disorder that may develop after exposure to severe psychological threats. It is characterized by debilitating symptoms such as re-experiencing and negative changes in mood and cognitions which are associated with comorbidity, functional impairment and increased mortality. Although orthopaedic trauma may be classified as a traumatic experience causing PTSD, the prevalence of PTSD in paediatric patients following orthopaedic trauma is currently unknown.

**Methods:**

This systematic review aimed to obtain insight into the prevalence of PTSD in paediatric patients after orthopaedic trauma (with or without surgical intervention). Seven medical literature databases were searched to identify studies reporting on the occurrence of PTSD in paediatric patients following orthopaedic trauma.

**Results:**

Ten studies were included for analysis. Overall, the pooled prevalence of PTSD was 15% (95% confidence interval: −5.1%, 34.2%), with significant heterogeneity between studies regarding patient population (e.g. age, gender and type of orthopaedic injury) as well as tools used to diagnose PTSD – all influencing the occurrence of PTSD.

**Conclusions:**

As PTSD appears to be a relatively common psychological comorbidity after orthopaedic paediatric trauma, we emphasize that both clinicians and parents should be aware of PTSD symptoms in children to allow early treatment of PTSD.

**Significance of Study:**

Approximately one in seven children may develop PTSD following orthopaedic trauma, highlighting the importance of systematic screening and monitoring of PTSD symptoms in paediatric patients recovering from orthopaedic injuries.

**Level of Evidence:**

Level II (systematic review of level-II studies)

## Introduction

Orthopaedic trauma encompasses any injury to the musculoskeletal system caused by external force, ranging from simple fractures to complex multisystem injuries. Orthopaedic paediatric injuries are relatively common, with incidence rates ranging from 1800 to 5344 per 100,000 persons per year and are impactful in terms of disability, loss of potential, rehabilitation and treatment costs.^1,2^

In addition to physical injury and its functional impact, patients often experience substantial psychological consequences. Orthopaedic trauma can lead to depressive complaints and pain catastrophizing^3–6^ – which, in turn, are associated with increased disability and prolonged recovery times.^7,8^ The psychological burden is particularly concerning in children, whose developmental stage may amplify the emotional impact of trauma. Moreover, the direct experience of a severe accident or violence, with or without physical injury, may lead to posttraumatic stress disorder (PTSD).

PTSD is a severe and often chronic and progressive psychological disorder that develops after exposure to a psychologically traumatic event. Four symptom clusters characterize it: (i) intrusive symptoms (e.g. re-experiencing of recurrent and involuntary distressing memories and dreams), (ii) avoidance of distressing thoughts or external reminders of the trauma, (iii) negative changes in cognition and/or mood and (iv) hyperarousal and hyperreactivity. According to the disorder criteria, PTSD requires symptoms to persist for more than 1 month and cause clinically significant distress or impairment in daily life function.^9^

A previous systematic review and meta-analysis on PTSD after orthopaedic injury in adults showed that the weighted pooled prevalence of PTSD is as high as 27% (range 19.0–35.9%).^10^ However, knowledge on the prevalence of PTSD in paediatric populations following orthopaedic trauma is scarce. In children, developmental factors, such as cognitive maturity and coping mechanisms, may influence psychological outcomes differently compared to adults. A systematic review and meta-analysis investigating the prevalence of psychological stress reactions in children after paediatric surgery found that 16% of children meet the criteria for PTSD post-surgery, underscoring the importance of awareness of medically related PTSD in the paediatric population.^11^ To our best knowledge, no systematic review has been conducted to investigate the prevalence of PTSD in paediatric patients following orthopaedic trauma.

Hence, this study aims to systematically assess the existing literature on PTSD in paediatric patients following orthopaedic trauma. By deriving a pooled estimate, we aim to provide insight into the prevalence of PTSD in this population and to highlight the importance of early psychological assessment and intervention in paediatric orthopaedic care.

## Methods

This review was registered at PROSPERO (registration number: CRD42024545215) prior to data collection and was conducted in accordance with the Preferred Reporting Items for Systematic Reviews (PRISMA) and Meta-Analyses guidelines.^12^

### Search strategy

Seven literature databases (Academic Search Premier, Cochrane Library, Embase, Emcare, PsycINFO, PubMed and Web of Science) were searched for publications using a systematic search strategy developed by an information specialist (JS; see Supplemental Appendix 1). The search consisted of three components: (i) PTSD; (ii) children and (iii) orthopaedic trauma. The list of references was exported to EndNote (version X9, Clarivate Analytics, Philadelphia, United States) to identify and remove duplicate articles. The shortened list was then imported into the web application Rayyan (Doha, Qatar) for study selection.

### Study selection

Two reviewers (JPH and LH) independently screened titles and abstracts before independently assessing the eligibility of full texts. Discrepancies were resolved through discussion. The inclusion criteria were as follows: (i) clinical studies and (ii) reporting on the occurrence of PTSD in children (<18 years old) following orthopaedic trauma (with or without surgical intervention). Articles that focused on patients with injuries sustained in a war context were excluded from analysis, as research indicates that war victims have a higher risk of developing PTSD.^13,14^ Furthermore, case studies and series were excluded from this analysis, as they may misrepresent prevalence numbers due to their inherent selection bias.

### Data extraction

Data were extracted independently by two reviewers (JPH and LH) using an Excel spreadsheet (Microsoft, Redmond, United States) with predefined points of data extraction. The following data were extracted: (i) first author; (ii) year of publication; (iii) country in which the study was conducted; (iv) study design; (v) follow-up duration (months); (vi) number of patients included; (vii) age (mean and range); (viii) gender distribution (% female); (ix) type of orthopaedic injury; (x) type of orthopaedic intervention; (xi) PTSD diagnostic assessment method; and (xii) PTSD occurrence (reported as the percentage of patients with PTSD among all those at risk in each study). All studies were assessed for quality using the Critical Appraisal Skills Program study quality assessment tool.^15^

### Statistical analysis

Statistical analyses were conducted using IBM SPSS Statistics (version 27.0.1). Descriptive statistics were used to summarize study characteristics, including sample size, age, gender distribution, and follow-up duration. Data were presented with means and standard deviations, medians and ranges, or proportions and percentages.

The primary outcome was the prevalence of PTSD in children following orthopaedic trauma. Confidence intervals (CIs) for prevalence rates for studies that did not include the CI in the manuscript were calculated using the Clopper-Pearson interval. For the meta-analysis, weights were first calculated for each study using inverse variance weighting,^16^ and then standardized across studies for pooled prevalence calculations so that their totals equalled 100.

## Results

**Figure 1.**
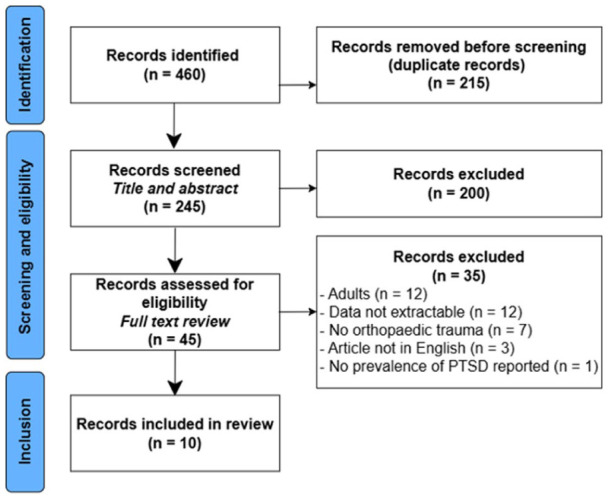
Flowchart of the study selection and inclusion process.

Four hundred and sixty publications were identified using the systematic search. After the removal of duplicates, 245 publications were screened for eligibility based on title and abstract. Thereafter, 45 full texts were assessed, of which 10 studies fulfilled the inclusion criteria (Figure 1). The overall quality of included studies was categorized as ‘fair’ in eight articles, and ‘good’ in two articles (Supplemental Table 1).

Patient demographics of included studies are shown in Table 1: the number of participants per study ranged from 32 to 400 (mean = 136). Overall, 1356 patients with a mean age of 10.2 years (standard deviation = 2.3) were included in our analysis: 32% (*n* = 435) of whom were girls. Regarding orthopaedic trauma, studies differed on how and how detailed these were reported; only five studies reported which limb was affected (i.e. fractured), only two studies reported the number of fractures, and three studies reported the number of patients with open fractures. The occurrence of surgical interventions was described in five studies, but the exact number of patients requiring surgery remains unknown, due to the lack of detailed information.

**Table 1.** Patient demographics of included studies.

Publication	Country	Number of patients	Mean age in years	Orthopaedic injury	Surgical intervention
Al Zomia (2023)^17^	Saudi Arabia	100 (33% female)	7.3 (range 1–12)	1 fracture (*n* = 78); 2 fractures (*n* = 16); 3 fractures (*n* = 3); >3 fractures (*n* = 3)	70 (70%)
Hajek (2009)^18^	United States	99 (37% female)	11.7 (range 8–15)	Fracture with an Abbreviated Injury Scale score of ≤3	Unknown
Levi (1999)^19^	United States	59 (61% female)	9.3 (range 6–12)	Femur fracture (*n* = 28), tibia or fibula fracture (*n* = 7), multiple fractures (*n* = 9)	Unknown
May (2023)^20^	United States	176 (71% female)	13.4 (range 8–18)	Musculoskeletal trauma requiring surgery (*n* = 99) upper extremity (*n* = 78) lower extremity (*n* = 1)	176 (100%)
McKinnon (2017)^21^	Australia	57 (% female unknown)	11.8 (range 7–16)	Trauma after accidental injury	Unknown
Messner (2020)^22^	United Kingdom	32 (38% female)	11 (range 4–17)	Gustilo and Anderson grade IIIB (*n* = 30), or IIIC (*n* = 2) open lower limb fractures	32 (100%) (external fixator *n* = 23, ORIF *n* = 9)
Sanders (2005)^23^	United States	400 (36% female)	11 (range 3–16)	Fractures: radius (*n* = 83), radius and ulna (*n* = 35), closed tibia shaft (*n* = 31), distal radius with ulna (*n* = 23), phalanx (*n* = 19), metacarpal (*n* = 13), ankle (*n* = 11), tibia and fibula (*n* = 11). Ankle sprain (*n* = 11), NB: multisystem injuries (*n* = 23)	Unknown
Subasi (2003)^24^	Turkey	58 (5% female)	7 (range 3–13)	Unstable pelvic fractures Tile type B (*n* = 34) or C1 (*n* = 24) treated non-operatively	0%
Vitale (2006)^25^	United States	299 (40% female)	7.3 (range 5–18)	Ankle (*n* = 36), femur (*n* = 108), supracondylar humerus (*n* = 134), tibial spine (*n* = 3), and open fractures (*n* = 22)	ORIF (*n* = 59), external fixator (*n* = 4), other (*n* = 3), unknown (*n* = 233)
Wallace (2012)^26^	United States	76 (26% female)	12.6 (range 8–17)	High-energy trauma with orthopaedic injuries (*n* = 44) or a low-energy trauma with an isolated upper extremity fracture that was treated nonoperatively (*n* = 32)	32 (42%) (*n* = 22 one surgery; *n* = 10 multiple surgeries)

ORIF: open reduction internal fixation; CI: confidence interval.

Across the studies, six different structured assessment tools were used to assess PTSD (Table 2). Self-report tools were the predominant method of assessment (*n* = 7) while parent-report tools were used in three studies. Only one study performed a clinician-administered interview, albeit unstructured. For the self-report tools, the Child PTSD Symptom Scale (CPSS) was the most frequently used (*n* = 4), while the more concise Child Trauma Screen (CTS), Child PTSD Reaction Index (CPTSDRI) and Child Impact of Events Scale (CRIES) were all used in one study. The follow-up durations varied considerably, ranging from 3 to 89 months. Two studies provided no follow-up duration data.

**Table 2.** Overview of PTSD assessment tools across studies.

Publication	Follow-up (months)	PTSD assessment tool	Type of assessment tool
Al Zomia (2023)^17^	Unknown	CTS	Self-report
Hajek (2009)^18^	12	PCL-C/PR	Parent-report
Levi (1999)^19^	12	CPTSDRI and PTSS parent interview	Self-report and parent-report
May (2023)^20^	6	CPSS	Self-report
McKinnon 2017)^21^	3	CPSS	Self-report
Messner (2020)^22^	65	CRIES	Self-report
Sanders (2005)^23^	3	CPSS	Self-report
Subasi (2003)^24^	Unknown	Unstructured interview using DSM-IV criteria	Clinician-administered
Vitale (2006)^25^	3	PROPS	Parent-report
Wallace (2012)^26^	89	CPSS	Self-report

PTSD: post-traumatic stress disorder; CTS: child trauma screen; PCL-C/PR: PTSD checklist for children/parent report; CPTSDRI: Child post-traumatic stress disorder reaction index; PTSS: post-traumatic stress scale; CPSS: Child PTSD symptom scale; CRIES: Child impact of events scale; DSM-IV: Diagnostic and Statistical Manual of Mental Disorders; PROPS: parent report of posttraumatic symptoms.

The prevalence of PTSD at the last follow-up ranged from 2%^19^ to 38%^22^ and varied by assessment method (Supplemental Table 2). Self-reported outcomes ranged from 5% to 38% (mean = 25%), while the parent-reported outcomes were lower, ranging from 2% to 10% (mean = 8%). The single clinician-assessed study reported 10%, falling between the self- and parent-reported outcomes for PTSD prevalence.

**Figure 2.**
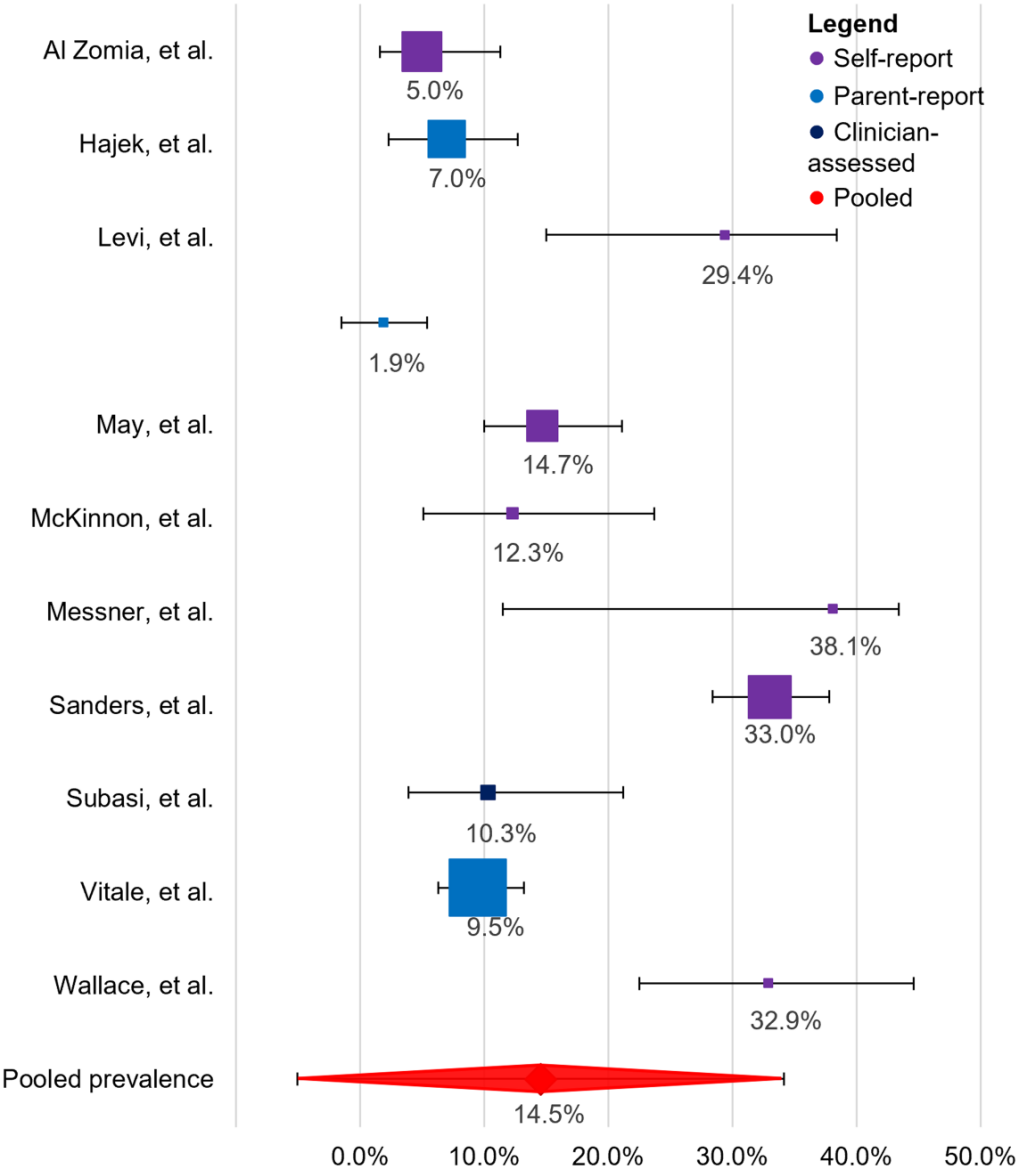
Forest plot of prevalence estimates across included studies. Squares indicate study-specific prevalence estimates, with square size proportional to each study’s weight in the meta-analysis. Horizontal lines represent 95% confidence intervals. The diamond denotes the pooled prevalence estimate and its corresponding 95% confidence interval.

Across the studies, the weighted pooled prevalence was calculated to be 15% (95% CI: −5.1%, 34.2%) (Figure 2). For the study by Levi et al.^19^ the prevalence based on the self-reported outcomes was included in the calculations; the prevalence based on the parent-report was much lower, namely 2% (CI: −1.6, 5.4%).

## Discussion

This systematic review aimed to obtain insight into the prevalence of PTSD in paediatric patients following orthopaedic trauma. In total, 10 studies were included, describing a total of 1356 patients. Given the relatively high prevalence of PTSD (pooled prevalence of 15%), this review underscores the importance of systematic screening and monitoring of PTSD symptoms in paediatric patients following orthopaedic injuries. The notable variability in prevalence estimates between included studies – driven by differences, injury type/severity, orthopaedic treatment, demographics, PTSD assessment tools, and follow-up duration – calls for standardized, developmentally sensitive diagnostic approaches.

Concerning the severity of orthopaedic trauma and the complexity of injuries, Messner et al.^22^ documented a high prevalence of 38% in patients with severe open fractures, whereas Levi et al.^19^ found a substantially lower prevalence of only 2% within a population experiencing less severe injuries. Also, the included studies did not clearly describe the exact type of orthopaedic injuries included, which of these children ultimately underwent surgery, and, finally, which of these children developed PTSD. This information is important, as the literature indicates that hospitalization and surgeries are risk factors for the development of PTSD.^27,28^

Regarding demographics, patient age varied widely across studies. Research has shown that young children’s response to medical trauma varies with age and development, as they often do not perceive their illness or injury as life-threatening due to a limited understanding of death’s permanence.^29–31^ Therefore, the impact of a traumatic event and the subsequent relative risk of developing psychological consequences, such as PTSD, must be considered within the context of their social, emotional, and relational developmental competencies.^32^ Furthermore, gender differences can also affect the prevalence of PTSD, as women are at higher risk than men for developing PTSD.^33–35^ The percentage of girls in the studies ranged from 5% to 71%, potentially affecting the reported PTSD prevalence.

Acknowledging the potential confounding factors of the prevalence rates in the included studies, our findings align with PTSD rates seen in paediatric patients with other traumas, like brain (17%) and spinal cord injuries (24%), and children following hospitalization for any physical illness or injury (22%).^28,36,37^

### Assessment tools for PTSD

A critical insight provided by our review was the marked discrepancy between self-reported (24%) and parent-reported (8%) PTSD prevalence estimates. Previous research consistently highlights parental underestimation of child trauma symptoms, often due to parental coping styles, personal psychological distress or limited recognition of trauma-related behaviours.^11,36^ Therefore, relying on parental reports solely may underestimate child PTSD, risking missed chances for early detection and intervention. In addition, data obtained through self-reports – like those employed in the included studies – are known to be prone to different biases.^37^ Importantly, most self-report measures (i.e. the CTS, CPTSDRI and CRIES) were brief screening or symptom checklists rather than comprehensive diagnostic instruments, which may further influence prevalence estimates. Structured clinical interviews, such as the Clinician-Administered PTSD Scale for Children and Adolescents and the Anxiety Disorders Interview Schedule for Children PTSD module, are widely recognized as the gold standard for PTSD diagnosis in youth due to their systematic assessment of PTSD criteria.^38^ However, structured interviews were not employed in the reviewed studies.

### Assessment timeframe for PTSD

Follow-up durations varied from 3 months^17,20,39^ to 89 months.^21^ Such variations could substantially affect prevalence estimates, given that PTSD symptoms are known to fluctuate, spontaneously remit or manifest as delayed onset beyond typical short-term follow-up periods. A systematic review specifically focusing on children demonstrated that PTSD prevalence tends to decline spontaneously within the first 3 to 6 months following trauma exposure, with only minimal evidence for further spontaneous reductions in prevalence or symptom severity beyond 6 months.^23^ Thus, while spontaneous remission of PTSD symptoms may still occur in the initial months post-trauma, new onset or delayed expressions of PTSD also remain possible. Together, these findings hint that a minimum follow-up period of 6 months should be necessary.

### Clinical implications and recommendations

Recognizing PTSD’s progression following trauma is essential for optimizing clinical care. For children with persistent posttraumatic symptoms, effective psychological interventions, such as cognitive-behavioural therapy, psychoeducation, coping-skills training and peer-support programmes, significantly reduce PTSD severity and facilitate recovery. In addition, treatment is not only crucial for the mental health of patients but also for the orthopaedic care, as PTSD symptoms may interfere with the rehabilitation process. Avoidance and negative alterations may lead to the unwillingness to participate in activities necessary to regain physical function. Hyperarousal may lead to increased irritability and sleep difficulties and, in turn, may exacerbate physical symptoms of pain and discomfort.^25^ Re-experiencing can be triggered by pain or sensory reminders of the trauma, causing a vicious cycle where physical and psychological symptoms reinforce each other.^26,40^

It would be valuable to stratify trauma severity when evaluating the risk of PTSD, as less severe injuries, such as minor fractures, may confer a lower risk compared with more severe trauma, such as polytrauma. However, the available data lacked sufficient detail on injury severity to allow for such stratification in the present analysis. Consequently, further research is required to clarify the relationship between trauma severity and PTSD risk and to inform more precise, severity-based treatment recommendations.

## Conclusions

Approximately one in seven children may develop PTSD following orthopaedic trauma, highlighting the importance of systematic screening and monitoring of PTSD symptoms in paediatric patients recovering from orthopaedic injuries. Variability in prevalence – driven by differences, injury type/severity, orthopaedic treatment, demographics, PTSD assessment tools, and timing of follow-up – emphasizes the need for standardized, developmentally sensitive diagnostic approaches. In addition, findings stress raising awareness among clinicians, fostering collaboration across specialties, and integrating trauma-informed care into treatment. Future research should aim to refine diagnostic tools, establish optimal follow-up durations, and further explore how injury characteristics and treatment contexts contribute to PTSD risk. Addressing these gaps will enhance clinical care and support the long-term well-being of paediatric trauma patients.

## Supplemental Material

sj-doc-1-cho-10.1177_18632521261419773 – Supplemental material for Prevalence of posttraumatic stress disorder in paediatric patients following orthopaedic trauma: A systematic review Supplemental material, sj-doc-1-cho-10.1177_18632521261419773 for Prevalence of posttraumatic stress disorder in paediatric patients following orthopaedic trauma: A systematic review by Janne L Punski-Hoogervorst, Lotje A Hoogervorst, Jan W Schoones, Avi Avital and Pieter Bas de Witte in Journal of Children's Orthopaedics

sj-docx-2-cho-10.1177_18632521261419773 – Supplemental material for Prevalence of posttraumatic stress disorder in paediatric patients following orthopaedic trauma: A systematic review Supplemental material, sj-docx-2-cho-10.1177_18632521261419773 for Prevalence of posttraumatic stress disorder in paediatric patients following orthopaedic trauma: A systematic review by Janne L Punski-Hoogervorst, Lotje A Hoogervorst, Jan W Schoones, Avi Avital and Pieter Bas de Witte in Journal of Children's Orthopaedics

sj-pdf-3-cho-10.1177_18632521261419773 – Supplemental material for Prevalence of posttraumatic stress disorder in paediatric patients following orthopaedic trauma: A systematic review Supplemental material, sj-pdf-3-cho-10.1177_18632521261419773 for Prevalence of posttraumatic stress disorder in paediatric patients following orthopaedic trauma: A systematic review by Janne L Punski-Hoogervorst, Lotje A Hoogervorst, Jan W Schoones, Avi Avital and Pieter Bas de Witte in Journal of Children's Orthopaedics

sj-pdf-4-cho-10.1177_18632521261419773 – Supplemental material for Prevalence of posttraumatic stress disorder in paediatric patients following orthopaedic trauma: A systematic review Supplemental material, sj-pdf-4-cho-10.1177_18632521261419773 for Prevalence of posttraumatic stress disorder in paediatric patients following orthopaedic trauma: A systematic review by Janne L Punski-Hoogervorst, Lotje A Hoogervorst, Jan W Schoones, Avi Avital and Pieter Bas de Witte in Journal of Children's Orthopaedics

sj-pdf-5-cho-10.1177_18632521261419773 – Supplemental material for Prevalence of posttraumatic stress disorder in paediatric patients following orthopaedic trauma: A systematic review Supplemental material, sj-pdf-5-cho-10.1177_18632521261419773 for Prevalence of posttraumatic stress disorder in paediatric patients following orthopaedic trauma: A systematic review by Janne L Punski-Hoogervorst, Lotje A Hoogervorst, Jan W Schoones, Avi Avital and Pieter Bas de Witte in Journal of Children's Orthopaedics

sj-pdf-6-cho-10.1177_18632521261419773 – Supplemental material for Prevalence of posttraumatic stress disorder in paediatric patients following orthopaedic trauma: A systematic review Supplemental material, sj-pdf-6-cho-10.1177_18632521261419773 for Prevalence of posttraumatic stress disorder in paediatric patients following orthopaedic trauma: A systematic review by Janne L Punski-Hoogervorst, Lotje A Hoogervorst, Jan W Schoones, Avi Avital and Pieter Bas de Witte in Journal of Children's Orthopaedics

sj-pdf-7-cho-10.1177_18632521261419773 – Supplemental material for Prevalence of posttraumatic stress disorder in paediatric patients following orthopaedic trauma: A systematic review Supplemental material, sj-pdf-7-cho-10.1177_18632521261419773 for Prevalence of posttraumatic stress disorder in paediatric patients following orthopaedic trauma: A systematic review by Janne L Punski-Hoogervorst, Lotje A Hoogervorst, Jan W Schoones, Avi Avital and Pieter Bas de Witte in Journal of Children's Orthopaedics
