# Supplementary material for: Prevalence of posttraumatic stress disorder in paediatric patients following orthopaedic trauma: A systematic review
Source: J Child Orthop. 2026 Feb 18;20(2):103–10. doi: 10.1177/18632521261419773 (PMC12920159; doi:10.1177/18632521261419773)
Supplement: sj-doc-1-cho-10.1177_18632521261419773 – Supplemental material for Prevalence of posttraumatic stress disorder in paediatric patients following orthopaedic trauma: A systematic review [file sj-doc-1-cho-10.1177_18632521261419773.doc]

**Appendix 1 – Search strategy**

**Academic Search Premier**

((TI("posttraumatic stress disorder" OR "Moral Injuries" OR "Moral Injury" OR "Post Traumatic Neuroses" OR "Post Traumatic Neurosis" OR "Post Traumatic Stress" OR "Post Traumatic Stress Disorder" OR "Post Traumatic Stress Disorders" OR "Posttraumatic Neuroses" OR "Posttraumatic Neurosis" OR "Posttraumatic Stress" OR "Posttraumatic Stress Disorder" OR "Posttraumatic Stress Disorders" OR (("PTSD" NEAR/10 "stress") OR ("PTSS" NEAR/10 "stress")) OR (("Moral" NEAR/6 "Injuries") OR ("Moral" NEAR/6 "Injury") OR ("Post" NEAR/6 "Traumatic" NEAR/6 "Neuroses") OR ("Post" NEAR/6 "Traumatic" NEAR/6 "Neurosis") OR ("Post" NEAR/6 "Traumatic" NEAR/6 "Stress") OR ("Post" NEAR/6 "Traumatic" NEAR/6 "Stress" NEAR/6 "Disorder") OR ("Post" NEAR/6 "Traumatic" NEAR/6 "Stress" NEAR/6 "Disorders") OR ("Posttraumatic" NEAR/6 "Neuroses") OR ("Posttraumatic" NEAR/6 "Neurosis") OR ("Posttraumatic" NEAR/6 "Stress") OR ("Posttraumatic" NEAR/6 "Stress" NEAR/6 "Disorder") OR ("Posttraumatic" NEAR/6 "Stress" NEAR/6 "Disorders"))) OR SU("posttraumatic stress disorder" OR "Moral Injuries" OR "Moral Injury" OR "Post Traumatic Neuroses" OR "Post Traumatic Neurosis" OR "Post Traumatic Stress" OR "Post Traumatic Stress Disorder" OR "Post Traumatic Stress Disorders" OR "Posttraumatic Neuroses" OR "Posttraumatic Neurosis" OR "Posttraumatic Stress" OR "Posttraumatic Stress Disorder" OR "Posttraumatic Stress Disorders" OR (("PTSD" NEAR/10 "stress") OR ("PTSS" NEAR/10 "stress")) OR (("Moral" NEAR/6 "Injuries") OR ("Moral" NEAR/6 "Injury") OR ("Post" NEAR/6 "Traumatic" NEAR/6 "Neuroses") OR ("Post" NEAR/6 "Traumatic" NEAR/6 "Neurosis") OR ("Post" NEAR/6 "Traumatic" NEAR/6 "Stress") OR ("Post" NEAR/6 "Traumatic" NEAR/6 "Stress" NEAR/6 "Disorder") OR ("Post" NEAR/6 "Traumatic" NEAR/6 "Stress" NEAR/6 "Disorders") OR ("Posttraumatic" NEAR/6 "Neuroses") OR ("Posttraumatic" NEAR/6 "Neurosis") OR ("Posttraumatic" NEAR/6 "Stress") OR ("Posttraumatic" NEAR/6 "Stress" NEAR/6 "Disorder") OR ("Posttraumatic" NEAR/6 "Stress" NEAR/6 "Disorders"))) OR KW("posttraumatic stress disorder" OR "Moral Injuries" OR "Moral Injury" OR "Post Traumatic Neuroses" OR "Post Traumatic Neurosis" OR "Post Traumatic Stress" OR "Post Traumatic Stress Disorder" OR "Post Traumatic Stress Disorders" OR "Posttraumatic Neuroses" OR "Posttraumatic Neurosis" OR "Posttraumatic Stress" OR "Posttraumatic Stress Disorder" OR "Posttraumatic Stress Disorders" OR (("PTSD" NEAR/10 "stress") OR ("PTSS" NEAR/10 "stress")) OR (("Moral" NEAR/6 "Injuries") OR ("Moral" NEAR/6 "Injury") OR ("Post" NEAR/6 "Traumatic" NEAR/6 "Neuroses") OR ("Post" NEAR/6 "Traumatic" NEAR/6 "Neurosis") OR ("Post" NEAR/6 "Traumatic" NEAR/6 "Stress") OR ("Post" NEAR/6 "Traumatic" NEAR/6 "Stress" NEAR/6 "Disorder") OR ("Post" NEAR/6 "Traumatic" NEAR/6 "Stress" NEAR/6 "Disorders") OR ("Posttraumatic" NEAR/6 "Neuroses") OR ("Posttraumatic" NEAR/6 "Neurosis") OR ("Posttraumatic" NEAR/6 "Stress") OR ("Posttraumatic" NEAR/6 "Stress" NEAR/6 "Disorder") OR ("Posttraumatic" NEAR/6 "Stress" NEAR/6 "Disorders"))) OR AB("posttraumatic stress disorder" OR "Moral Injuries" OR "Moral Injury" OR "Post Traumatic Neuroses" OR "Post Traumatic Neurosis" OR "Post Traumatic Stress" OR "Post Traumatic Stress Disorder" OR "Post Traumatic Stress Disorders" OR "Posttraumatic Neuroses" OR "Posttraumatic Neurosis" OR "Posttraumatic Stress" OR "Posttraumatic Stress Disorder" OR "Posttraumatic Stress Disorders" OR (("PTSD" NEAR/10 "stress") OR ("PTSS" NEAR/10 "stress")) OR (("Moral" NEAR/6 "Injuries") OR ("Moral" NEAR/6 "Injury") OR ("Post" NEAR/6 "Traumatic" NEAR/6 "Neuroses") OR ("Post" NEAR/6 "Traumatic" NEAR/6 "Neurosis") OR ("Post" NEAR/6 "Traumatic" NEAR/6 "Stress") OR ("Post" NEAR/6 "Traumatic" NEAR/6 "Stress" NEAR/6 "Disorder") OR ("Post" NEAR/6 "Traumatic" NEAR/6 "Stress" NEAR/6 "Disorders") OR ("Posttraumatic" NEAR/6 "Neuroses") OR ("Posttraumatic" NEAR/6 "Neurosis") OR ("Posttraumatic" NEAR/6 "Stress") OR ("Posttraumatic" NEAR/6 "Stress" NEAR/6 "Disorder") OR ("Posttraumatic" NEAR/6 "Stress" NEAR/6 "Disorders")))) AND (TI("child" OR "children" OR exp "Infant" OR "infant" OR "infants" OR "infancy" OR "newborn" OR "newborns" OR "new-born" OR "new-borns" OR "neonate" OR "neonates" OR "neonatal" OR "neo-nate" OR "neo-nates" OR "neo-natal" OR "neonatology" OR "NICU" OR "premature" OR "prematures" OR "pre-mature" OR "pre-matures" OR "preterm" OR "pre-term" OR "postnatal" OR "post-natal" OR "baby" OR "babies" OR "suckling" OR "sucklings" OR "toddler" OR "toddlers" OR "childhood" OR "schoolchild" OR "schoolchildren" OR "childcare" OR "child-care" OR "youngster" OR "youngsters" OR "preschool" OR "pre-school" OR "kid" OR "kids" OR "boy" OR "boys" OR "girl" OR "girls" OR exp "Adolescent" OR "adolescent" OR "adolescents" OR "adolescence" OR "pre-adolescent" OR "pre-adolescents" OR "pre-adolescence" OR "schoolage" OR "schoolboy" OR "schoolboys" OR "schoolgirl" OR "schoolgirls" OR "pre-puber" OR "pre-puberty" OR "prepuber" OR "prepubers" OR "prepuberty" OR "puber" OR "puberty" OR "puberal" OR "teenager" OR "teenagers" OR "teens" OR "youth" OR "youths" OR "underaged" OR "under-aged" OR exp "Pediatrics" OR "Pediatric" OR "Pediatrics" OR "Paediatric" OR "Paediatrics" **OR** "child" OR children* OR schoolchild* OR "infant" OR "infants" OR "infancy" OR adolesc* OR pediat* OR paediat* OR neonat* OR toddler* OR "teen" OR "teens" OR teenager* OR preteen* OR newborn* OR postneonat* OR postnatal* OR "puberty" OR preschool* OR suckling* OR "juvenile" OR "new born" OR "new borns" OR new-born* OR neo-nat* OR neonat* OR perinat* OR underag* OR "under age" OR "under aged" OR youth* OR kinder* OR pubescen* OR prepubescen* OR "prepuberty" OR "school age" OR "schoolage" OR "school ages" OR schoolage*) OR SU("child" OR "children" OR exp "Infant" OR "infant" OR "infants" OR "infancy" OR "newborn" OR "newborns" OR "new-born" OR "new-borns" OR "neonate" OR "neonates" OR "neonatal" OR "neo-nate" OR "neo-nates" OR "neo-natal" OR "neonatology" OR "NICU" OR "premature" OR "prematures" OR "pre-mature" OR "pre-matures" OR "preterm" OR "pre-term" OR "postnatal" OR "post-natal" OR "baby" OR "babies" OR "suckling" OR "sucklings" OR "toddler" OR "toddlers" OR "childhood" OR "schoolchild" OR "schoolchildren" OR "childcare" OR "child-care" OR "youngster" OR "youngsters" OR "preschool" OR "pre-school" OR "kid" OR "kids" OR "boy" OR "boys" OR "girl" OR "girls" OR exp "Adolescent" OR "adolescent" OR "adolescents" OR "adolescence" OR "pre-adolescent" OR "pre-adolescents" OR "pre-adolescence" OR "schoolage" OR "schoolboy" OR "schoolboys" OR "schoolgirl" OR "schoolgirls" OR "pre-puber" OR "pre-puberty" OR "prepuber" OR "prepubers" OR "prepuberty" OR "puber" OR "puberty" OR "puberal" OR "teenager" OR "teenagers" OR "teens" OR "youth" OR "youths" OR "underaged" OR "under-aged" OR exp "Pediatrics" OR "Pediatric" OR "Pediatrics" OR "Paediatric" OR "Paediatrics" **OR** "child" OR children* OR schoolchild* OR "infant" OR "infants" OR "infancy" OR adolesc* OR pediat* OR paediat* OR neonat* OR toddler* OR "teen" OR "teens" OR teenager* OR preteen* OR newborn* OR postneonat* OR postnatal* OR "puberty" OR preschool* OR suckling* OR "juvenile" OR "new born" OR "new borns" OR new-born* OR neo-nat* OR neonat* OR perinat* OR underag* OR "under age" OR "under aged" OR youth* OR kinder* OR pubescen* OR prepubescen* OR "prepuberty" OR "school age" OR "schoolage" OR "school ages" OR schoolage*) OR KW("child" OR "children" OR exp "Infant" OR "infant" OR "infants" OR "infancy" OR "newborn" OR "newborns" OR "new-born" OR "new-borns" OR "neonate" OR "neonates" OR "neonatal" OR "neo-nate" OR "neo-nates" OR "neo-natal" OR "neonatology" OR "NICU" OR "premature" OR "prematures" OR "pre-mature" OR "pre-matures" OR "preterm" OR "pre-term" OR "postnatal" OR "post-natal" OR "baby" OR "babies" OR "suckling" OR "sucklings" OR "toddler" OR "toddlers" OR "childhood" OR "schoolchild" OR "schoolchildren" OR "childcare" OR "child-care" OR "youngster" OR "youngsters" OR "preschool" OR "pre-school" OR "kid" OR "kids" OR "boy" OR "boys" OR "girl" OR "girls" OR exp "Adolescent" OR "adolescent" OR "adolescents" OR "adolescence" OR "pre-adolescent" OR "pre-adolescents" OR "pre-adolescence" OR "schoolage" OR "schoolboy" OR "schoolboys" OR "schoolgirl" OR "schoolgirls" OR "pre-puber" OR "pre-puberty" OR "prepuber" OR "prepubers" OR "prepuberty" OR "puber" OR "puberty" OR "puberal" OR "teenager" OR "teenagers" OR "teens" OR "youth" OR "youths" OR "underaged" OR "under-aged" OR exp "Pediatrics" OR "Pediatric" OR "Pediatrics" OR "Paediatric" OR "Paediatrics" **OR** "child" OR children* OR schoolchild* OR "infant" OR "infants" OR "infancy" OR adolesc* OR pediat* OR paediat* OR neonat* OR toddler* OR "teen" OR "teens" OR teenager* OR preteen* OR newborn* OR postneonat* OR postnatal* OR "puberty" OR preschool* OR suckling* OR "juvenile" OR "new born" OR "new borns" OR new-born* OR neo-nat* OR neonat* OR perinat* OR underag* OR "under age" OR "under aged" OR youth* OR kinder* OR pubescen* OR prepubescen* OR "prepuberty" OR "school age" OR "schoolage" OR "school ages" OR schoolage*) OR AB("child" OR "children" OR exp "Infant" OR "infant" OR "infants" OR "infancy" OR "newborn" OR "newborns" OR "new-born" OR "new-borns" OR "neonate" OR "neonates" OR "neonatal" OR "neo-nate" OR "neo-nates" OR "neo-natal" OR "neonatology" OR "NICU" OR "premature" OR "prematures" OR "pre-mature" OR "pre-matures" OR "preterm" OR "pre-term" OR "postnatal" OR "post-natal" OR "baby" OR "babies" OR "suckling" OR "sucklings" OR "toddler" OR "toddlers" OR "childhood" OR "schoolchild" OR "schoolchildren" OR "childcare" OR "child-care" OR "youngster" OR "youngsters" OR "preschool" OR "pre-school" OR "kid" OR "kids" OR "boy" OR "boys" OR "girl" OR "girls" OR exp "Adolescent" OR "adolescent" OR "adolescents" OR "adolescence" OR "pre-adolescent" OR "pre-adolescents" OR "pre-adolescence" OR "schoolage" OR "schoolboy" OR "schoolboys" OR "schoolgirl" OR "schoolgirls" OR "pre-puber" OR "pre-puberty" OR "prepuber" OR "prepubers" OR "prepuberty" OR "puber" OR "puberty" OR "puberal" OR "teenager" OR "teenagers" OR "teens" OR "youth" OR "youths" OR "underaged" OR "under-aged" OR exp "Pediatrics" OR "Pediatric" OR "Pediatrics" OR "Paediatric" OR "Paediatrics" **OR** "child" OR children* OR schoolchild* OR "infant" OR "infants" OR "infancy" OR adolesc* OR pediat* OR paediat* OR neonat* OR toddler* OR "teen" OR "teens" OR teenager* OR preteen* OR newborn* OR postneonat* OR postnatal* OR "puberty" OR preschool* OR suckling* OR "juvenile" OR "new born" OR "new borns" OR new-born* OR neo-nat* OR neonat* OR perinat* OR underag* OR "under age" OR "under aged" OR youth* OR kinder* OR pubescen* OR prepubescen* OR "prepuberty" OR "school age" OR "schoolage" OR "school ages" OR schoolage*) OR TI("one year old" OR "two year old" OR "three year old" OR "four year old" OR "five year old" OR "six year old" OR "seven year old" OR "eight year old" OR "nine year old" OR "ten year old" OR "eleven year old" OR "twelve year old" OR "thirteen year old" OR "fourteen year old" OR "fifteen year old" OR "sixteen year old" OR "seventeen year old" OR "eighteen year old" OR "1 year old" OR "2 year old" OR "3 year old" OR "4 year old" OR "5 year old" OR "6 year old" OR "7 year old" OR "8 year old" OR "9 year old" OR "10 year old" OR "11 year old" OR "12 year old" OR "13 year old" OR "14 year old" OR "15 year old" OR "16 year old" OR "17 year old" OR "18 year old" OR "two years old" OR "three years old" OR "four years old" OR "five years old" OR "six years old" OR "seven years old" OR "eight years old" OR "nine years old" OR "ten years old" OR "eleven years old" OR "twelve years old" OR "thirteen years old" OR "fourteen years old" OR "fifteen years old" OR "sixteen years old" OR "seventeen years old" OR "eighteen years old" OR "2 years old" OR "3 years old" OR "4 years old" OR "5 years old" OR "6 years old" OR "7 years old" OR "8 years old" OR "9 years old" OR "10 years old" OR "11 years old" OR "12 years old" OR "13 years old" OR "14 years old" OR "15 years old" OR "16 years old" OR "17 years old" OR "18 years old")) AND (TI("orthopedic trauma" OR "orthopedic traumas" OR "orthopaedic trauma" OR "orthopaedic traumas" OR "orthopedic injury" OR "orthopedic injuries" OR "orthopaedic injury" OR "orthopaedic injuries" OR "orthopedic surgery" OR "orthopedic surgeries" OR "orthopaedic surgery" OR "orthopaedic surgeries" OR "orthopedic intervention" OR "orthopedic interventions" OR "orthopaedic intervention" OR "orthopaedic interventions" OR "orthopedic trauma" OR "orthopedic procedures" OR "orthopaedic procedure" OR "orthopaedic procedures" OR "Arm Injury" OR "Back Injury" OR "Fracture" OR "Hand Injury" OR "Hip Injury" OR "Joint Dislocations" OR "Leg Injury" OR "Neck Injury" OR "Shoulder Injury" OR "Sprain" OR "Tendon Injury" OR "Ankle Fractures" OR "Ankle Injuries" OR "Arm Injuries" OR "Back Injuries" OR "Bone Diastasis" OR "Elbow Fractures" OR "Elbow Injuries" OR "Femoral Fractures" OR "Fibula Fractures" OR "Finger Injuries" OR "Foot Injuries" OR "Forearm Injuries" OR "Fracture" OR "Fracture Dislocation" OR "Fractures" OR "Hand Injuries" OR "Hip Dislocation" OR "Hip Fractures" OR "Hip Injuries" OR "Humeral Fractures" OR "Intra-Articular Fractures" OR "Joint Dislocations" OR "Knee Dislocation" OR "Knee Fractures" OR "Knee Injuries" OR "Leg Injuries" OR "Medial Tibial Stress Syndrome" OR "Muscle Diastasis" OR "Neck Injuries" OR "Osteoporotic Fractures" OR "Patellar Dislocation" OR "Periprosthetic Fractures" OR "Radius Fractures" OR "Rib Fractures" OR "Rotator Cuff Injuries" OR "Shoulder Dislocation" OR "Shoulder Fractures" OR "Shoulder Impingement Syndrome" OR "Shoulder Injuries" OR "Skull Fractures" OR "Spinal Fractures" OR "Spinal Injuries" OR "Sprain" OR "Tendinopathy" OR "Tendon Injuries" OR "Tibial Fractures" OR "Tibial Meniscus Injuries" OR "Traumatic Amputation" OR "Traumatic Multiple Amputations" OR "Ulna Fractures" OR "Whiplash Injuries" OR "Wrist Fractures" OR "Wrist Injuries" OR "Ankle Fracture" OR "Ankle Injury" OR "Arm Injury" OR "Back Injury" OR "Elbow Fracture" OR "Elbow Injury" OR "Femoral Fracture" OR "Fibula Fracture" OR "Finger Injury" OR "Foot Injury" OR "Forearm Injury" OR "Fracture Dislocations" OR "Hand Injury" OR "Hip Dislocations" OR "Hip Fracture" OR "Hip Injury" OR "Humeral Fracture" OR "Humeral Fracture" OR "Intra-Articular Fracture" OR "Joint Dislocation" OR "Knee Dislocations" OR "Knee Fracture" OR "Knee Injury" OR "Leg Injury" OR "Neck Injury" OR "Osteoporotic Fracture" OR "Patellar Dislocations" OR "Periprosthetic Fracture" OR "Radius Fracture" OR "Rib Fracture" OR "Rotator Cuff Injury" OR "Shoulder Dislocations" OR "Shoulder Fracture" OR "Shoulder Impingement" OR "Shoulder Injury" OR "Skull Fracture" OR "Spinal Fracture" OR "Spinal Injury" OR "Sprains" OR "Tendinopathies" OR "Tendon Injury" OR "Tibial Fracture" OR "Tibial Meniscus Injury" OR "Traumatic Amputations" OR "Traumatic Multiple Amputation" OR "Ulna Fracture" OR "Whiplash Injury" OR "Wrist Fracture" OR "Wrist Injury" OR "Orthopedics" OR "Orthopedic Surgery" OR "Acetabuloplast*" OR "Acetabuloplasty" OR "Alveolar Bone Graft*" OR "Alveolar Bone Grafting" OR "Ankle Replacement" OR "Anterior Cruciate Ligament Reconstruction" OR "Arthrodesis" OR "Arthroplast*" OR "Arthroplasty" OR "Arthroscop*" OR "Arthroscopy" OR "Bone Lengthening" OR "Bone Transplant*" OR "Bone Transplantation" OR "Bone-Patellar Tendon-Bone Graft*" OR "Bone-Patellar Tendon-Bone Grafting" OR "Cementoplast*" OR "Cementoplasty" OR "Disarticulation" OR "Diskectom*" OR "Diskectomy" OR "Distraction Osteogenesis" OR "Elbow Replacement" OR "Finger Replacement" OR "Fracture Fixation" OR "Genioplast*" OR "Genioplasty" OR "Hemiarthroplast*" OR "Hemiarthroplasty" OR "Hemipelvectom*" OR "Hemipelvectomy" OR "Hip Replacement" OR "Ilizarov Technique" OR "Joint Capsule Release" OR "Knee Replacement" OR "Kyphoplast*" OR "Kyphoplasty" OR "Laminectom*" OR "Laminectomy" OR "Le Fort Osteotom*" OR "Le Fort Osteotomy" OR "Limb Salvage" OR "Mandibular Osteotom*" OR "Mandibular Osteotomy" OR "Mandibular Reconstruction" OR "Maxillary Osteotom*" OR "Maxillary Osteotomy" OR "Meniscectom*" OR "Meniscectomy" OR "Open Fracture Reduction" OR "Orthognathic Surgical Procedure" OR "Orthognathic Surgical Procedures" OR "Osteotom*" OR "Osteotomy" OR "Posterior Cruciate Ligament Reconstruction" OR "Replacement Arthroplast*" OR "Replacement Arthroplasty" OR "Sagittal Split Ramus Osteotom*" OR "Sagittal Split Ramus Osteotomy" OR "Shoulder Replacement" OR "Sinus Floor Augmentation" OR "Spinal Fusion" OR "Surgical Amputation" OR "Synovectom*" OR "Synovectomy" OR "Tendon Transfer" OR "Tenodesis" OR "Tenotom*" OR "Tenotomy" OR "Total Disc Replacement" OR "Traction" OR "Ulnar Collateral Ligament Reconstruction" OR "Vertebroplast*" OR "Vertebroplasty" OR "Orthopedic Equipment" OR "orthopedic" OR "orthopedics" OR "orthopedic*" OR "orthopaedic" OR "orthopaedics" OR "orthopaedic*") OR SU("orthopedic trauma" OR "orthopedic traumas" OR "orthopaedic trauma" OR "orthopaedic traumas" OR "orthopedic injury" OR "orthopedic injuries" OR "orthopaedic injury" OR "orthopaedic injuries" OR "orthopedic surgery" OR "orthopedic surgeries" OR "orthopaedic surgery" OR "orthopaedic surgeries" OR "orthopedic intervention" OR "orthopedic interventions" OR "orthopaedic intervention" OR "orthopaedic interventions" OR "orthopedic trauma" OR "orthopedic procedures" OR "orthopaedic procedure" OR "orthopaedic procedures" OR "Arm Injury" OR "Back Injury" OR "Fracture" OR "Hand Injury" OR "Hip Injury" OR "Joint Dislocations" OR "Leg Injury" OR "Neck Injury" OR "Shoulder Injury" OR "Sprain" OR "Tendon Injury" OR "Ankle Fractures" OR "Ankle Injuries" OR "Arm Injuries" OR "Back Injuries" OR "Bone Diastasis" OR "Elbow Fractures" OR "Elbow Injuries" OR "Femoral Fractures" OR "Fibula Fractures" OR "Finger Injuries" OR "Foot Injuries" OR "Forearm Injuries" OR "Fracture" OR "Fracture Dislocation" OR "Fractures" OR "Hand Injuries" OR "Hip Dislocation" OR "Hip Fractures" OR "Hip Injuries" OR "Humeral Fractures" OR "Intra-Articular Fractures" OR "Joint Dislocations" OR "Knee Dislocation" OR "Knee Fractures" OR "Knee Injuries" OR "Leg Injuries" OR "Medial Tibial Stress Syndrome" OR "Muscle Diastasis" OR "Neck Injuries" OR "Osteoporotic Fractures" OR "Patellar Dislocation" OR "Periprosthetic Fractures" OR "Radius Fractures" OR "Rib Fractures" OR "Rotator Cuff Injuries" OR "Shoulder Dislocation" OR "Shoulder Fractures" OR "Shoulder Impingement Syndrome" OR "Shoulder Injuries" OR "Skull Fractures" OR "Spinal Fractures" OR "Spinal Injuries" OR "Sprain" OR "Tendinopathy" OR "Tendon Injuries" OR "Tibial Fractures" OR "Tibial Meniscus Injuries" OR "Traumatic Amputation" OR "Traumatic Multiple Amputations" OR "Ulna Fractures" OR "Whiplash Injuries" OR "Wrist Fractures" OR "Wrist Injuries" OR "Ankle Fracture" OR "Ankle Injury" OR "Arm Injury" OR "Back Injury" OR "Elbow Fracture" OR "Elbow Injury" OR "Femoral Fracture" OR "Fibula Fracture" OR "Finger Injury" OR "Foot Injury" OR "Forearm Injury" OR "Fracture Dislocations" OR "Hand Injury" OR "Hip Dislocations" OR "Hip Fracture" OR "Hip Injury" OR "Humeral Fracture" OR "Humeral Fracture" OR "Intra-Articular Fracture" OR "Joint Dislocation" OR "Knee Dislocations" OR "Knee Fracture" OR "Knee Injury" OR "Leg Injury" OR "Neck Injury" OR "Osteoporotic Fracture" OR "Patellar Dislocations" OR "Periprosthetic Fracture" OR "Radius Fracture" OR "Rib Fracture" OR "Rotator Cuff Injury" OR "Shoulder Dislocations" OR "Shoulder Fracture" OR "Shoulder Impingement" OR "Shoulder Injury" OR "Skull Fracture" OR "Spinal Fracture" OR "Spinal Injury" OR "Sprains" OR "Tendinopathies" OR "Tendon Injury" OR "Tibial Fracture" OR "Tibial Meniscus Injury" OR "Traumatic Amputations" OR "Traumatic Multiple Amputation" OR "Ulna Fracture" OR "Whiplash Injury" OR "Wrist Fracture" OR "Wrist Injury" OR "Orthopedics" OR "Orthopedic Surgery" OR "Acetabuloplast*" OR "Acetabuloplasty" OR "Alveolar Bone Graft*" OR "Alveolar Bone Grafting" OR "Ankle Replacement" OR "Anterior Cruciate Ligament Reconstruction" OR "Arthrodesis" OR "Arthroplast*" OR "Arthroplasty" OR "Arthroscop*" OR "Arthroscopy" OR "Bone Lengthening" OR "Bone Transplant*" OR "Bone Transplantation" OR "Bone-Patellar Tendon-Bone Graft*" OR "Bone-Patellar Tendon-Bone Grafting" OR "Cementoplast*" OR "Cementoplasty" OR "Disarticulation" OR "Diskectom*" OR "Diskectomy" OR "Distraction Osteogenesis" OR "Elbow Replacement" OR "Finger Replacement" OR "Fracture Fixation" OR "Genioplast*" OR "Genioplasty" OR "Hemiarthroplast*" OR "Hemiarthroplasty" OR "Hemipelvectom*" OR "Hemipelvectomy" OR "Hip Replacement" OR "Ilizarov Technique" OR "Joint Capsule Release" OR "Knee Replacement" OR "Kyphoplast*" OR "Kyphoplasty" OR "Laminectom*" OR "Laminectomy" OR "Le Fort Osteotom*" OR "Le Fort Osteotomy" OR "Limb Salvage" OR "Mandibular Osteotom*" OR "Mandibular Osteotomy" OR "Mandibular Reconstruction" OR "Maxillary Osteotom*" OR "Maxillary Osteotomy" OR "Meniscectom*" OR "Meniscectomy" OR "Open Fracture Reduction" OR "Orthognathic Surgical Procedure" OR "Orthognathic Surgical Procedures" OR "Osteotom*" OR "Osteotomy" OR "Posterior Cruciate Ligament Reconstruction" OR "Replacement Arthroplast*" OR "Replacement Arthroplasty" OR "Sagittal Split Ramus Osteotom*" OR "Sagittal Split Ramus Osteotomy" OR "Shoulder Replacement" OR "Sinus Floor Augmentation" OR "Spinal Fusion" OR "Surgical Amputation" OR "Synovectom*" OR "Synovectomy" OR "Tendon Transfer" OR "Tenodesis" OR "Tenotom*" OR "Tenotomy" OR "Total Disc Replacement" OR "Traction" OR "Ulnar Collateral Ligament Reconstruction" OR "Vertebroplast*" OR "Vertebroplasty" OR "Orthopedic Equipment" OR "orthopedic" OR "orthopedics" OR "orthopedic*" OR "orthopaedic" OR "orthopaedics" OR "orthopaedic*") OR KW("orthopedic trauma" OR "orthopedic traumas" OR "orthopaedic trauma" OR "orthopaedic traumas" OR "orthopedic injury" OR "orthopedic injuries" OR "orthopaedic injury" OR "orthopaedic injuries" OR "orthopedic surgery" OR "orthopedic surgeries" OR "orthopaedic surgery" OR "orthopaedic surgeries" OR "orthopedic intervention" OR "orthopedic interventions" OR "orthopaedic intervention" OR "orthopaedic interventions" OR "orthopedic trauma" OR "orthopedic procedures" OR "orthopaedic procedure" OR "orthopaedic procedures" OR "Arm Injury" OR "Back Injury" OR "Fracture" OR "Hand Injury" OR "Hip Injury" OR "Joint Dislocations" OR "Leg Injury" OR "Neck Injury" OR "Shoulder Injury" OR "Sprain" OR "Tendon Injury" OR "Ankle Fractures" OR "Ankle Injuries" OR "Arm Injuries" OR "Back Injuries" OR "Bone Diastasis" OR "Elbow Fractures" OR "Elbow Injuries" OR "Femoral Fractures" OR "Fibula Fractures" OR "Finger Injuries" OR "Foot Injuries" OR "Forearm Injuries" OR "Fracture" OR "Fracture Dislocation" OR "Fractures" OR "Hand Injuries" OR "Hip Dislocation" OR "Hip Fractures" OR "Hip Injuries" OR "Humeral Fractures" OR "Intra-Articular Fractures" OR "Joint Dislocations" OR "Knee Dislocation" OR "Knee Fractures" OR "Knee Injuries" OR "Leg Injuries" OR "Medial Tibial Stress Syndrome" OR "Muscle Diastasis" OR "Neck Injuries" OR "Osteoporotic Fractures" OR "Patellar Dislocation" OR "Periprosthetic Fractures" OR "Radius Fractures" OR "Rib Fractures" OR "Rotator Cuff Injuries" OR "Shoulder Dislocation" OR "Shoulder Fractures" OR "Shoulder Impingement Syndrome" OR "Shoulder Injuries" OR "Skull Fractures" OR "Spinal Fractures" OR "Spinal Injuries" OR "Sprain" OR "Tendinopathy" OR "Tendon Injuries" OR "Tibial Fractures" OR "Tibial Meniscus Injuries" OR "Traumatic Amputation" OR "Traumatic Multiple Amputations" OR "Ulna Fractures" OR "Whiplash Injuries" OR "Wrist Fractures" OR "Wrist Injuries" OR "Ankle Fracture" OR "Ankle Injury" OR "Arm Injury" OR "Back Injury" OR "Elbow Fracture" OR "Elbow Injury" OR "Femoral Fracture" OR "Fibula Fracture" OR "Finger Injury" OR "Foot Injury" OR "Forearm Injury" OR "Fracture Dislocations" OR "Hand Injury" OR "Hip Dislocations" OR "Hip Fracture" OR "Hip Injury" OR "Humeral Fracture" OR "Humeral Fracture" OR "Intra-Articular Fracture" OR "Joint Dislocation" OR "Knee Dislocations" OR "Knee Fracture" OR "Knee Injury" OR "Leg Injury" OR "Neck Injury" OR "Osteoporotic Fracture" OR "Patellar Dislocations" OR "Periprosthetic Fracture" OR "Radius Fracture" OR "Rib Fracture" OR "Rotator Cuff Injury" OR "Shoulder Dislocations" OR "Shoulder Fracture" OR "Shoulder Impingement" OR "Shoulder Injury" OR "Skull Fracture" OR "Spinal Fracture" OR "Spinal Injury" OR "Sprains" OR "Tendinopathies" OR "Tendon Injury" OR "Tibial Fracture" OR "Tibial Meniscus Injury" OR "Traumatic Amputations" OR "Traumatic Multiple Amputation" OR "Ulna Fracture" OR "Whiplash Injury" OR "Wrist Fracture" OR "Wrist Injury" OR "Orthopedics" OR "Orthopedic Surgery" OR "Acetabuloplast*" OR "Acetabuloplasty" OR "Alveolar Bone Graft*" OR "Alveolar Bone Grafting" OR "Ankle Replacement" OR "Anterior Cruciate Ligament Reconstruction" OR "Arthrodesis" OR "Arthroplast*" OR "Arthroplasty" OR "Arthroscop*" OR "Arthroscopy" OR "Bone Lengthening" OR "Bone Transplant*" OR "Bone Transplantation" OR "Bone-Patellar Tendon-Bone Graft*" OR "Bone-Patellar Tendon-Bone Grafting" OR "Cementoplast*" OR "Cementoplasty" OR "Disarticulation" OR "Diskectom*" OR "Diskectomy" OR "Distraction Osteogenesis" OR "Elbow Replacement" OR "Finger Replacement" OR "Fracture Fixation" OR "Genioplast*" OR "Genioplasty" OR "Hemiarthroplast*" OR "Hemiarthroplasty" OR "Hemipelvectom*" OR "Hemipelvectomy" OR "Hip Replacement" OR "Ilizarov Technique" OR "Joint Capsule Release" OR "Knee Replacement" OR "Kyphoplast*" OR "Kyphoplasty" OR "Laminectom*" OR "Laminectomy" OR "Le Fort Osteotom*" OR "Le Fort Osteotomy" OR "Limb Salvage" OR "Mandibular Osteotom*" OR "Mandibular Osteotomy" OR "Mandibular Reconstruction" OR "Maxillary Osteotom*" OR "Maxillary Osteotomy" OR "Meniscectom*" OR "Meniscectomy" OR "Open Fracture Reduction" OR "Orthognathic Surgical Procedure" OR "Orthognathic Surgical Procedures" OR "Osteotom*" OR "Osteotomy" OR "Posterior Cruciate Ligament Reconstruction" OR "Replacement Arthroplast*" OR "Replacement Arthroplasty" OR "Sagittal Split Ramus Osteotom*" OR "Sagittal Split Ramus Osteotomy" OR "Shoulder Replacement" OR "Sinus Floor Augmentation" OR "Spinal Fusion" OR "Surgical Amputation" OR "Synovectom*" OR "Synovectomy" OR "Tendon Transfer" OR "Tenodesis" OR "Tenotom*" OR "Tenotomy" OR "Total Disc Replacement" OR "Traction" OR "Ulnar Collateral Ligament Reconstruction" OR "Vertebroplast*" OR "Vertebroplasty" OR "Orthopedic Equipment" OR "orthopedic" OR "orthopedics" OR "orthopedic*" OR "orthopaedic" OR "orthopaedics" OR "orthopaedic*") OR AB("orthopedic trauma" OR "orthopedic traumas" OR "orthopaedic trauma" OR "orthopaedic traumas" OR "orthopedic injury" OR "orthopedic injuries" OR "orthopaedic injury" OR "orthopaedic injuries" OR "orthopedic surgery" OR "orthopedic surgeries" OR "orthopaedic surgery" OR "orthopaedic surgeries" OR "orthopedic intervention" OR "orthopedic interventions" OR "orthopaedic intervention" OR "orthopaedic interventions" OR "orthopedic trauma" OR "orthopedic procedures" OR "orthopaedic procedure" OR "orthopaedic procedures" OR "Arm Injury" OR "Back Injury" OR "Fracture" OR "Hand Injury" OR "Hip Injury" OR "Joint Dislocations" OR "Leg Injury" OR "Neck Injury" OR "Shoulder Injury" OR "Sprain" OR "Tendon Injury" OR "Ankle Fractures" OR "Ankle Injuries" OR "Arm Injuries" OR "Back Injuries" OR "Bone Diastasis" OR "Elbow Fractures" OR "Elbow Injuries" OR "Femoral Fractures" OR "Fibula Fractures" OR "Finger Injuries" OR "Foot Injuries" OR "Forearm Injuries" OR "Fracture" OR "Fracture Dislocation" OR "Fractures" OR "Hand Injuries" OR "Hip Dislocation" OR "Hip Fractures" OR "Hip Injuries" OR "Humeral Fractures" OR "Intra-Articular Fractures" OR "Joint Dislocations" OR "Knee Dislocation" OR "Knee Fractures" OR "Knee Injuries" OR "Leg Injuries" OR "Medial Tibial Stress Syndrome" OR "Muscle Diastasis" OR "Neck Injuries" OR "Osteoporotic Fractures" OR "Patellar Dislocation" OR "Periprosthetic Fractures" OR "Radius Fractures" OR "Rib Fractures" OR "Rotator Cuff Injuries" OR "Shoulder Dislocation" OR "Shoulder Fractures" OR "Shoulder Impingement Syndrome" OR "Shoulder Injuries" OR "Skull Fractures" OR "Spinal Fractures" OR "Spinal Injuries" OR "Sprain" OR "Tendinopathy" OR "Tendon Injuries" OR "Tibial Fractures" OR "Tibial Meniscus Injuries" OR "Traumatic Amputation" OR "Traumatic Multiple Amputations" OR "Ulna Fractures" OR "Whiplash Injuries" OR "Wrist Fractures" OR "Wrist Injuries" OR "Ankle Fracture" OR "Ankle Injury" OR "Arm Injury" OR "Back Injury" OR "Elbow Fracture" OR "Elbow Injury" OR "Femoral Fracture" OR "Fibula Fracture" OR "Finger Injury" OR "Foot Injury" OR "Forearm Injury" OR "Fracture Dislocations" OR "Hand Injury" OR "Hip Dislocations" OR "Hip Fracture" OR "Hip Injury" OR "Humeral Fracture" OR "Humeral Fracture" OR "Intra-Articular Fracture" OR "Joint Dislocation" OR "Knee Dislocations" OR "Knee Fracture" OR "Knee Injury" OR "Leg Injury" OR "Neck Injury" OR "Osteoporotic Fracture" OR "Patellar Dislocations" OR "Periprosthetic Fracture" OR "Radius Fracture" OR "Rib Fracture" OR "Rotator Cuff Injury" OR "Shoulder Dislocations" OR "Shoulder Fracture" OR "Shoulder Impingement" OR "Shoulder Injury" OR "Skull Fracture" OR "Spinal Fracture" OR "Spinal Injury" OR "Sprains" OR "Tendinopathies" OR "Tendon Injury" OR "Tibial Fracture" OR "Tibial Meniscus Injury" OR "Traumatic Amputations" OR "Traumatic Multiple Amputation" OR "Ulna Fracture" OR "Whiplash Injury" OR "Wrist Fracture" OR "Wrist Injury" OR "Orthopedics" OR "Orthopedic Surgery" OR "Acetabuloplast*" OR "Acetabuloplasty" OR "Alveolar Bone Graft*" OR "Alveolar Bone Grafting" OR "Ankle Replacement" OR "Anterior Cruciate Ligament Reconstruction" OR "Arthrodesis" OR "Arthroplast*" OR "Arthroplasty" OR "Arthroscop*" OR "Arthroscopy" OR "Bone Lengthening" OR "Bone Transplant*" OR "Bone Transplantation" OR "Bone-Patellar Tendon-Bone Graft*" OR "Bone-Patellar Tendon-Bone Grafting" OR "Cementoplast*" OR "Cementoplasty" OR "Disarticulation" OR "Diskectom*" OR "Diskectomy" OR "Distraction Osteogenesis" OR "Elbow Replacement" OR "Finger Replacement" OR "Fracture Fixation" OR "Genioplast*" OR "Genioplasty" OR "Hemiarthroplast*" OR "Hemiarthroplasty" OR "Hemipelvectom*" OR "Hemipelvectomy" OR "Hip Replacement" OR "Ilizarov Technique" OR "Joint Capsule Release" OR "Knee Replacement" OR "Kyphoplast*" OR "Kyphoplasty" OR "Laminectom*" OR "Laminectomy" OR "Le Fort Osteotom*" OR "Le Fort Osteotomy" OR "Limb Salvage" OR "Mandibular Osteotom*" OR "Mandibular Osteotomy" OR "Mandibular Reconstruction" OR "Maxillary Osteotom*" OR "Maxillary Osteotomy" OR "Meniscectom*" OR "Meniscectomy" OR "Open Fracture Reduction" OR "Orthognathic Surgical Procedure" OR "Orthognathic Surgical Procedures" OR "Osteotom*" OR "Osteotomy" OR "Posterior Cruciate Ligament Reconstruction" OR "Replacement Arthroplast*" OR "Replacement Arthroplasty" OR "Sagittal Split Ramus Osteotom*" OR "Sagittal Split Ramus Osteotomy" OR "Shoulder Replacement" OR "Sinus Floor Augmentation" OR "Spinal Fusion" OR "Surgical Amputation" OR "Synovectom*" OR "Synovectomy" OR "Tendon Transfer" OR "Tenodesis" OR "Tenotom*" OR "Tenotomy" OR "Total Disc Replacement" OR "Traction" OR "Ulnar Collateral Ligament Reconstruction" OR "Vertebroplast*" OR "Vertebroplasty" OR "Orthopedic Equipment" OR "orthopedic" OR "orthopedics" OR "orthopedic*" OR "orthopaedic" OR "orthopaedics" OR "orthopaedic*")))

**Cochrane Library**

(("posttraumatic stress disorder" OR "Moral Injuries" OR "Moral Injury" OR "Post Traumatic Neuroses" OR "Post Traumatic Neurosis" OR "Post Traumatic Stress" OR "Post Traumatic Stress Disorder" OR "Post Traumatic Stress Disorders" OR "Posttraumatic Neuroses" OR "Posttraumatic Neurosis" OR "Posttraumatic Stress" OR "Posttraumatic Stress Disorder" OR "Posttraumatic Stress Disorders" OR (("PTSD" NEAR/10 "stress") OR ("PTSS" NEAR/10 "stress")) OR (("Moral" NEAR/6 "Injuries") OR ("Moral" NEAR/6 "Injury") OR ("Post" NEAR/6 "Traumatic" NEAR/6 "Neuroses") OR ("Post" NEAR/6 "Traumatic" NEAR/6 "Neurosis") OR ("Post" NEAR/6 "Traumatic" NEAR/6 "Stress") OR ("Post" NEAR/6 "Traumatic" NEAR/6 "Stress" NEAR/6 "Disorder") OR ("Post" NEAR/6 "Traumatic" NEAR/6 "Stress" NEAR/6 "Disorders") OR ("Posttraumatic" NEAR/6 "Neuroses") OR ("Posttraumatic" NEAR/6 "Neurosis") OR ("Posttraumatic" NEAR/6 "Stress") OR ("Posttraumatic" NEAR/6 "Stress" NEAR/6 "Disorder") OR ("Posttraumatic" NEAR/6 "Stress" NEAR/6 "Disorders"))):ti,ab,kw AND (("Child" OR "child" OR "children" OR exp "Infant" OR "infant" OR "infants" OR "infancy" OR "newborn" OR "newborns" OR "new-born" OR "new-borns" OR "neonate" OR "neonates" OR "neonatal" OR "neo-nate" OR "neo-nates" OR "neo-natal" OR "neonatology" OR "NICU" OR "premature" OR "prematures" OR "pre-mature" OR "pre-matures" OR "preterm" OR "pre-term" OR "postnatal" OR "post-natal" OR "baby" OR "babies" OR "suckling" OR "sucklings" OR "toddler" OR "toddlers" OR "childhood" OR "schoolchild" OR "schoolchildren" OR "childcare" OR "child-care" OR "youngster" OR "youngsters" OR "preschool" OR "pre-school" OR "kid" OR "kids" OR "boy" OR "boys" OR "girl" OR "girls" OR exp "Adolescent" OR "adolescent" OR "adolescents" OR "adolescence" OR "pre-adolescent" OR "pre-adolescents" OR "pre-adolescence" OR "schoolage" OR "schoolboy" OR "schoolboys" OR "schoolgirl" OR "schoolgirls" OR "pre-puber" OR "pre-puberty" OR "prepuber" OR "prepubers" OR "prepuberty" OR "puber" OR "puberty" OR "puberal" OR "teenager" OR "teenagers" OR "teens" OR "youth" OR "youths" OR "underaged" OR "under-aged" OR exp "Pediatrics" OR "Pediatric" OR "Pediatrics" OR "Paediatric" OR "Paediatrics" **OR** "child" OR children* OR schoolchild* OR "infant" OR "infants" OR "infancy" OR adolesc* OR pediat* OR paediat* OR neonat* OR toddler* OR "teen" OR "teens" OR teenager* OR preteen* OR newborn* OR postneonat* OR postnatal* OR "puberty" OR preschool* OR suckling* OR "juvenile" OR "new born" OR "new borns" OR new-born* OR neo-nat* OR neonat* OR perinat* OR underag* OR "under age" OR "under aged" OR youth* OR kinder* OR pubescen* OR prepubescen* OR "prepuberty" OR "school age" OR "schoolage" OR "school ages" OR schoolage*):ti,ab,kw OR ("one year old" OR "two year old" OR "three year old" OR "four year old" OR "five year old" OR "six year old" OR "seven year old" OR "eight year old" OR "nine year old" OR "ten year old" OR "eleven year old" OR "twelve year old" OR "thirteen year old" OR "fourteen year old" OR "fifteen year old" OR "sixteen year old" OR "seventeen year old" OR "eighteen year old" OR "1 year old" OR "2 year old" OR "3 year old" OR "4 year old" OR "5 year old" OR "6 year old" OR "7 year old" OR "8 year old" OR "9 year old" OR "10 year old" OR "11 year old" OR "12 year old" OR "13 year old" OR "14 year old" OR "15 year old" OR "16 year old" OR "17 year old" OR "18 year old" OR "two years old" OR "three years old" OR "four years old" OR "five years old" OR "six years old" OR "seven years old" OR "eight years old" OR "nine years old" OR "ten years old" OR "eleven years old" OR "twelve years old" OR "thirteen years old" OR "fourteen years old" OR "fifteen years old" OR "sixteen years old" OR "seventeen years old" OR "eighteen years old" OR "2 years old" OR "3 years old" OR "4 years old" OR "5 years old" OR "6 years old" OR "7 years old" OR "8 years old" OR "9 years old" OR "10 years old" OR "11 years old" OR "12 years old" OR "13 years old" OR "14 years old" OR "15 years old" OR "16 years old" OR "17 years old" OR "18 years old"):ti) AND (("Injury" AND "Musculoskeletal System") OR "orthopedic trauma" OR "orthopedic traumas" OR "orthopaedic trauma" OR "orthopaedic traumas" OR "orthopedic injury" OR "orthopedic injuries" OR "orthopaedic injury" OR "orthopaedic injuries" OR "orthopedic surgery" OR "orthopedic surgeries" OR "orthopaedic surgery" OR "orthopaedic surgeries" OR "orthopedic intervention" OR "orthopedic interventions" OR "orthopaedic intervention" OR "orthopaedic interventions" OR "orthopedic trauma" OR "orthopedic procedures" OR "orthopaedic procedure" OR "orthopaedic procedures" OR "Arm Injury" OR "Back Injury" OR "Fracture" OR "Hand Injury" OR "Hip Injury" OR "Joint Dislocations" OR "Leg Injury" OR "Neck Injury" OR "Shoulder Injury" OR "Sprain" OR "Tendon Injury" OR "Ankle Fractures" OR "Ankle Injuries" OR "Arm Injuries" OR "Back Injuries" OR "Bone Diastasis" OR "Elbow Fractures" OR "Elbow Injuries" OR "Femoral Fractures" OR "Fibula Fractures" OR "Finger Injuries" OR "Foot Injuries" OR "Forearm Injuries" OR "Fracture" OR "Fracture Dislocation" OR "Fractures" OR "Hand Injuries" OR "Hip Dislocation" OR "Hip Fractures" OR "Hip Injuries" OR "Humeral Fractures" OR "Intra-Articular Fractures" OR "Joint Dislocations" OR "Knee Dislocation" OR "Knee Fractures" OR "Knee Injuries" OR "Leg Injuries" OR "Medial Tibial Stress Syndrome" OR "Muscle Diastasis" OR "Neck Injuries" OR "Osteoporotic Fractures" OR "Patellar Dislocation" OR "Periprosthetic Fractures" OR "Radius Fractures" OR "Rib Fractures" OR "Rotator Cuff Injuries" OR "Shoulder Dislocation" OR "Shoulder Fractures" OR "Shoulder Impingement Syndrome" OR "Shoulder Injuries" OR "Skull Fractures" OR "Spinal Fractures" OR "Spinal Injuries" OR "Sprain" OR "Tendinopathy" OR "Tendon Injuries" OR "Tibial Fractures" OR "Tibial Meniscus Injuries" OR "Traumatic Amputation" OR "Traumatic Multiple Amputations" OR "Ulna Fractures" OR "Whiplash Injuries" OR "Wrist Fractures" OR "Wrist Injuries" OR "Ankle Fracture" OR "Ankle Injury" OR "Arm Injury" OR "Back Injury" OR "Elbow Fracture" OR "Elbow Injury" OR "Femoral Fracture" OR "Fibula Fracture" OR "Finger Injury" OR "Foot Injury" OR "Forearm Injury" OR "Fracture Dislocations" OR "Hand Injury" OR "Hip Dislocations" OR "Hip Fracture" OR "Hip Injury" OR "Humeral Fracture" OR "Humeral Fracture" OR "Intra-Articular Fracture" OR "Joint Dislocation" OR "Knee Dislocations" OR "Knee Fracture" OR "Knee Injury" OR "Leg Injury" OR "Neck Injury" OR "Osteoporotic Fracture" OR "Patellar Dislocations" OR "Periprosthetic Fracture" OR "Radius Fracture" OR "Rib Fracture" OR "Rotator Cuff Injury" OR "Shoulder Dislocations" OR "Shoulder Fracture" OR "Shoulder Impingement" OR "Shoulder Injury" OR "Skull Fracture" OR "Spinal Fracture" OR "Spinal Injury" OR "Sprains" OR "Tendinopathies" OR "Tendon Injury" OR "Tibial Fracture" OR "Tibial Meniscus Injury" OR "Traumatic Amputations" OR "Traumatic Multiple Amputation" OR "Ulna Fracture" OR "Whiplash Injury" OR "Wrist Fracture" OR "Wrist Injury" OR "Orthopedics" OR "Orthopedic Surgery" OR "Acetabuloplast*" OR "Acetabuloplasty" OR "Alveolar Bone Graft*" OR "Alveolar Bone Grafting" OR "Ankle Replacement" OR "Anterior Cruciate Ligament Reconstruction" OR "Arthrodesis" OR "Arthroplast*" OR "Arthroplasty" OR "Arthroscop*" OR "Arthroscopy" OR "Bone Lengthening" OR "Bone Transplant*" OR "Bone Transplantation" OR "Bone-Patellar Tendon-Bone Graft*" OR "Bone-Patellar Tendon-Bone Grafting" OR "Cementoplast*" OR "Cementoplasty" OR "Disarticulation" OR "Diskectom*" OR "Diskectomy" OR "Distraction Osteogenesis" OR "Elbow Replacement" OR "Finger Replacement" OR "Fracture Fixation" OR "Genioplast*" OR "Genioplasty" OR "Hemiarthroplast*" OR "Hemiarthroplasty" OR "Hemipelvectom*" OR "Hemipelvectomy" OR "Hip Replacement" OR "Ilizarov Technique" OR "Joint Capsule Release" OR "Knee Replacement" OR "Kyphoplast*" OR "Kyphoplasty" OR "Laminectom*" OR "Laminectomy" OR "Le Fort Osteotom*" OR "Le Fort Osteotomy" OR "Limb Salvage" OR "Mandibular Osteotom*" OR "Mandibular Osteotomy" OR "Mandibular Reconstruction" OR "Maxillary Osteotom*" OR "Maxillary Osteotomy" OR "Meniscectom*" OR "Meniscectomy" OR "Open Fracture Reduction" OR "Orthognathic Surgical Procedure" OR "Orthognathic Surgical Procedures" OR "Osteotom*" OR "Osteotomy" OR "Posterior Cruciate Ligament Reconstruction" OR "Replacement Arthroplast*" OR "Replacement Arthroplasty" OR "Sagittal Split Ramus Osteotom*" OR "Sagittal Split Ramus Osteotomy" OR "Shoulder Replacement" OR "Sinus Floor Augmentation" OR "Spinal Fusion" OR "Surgical Amputation" OR "Synovectom*" OR "Synovectomy" OR "Tendon Transfer" OR "Tenodesis" OR "Tenotom*" OR "Tenotomy" OR "Total Disc Replacement" OR "Traction" OR "Ulnar Collateral Ligament Reconstruction" OR "Vertebroplast*" OR "Vertebroplasty" OR "Orthopedic Equipment" OR "orthopedic" OR "orthopedics" OR "orthopedic*" OR "orthopaedic" OR "orthopaedics" OR "orthopaedic*"):ti,ab,kw)

NOT (conference abstract OR meeting abstract OR conference proceeding OR conference proceedings):pt

**Embase**

((exp *"posttraumatic stress disorder"/ OR "Moral Injuries".ti,ab OR "Moral Injury".ti,ab OR "Post Traumatic Neuroses".ti,ab OR "Post Traumatic Neurosis".ti,ab OR "Post Traumatic Stress".ti,ab OR "Post Traumatic Stress Disorder".ti,ab OR "Post Traumatic Stress Disorders".ti,ab OR "Posttraumatic Neuroses".ti,ab OR "Posttraumatic Neurosis".ti,ab OR "Posttraumatic Stress".ti,ab OR "Posttraumatic Stress Disorder".ti,ab OR "Posttraumatic Stress Disorders".ti,ab OR (("PTSD" ADJ10 "stress") OR ("PTSS" ADJ10 "stress")).ti,ab OR (("Moral" ADJ6 "Injuries") OR ("Moral" ADJ6 "Injury") OR ("Post" ADJ6 "Traumatic" ADJ6 "Neuroses") OR ("Post" ADJ6 "Traumatic" ADJ6 "Neurosis") OR ("Post" ADJ6 "Traumatic" ADJ6 "Stress") OR ("Post" ADJ6 "Traumatic" ADJ6 "Stress" ADJ6 "Disorder") OR ("Post" ADJ6 "Traumatic" ADJ6 "Stress" ADJ6 "Disorders") OR ("Posttraumatic" ADJ6 "Neuroses") OR ("Posttraumatic" ADJ6 "Neurosis") OR ("Posttraumatic" ADJ6 "Stress") OR ("Posttraumatic" ADJ6 "Stress" ADJ6 "Disorder") OR ("Posttraumatic" ADJ6 "Stress" ADJ6 "Disorders")).ti,ab) AND (exp "Child"/ OR "child".ti,ab OR "children".ti,ab OR exp "Infant"/ OR "infant".ti,ab OR "infants".ti,ab OR "infancy".ti,ab OR "newborn".ti,ab OR "newborns".ti,ab OR "new-born".ti,ab OR "new-borns".ti,ab OR "neonate".ti,ab OR "neonates".ti,ab OR "neonatal".ti,ab OR "neo-nate".ti,ab OR "neo-nates".ti,ab OR "neo-natal".ti,ab OR "neonatology".ti,ab OR "NICU".ti OR "premature".ti,ab OR "prematures".ti,ab OR "pre-mature".ti,ab OR "pre-matures".ti,ab OR "preterm".ti,ab OR "pre-term".ti,ab OR "postnatal".ti,ab OR "post-natal".ti,ab OR "baby".ti,ab OR "babies".ti,ab OR "suckling".ti,ab OR "sucklings".ti,ab OR "toddler".ti,ab OR "toddlers".ti,ab OR "childhood".ti,ab OR "schoolchild".ti,ab OR "schoolchildren".ti,ab OR "childcare".ti,ab OR "child-care".ti,ab OR "youngster".ti,ab OR "youngsters".ti,ab OR "preschool".ti,ab OR "pre-school".ti,ab OR "kid".ti,ab OR "kids".ti,ab OR "boy".ti,ab OR "boys".ti,ab OR "girl".ti,ab OR "girls".ti,ab OR exp "Adolescent"/ OR "adolescent".ti,ab OR "adolescents".ti,ab OR "adolescence".ti,ab OR "pre-adolescent".ti,ab OR "pre-adolescents".ti,ab OR "pre-adolescence".ti,ab OR "schoolage".ti,ab OR "schoolboy".ti,ab OR "schoolboys".ti,ab OR "schoolgirl".ti,ab OR "schoolgirls".ti,ab OR "pre-puber".ti,ab OR "pre-puberty".ti,ab OR "prepuber".ti,ab OR "prepubers".ti,ab OR "prepuberty".ti,ab OR "puber".ti,ab OR "puberty".ti,ab OR "puberal".ti,ab OR "teenager".ti,ab OR "teenagers".ti,ab OR "teens".ti,ab OR "youth".ti,ab OR "youths".ti,ab OR "underaged".ti,ab OR "under-aged".ti,ab OR exp "Pediatrics"/ OR "Pediatric".ti,ab OR "Pediatrics".ti,ab OR "Paediatric".ti,ab OR "Paediatrics".ti,ab OR children*.ti,ab OR schoolchild*.ti,ab OR "infant".ti,ab OR "infants".ti,ab OR "infancy".ti,ab OR adolesc*.ti,ab OR pediat*.ti,ab OR paediat*.ti,ab OR neonat*.ti,ab OR toddler*.ti,ab OR "teen".ti,ab OR "teens".ti,ab OR teenager*.ti,ab OR preteen*.ti,ab OR newborn*.ti,ab OR postneonat*.ti,ab OR postnatal*.ti,ab OR "puberty".ti,ab OR preschool*.ti,ab OR suckling*.ti,ab OR "juvenile".ti,ab OR "new born".ti,ab OR "new borns".ti,ab OR new-born*.ti,ab OR neo-nat*.ti,ab OR neonat*.ti,ab OR perinat*.ti,ab OR underag*.ti,ab OR "under age".ti,ab OR "under aged".ti,ab OR youth*.ti,ab OR kinder*.ti,ab OR pubescen*.ti,ab OR prepubescen*.ti,ab OR "prepuberty".ti,ab OR "school age".ti,ab OR "schoolage".ti,ab OR "school ages".ti,ab OR schoolage*.ti,ab OR "one year old".ti OR "two year old".ti OR "three year old".ti OR "four year old".ti OR "five year old".ti OR "six year old".ti OR "seven year old".ti OR "eight year old".ti OR "nine year old".ti OR "ten year old".ti OR "eleven year old".ti OR "twelve year old".ti OR "thirteen year old".ti OR "fourteen year old".ti OR "fifteen year old".ti OR "sixteen year old".ti OR "seventeen year old".ti OR "eighteen year old".ti OR "1 year old".ti OR "2 year old".ti OR "3 year old".ti OR "4 year old".ti OR "5 year old".ti OR "6 year old".ti OR "7 year old".ti OR "8 year old".ti OR "9 year old".ti OR "10 year old".ti OR "11 year old".ti OR "12 year old".ti OR "13 year old".ti OR "14 year old".ti OR "15 year old".ti OR "16 year old".ti OR "17 year old".ti OR "18 year old".ti OR "two years old".ti OR "three years old".ti OR "four years old".ti OR "five years old".ti OR "six years old".ti OR "seven years old".ti OR "eight years old".ti OR "nine years old".ti OR "ten years old".ti OR "eleven years old".ti OR "twelve years old".ti OR "thirteen years old".ti OR "fourteen years old".ti OR "fifteen years old".ti OR "sixteen years old".ti OR "seventeen years old".ti OR "eighteen years old".ti OR "2 years old".ti OR "3 years old".ti OR "4 years old".ti OR "5 years old".ti OR "6 years old".ti OR "7 years old".ti OR "8 years old".ti OR "9 years old".ti OR "10 years old".ti OR "11 years old".ti OR "12 years old".ti OR "13 years old".ti OR "14 years old".ti OR "15 years old".ti OR "16 years old".ti OR "17 years old".ti OR "18 years old".ti) AND ((*"Injury"/ AND exp *"Musculoskeletal System"/) OR "orthopedic trauma".ti,ab OR "orthopedic traumas".ti,ab OR "orthopaedic trauma".ti,ab OR "orthopaedic traumas".ti,ab OR "orthopedic injury".ti,ab OR "orthopedic injuries".ti,ab OR "orthopaedic injury".ti,ab OR "orthopaedic injuries".ti,ab OR "orthopedic surgery".ti,ab OR "orthopedic surgeries".ti,ab OR "orthopaedic surgery".ti,ab OR "orthopaedic surgeries".ti,ab OR "orthopedic intervention".ti,ab OR "orthopedic interventions".ti,ab OR "orthopaedic intervention".ti,ab OR "orthopaedic interventions".ti,ab OR "orthopedic trauma".ti,ab OR "orthopedic procedures".ti,ab OR "orthopaedic procedure".ti,ab OR "orthopaedic procedures".ti,ab OR exp *"Fracture"/ OR "Ankle Fractures".ti,ab OR "Ankle Injuries".ti,ab OR "Arm Injuries".ti,ab OR "Back Injuries".ti,ab OR "Bone Diastasis".ti,ab OR "Elbow Fractures".ti,ab OR "Elbow Injuries".ti,ab OR "Femoral Fractures".ti,ab OR "Fibula Fractures".ti,ab OR "Finger Injuries".ti,ab OR "Foot Injuries".ti,ab OR "Forearm Injuries".ti,ab OR "Fracture".ti,ab OR "Fracture Dislocation".ti,ab OR "Fractures".ti,ab OR "Hand Injuries".ti,ab OR "Hip Dislocation".ti,ab OR "Hip Fractures".ti,ab OR "Hip Injuries".ti,ab OR "Humeral Fractures".ti,ab OR "Intra-Articular Fractures".ti,ab OR "Joint Dislocations".ti,ab OR "Knee Dislocation".ti,ab OR "Knee Fractures".ti,ab OR "Knee Injuries".ti,ab OR "Leg Injuries".ti,ab OR "Medial Tibial Stress Syndrome".ti,ab OR "Muscle Diastasis".ti,ab OR "Neck Injuries".ti,ab OR "Osteoporotic Fractures".ti,ab OR "Patellar Dislocation".ti,ab OR "Periprosthetic Fractures".ti,ab OR "Radius Fractures".ti,ab OR "Rib Fractures".ti,ab OR "Rotator Cuff Injuries".ti,ab OR "Shoulder Dislocation".ti,ab OR "Shoulder Fractures".ti,ab OR "Shoulder Impingement Syndrome".ti,ab OR "Shoulder Injuries".ti,ab OR "Skull Fractures".ti,ab OR "Spinal Fractures".ti,ab OR "Spinal Injuries".ti,ab OR "Sprain".ti,ab OR "Tendinopathy".ti,ab OR "Tendon Injuries".ti,ab OR "Tibial Fractures".ti,ab OR "Tibial Meniscus Injuries".ti,ab OR "Traumatic Amputation".ti,ab OR "Traumatic Multiple Amputations".ti,ab OR "Ulna Fractures".ti,ab OR "Whiplash Injuries".ti,ab OR "Wrist Fractures".ti,ab OR "Wrist Injuries".ti,ab OR "Ankle Fracture".ti,ab OR "Ankle Injury".ti,ab OR "Arm Injury".ti,ab OR "Back Injury".ti,ab OR "Elbow Fracture".ti,ab OR "Elbow Injury".ti,ab OR "Femoral Fracture".ti,ab OR "Fibula Fracture".ti,ab OR "Finger Injury".ti,ab OR "Foot Injury".ti,ab OR "Forearm Injury".ti,ab OR "Fracture Dislocations".ti,ab OR "Hand Injury".ti,ab OR "Hip Dislocations".ti,ab OR "Hip Fracture".ti,ab OR "Hip Injury".ti,ab OR "Humeral Fracture".ti,ab OR "Humeral Fracture".ti,ab OR "Intra-Articular Fracture".ti,ab OR "Joint Dislocation".ti,ab OR "Knee Dislocations".ti,ab OR "Knee Fracture".ti,ab OR "Knee Injury".ti,ab OR "Leg Injury".ti,ab OR "Neck Injury".ti,ab OR "Osteoporotic Fracture".ti,ab OR "Patellar Dislocations".ti,ab OR "Periprosthetic Fracture".ti,ab OR "Radius Fracture".ti,ab OR "Rib Fracture".ti,ab OR "Rotator Cuff Injury".ti,ab OR "Shoulder Dislocations".ti,ab OR "Shoulder Fracture".ti,ab OR "Shoulder Impingement".ti,ab OR "Shoulder Injury".ti,ab OR "Skull Fracture".ti,ab OR "Spinal Fracture".ti,ab OR "Spinal Injury".ti,ab OR "Sprains".ti,ab OR "Tendinopathies".ti,ab OR "Tendon Injury".ti,ab OR "Tibial Fracture".ti,ab OR "Tibial Meniscus Injury".ti,ab OR "Traumatic Amputations".ti,ab OR "Traumatic Multiple Amputation".ti,ab OR "Ulna Fracture".ti,ab OR "Whiplash Injury".ti,ab OR "Wrist Fracture".ti,ab OR "Wrist Injury".ti,ab OR exp *"Orthopedics"/ OR exp *"Orthopedic Surgery"/ OR "Acetabuloplast*".ti,ab OR "Acetabuloplasty".ti,ab OR "Alveolar Bone Graft*".ti,ab OR "Alveolar Bone Grafting".ti,ab OR "Ankle Replacement".ti,ab OR "Anterior Cruciate Ligament Reconstruction".ti,ab OR "Arthrodesis".ti,ab OR "Arthroplast*".ti,ab OR "Arthroplasty".ti,ab OR "Arthroscop*".ti,ab OR "Arthroscopy".ti,ab OR "Bone Lengthening".ti,ab OR "Bone Transplant*".ti,ab OR "Bone Transplantation".ti,ab OR "Bone-Patellar Tendon-Bone Graft*".ti,ab OR "Bone-Patellar Tendon-Bone Grafting".ti,ab OR "Cementoplast*".ti,ab OR "Cementoplasty".ti,ab OR "Disarticulation".ti,ab OR "Diskectom*".ti,ab OR "Diskectomy".ti,ab OR "Distraction Osteogenesis".ti,ab OR "Elbow Replacement".ti,ab OR "Finger Replacement".ti,ab OR "Fracture Fixation".ti,ab OR "Genioplast*".ti,ab OR "Genioplasty".ti,ab OR "Hemiarthroplast*".ti,ab OR "Hemiarthroplasty".ti,ab OR "Hemipelvectom*".ti,ab OR "Hemipelvectomy".ti,ab OR "Hip Replacement".ti,ab OR "Ilizarov Technique".ti,ab OR "Joint Capsule Release".ti,ab OR "Knee Replacement".ti,ab OR "Kyphoplast*".ti,ab OR "Kyphoplasty".ti,ab OR "Laminectom*".ti,ab OR "Laminectomy".ti,ab OR "Le Fort Osteotom*".ti,ab OR "Le Fort Osteotomy".ti,ab OR "Limb Salvage".ti,ab OR "Mandibular Osteotom*".ti,ab OR "Mandibular Osteotomy".ti,ab OR "Mandibular Reconstruction".ti,ab OR "Maxillary Osteotom*".ti,ab OR "Maxillary Osteotomy".ti,ab OR "Meniscectom*".ti,ab OR "Meniscectomy".ti,ab OR "Open Fracture Reduction".ti,ab OR "Orthognathic Surgical Procedure".ti,ab OR "Orthognathic Surgical Procedures".ti,ab OR "Osteotom*".ti,ab OR "Osteotomy".ti,ab OR "Posterior Cruciate Ligament Reconstruction".ti,ab OR "Replacement Arthroplast*".ti,ab OR "Replacement Arthroplasty".ti,ab OR "Sagittal Split Ramus Osteotom*".ti,ab OR "Sagittal Split Ramus Osteotomy".ti,ab OR "Shoulder Replacement".ti,ab OR "Sinus Floor Augmentation".ti,ab OR "Spinal Fusion".ti,ab OR "Surgical Amputation".ti,ab OR "Synovectom*".ti,ab OR "Synovectomy".ti,ab OR "Tendon Transfer".ti,ab OR "Tenodesis".ti,ab OR "Tenotom*".ti,ab OR "Tenotomy".ti,ab OR "Total Disc Replacement".ti,ab OR "Traction".ti,ab OR "Ulnar Collateral Ligament Reconstruction".ti,ab OR "Vertebroplast*".ti,ab OR "Vertebroplasty".ti,ab OR exp *"Orthopedic Equipment"/ OR "orthopedic".ti,ab OR "orthopedics".ti,ab OR "orthopedic*".ti,ab OR "orthopaedic".ti,ab OR "orthopaedics".ti,ab OR "orthopaedic*".ti,ab)) NOT (conference review or conference abstract).pt

**Emcare**

((exp *"posttraumatic stress disorder"/ OR "Moral Injuries".ti,ab OR "Moral Injury".ti,ab OR "Post Traumatic Neuroses".ti,ab OR "Post Traumatic Neurosis".ti,ab OR "Post Traumatic Stress".ti,ab OR "Post Traumatic Stress Disorder".ti,ab OR "Post Traumatic Stress Disorders".ti,ab OR "Posttraumatic Neuroses".ti,ab OR "Posttraumatic Neurosis".ti,ab OR "Posttraumatic Stress".ti,ab OR "Posttraumatic Stress Disorder".ti,ab OR "Posttraumatic Stress Disorders".ti,ab OR (("PTSD" ADJ10 "stress") OR ("PTSS" ADJ10 "stress")).ti,ab OR (("Moral" ADJ6 "Injuries") OR ("Moral" ADJ6 "Injury") OR ("Post" ADJ6 "Traumatic" ADJ6 "Neuroses") OR ("Post" ADJ6 "Traumatic" ADJ6 "Neurosis") OR ("Post" ADJ6 "Traumatic" ADJ6 "Stress") OR ("Post" ADJ6 "Traumatic" ADJ6 "Stress" ADJ6 "Disorder") OR ("Post" ADJ6 "Traumatic" ADJ6 "Stress" ADJ6 "Disorders") OR ("Posttraumatic" ADJ6 "Neuroses") OR ("Posttraumatic" ADJ6 "Neurosis") OR ("Posttraumatic" ADJ6 "Stress") OR ("Posttraumatic" ADJ6 "Stress" ADJ6 "Disorder") OR ("Posttraumatic" ADJ6 "Stress" ADJ6 "Disorders")).ti,ab) AND (exp "Child"/ OR "child".ti,ab OR "children".ti,ab OR exp "Infant"/ OR "infant".ti,ab OR "infants".ti,ab OR "infancy".ti,ab OR "newborn".ti,ab OR "newborns".ti,ab OR "new-born".ti,ab OR "new-borns".ti,ab OR "neonate".ti,ab OR "neonates".ti,ab OR "neonatal".ti,ab OR "neo-nate".ti,ab OR "neo-nates".ti,ab OR "neo-natal".ti,ab OR "neonatology".ti,ab OR "NICU".ti OR "premature".ti,ab OR "prematures".ti,ab OR "pre-mature".ti,ab OR "pre-matures".ti,ab OR "preterm".ti,ab OR "pre-term".ti,ab OR "postnatal".ti,ab OR "post-natal".ti,ab OR "baby".ti,ab OR "babies".ti,ab OR "suckling".ti,ab OR "sucklings".ti,ab OR "toddler".ti,ab OR "toddlers".ti,ab OR "childhood".ti,ab OR "schoolchild".ti,ab OR "schoolchildren".ti,ab OR "childcare".ti,ab OR "child-care".ti,ab OR "youngster".ti,ab OR "youngsters".ti,ab OR "preschool".ti,ab OR "pre-school".ti,ab OR "kid".ti,ab OR "kids".ti,ab OR "boy".ti,ab OR "boys".ti,ab OR "girl".ti,ab OR "girls".ti,ab OR exp "Adolescent"/ OR "adolescent".ti,ab OR "adolescents".ti,ab OR "adolescence".ti,ab OR "pre-adolescent".ti,ab OR "pre-adolescents".ti,ab OR "pre-adolescence".ti,ab OR "schoolage".ti,ab OR "schoolboy".ti,ab OR "schoolboys".ti,ab OR "schoolgirl".ti,ab OR "schoolgirls".ti,ab OR "pre-puber".ti,ab OR "pre-puberty".ti,ab OR "prepuber".ti,ab OR "prepubers".ti,ab OR "prepuberty".ti,ab OR "puber".ti,ab OR "puberty".ti,ab OR "puberal".ti,ab OR "teenager".ti,ab OR "teenagers".ti,ab OR "teens".ti,ab OR "youth".ti,ab OR "youths".ti,ab OR "underaged".ti,ab OR "under-aged".ti,ab OR exp "Pediatrics"/ OR "Pediatric".ti,ab OR "Pediatrics".ti,ab OR "Paediatric".ti,ab OR "Paediatrics".ti,ab OR children*.ti,ab OR schoolchild*.ti,ab OR "infant".ti,ab OR "infants".ti,ab OR "infancy".ti,ab OR adolesc*.ti,ab OR pediat*.ti,ab OR paediat*.ti,ab OR neonat*.ti,ab OR toddler*.ti,ab OR "teen".ti,ab OR "teens".ti,ab OR teenager*.ti,ab OR preteen*.ti,ab OR newborn*.ti,ab OR postneonat*.ti,ab OR postnatal*.ti,ab OR "puberty".ti,ab OR preschool*.ti,ab OR suckling*.ti,ab OR "juvenile".ti,ab OR "new born".ti,ab OR "new borns".ti,ab OR new-born*.ti,ab OR neo-nat*.ti,ab OR neonat*.ti,ab OR perinat*.ti,ab OR underag*.ti,ab OR "under age".ti,ab OR "under aged".ti,ab OR youth*.ti,ab OR kinder*.ti,ab OR pubescen*.ti,ab OR prepubescen*.ti,ab OR "prepuberty".ti,ab OR "school age".ti,ab OR "schoolage".ti,ab OR "school ages".ti,ab OR schoolage*.ti,ab OR "one year old".ti OR "two year old".ti OR "three year old".ti OR "four year old".ti OR "five year old".ti OR "six year old".ti OR "seven year old".ti OR "eight year old".ti OR "nine year old".ti OR "ten year old".ti OR "eleven year old".ti OR "twelve year old".ti OR "thirteen year old".ti OR "fourteen year old".ti OR "fifteen year old".ti OR "sixteen year old".ti OR "seventeen year old".ti OR "eighteen year old".ti OR "1 year old".ti OR "2 year old".ti OR "3 year old".ti OR "4 year old".ti OR "5 year old".ti OR "6 year old".ti OR "7 year old".ti OR "8 year old".ti OR "9 year old".ti OR "10 year old".ti OR "11 year old".ti OR "12 year old".ti OR "13 year old".ti OR "14 year old".ti OR "15 year old".ti OR "16 year old".ti OR "17 year old".ti OR "18 year old".ti OR "two years old".ti OR "three years old".ti OR "four years old".ti OR "five years old".ti OR "six years old".ti OR "seven years old".ti OR "eight years old".ti OR "nine years old".ti OR "ten years old".ti OR "eleven years old".ti OR "twelve years old".ti OR "thirteen years old".ti OR "fourteen years old".ti OR "fifteen years old".ti OR "sixteen years old".ti OR "seventeen years old".ti OR "eighteen years old".ti OR "2 years old".ti OR "3 years old".ti OR "4 years old".ti OR "5 years old".ti OR "6 years old".ti OR "7 years old".ti OR "8 years old".ti OR "9 years old".ti OR "10 years old".ti OR "11 years old".ti OR "12 years old".ti OR "13 years old".ti OR "14 years old".ti OR "15 years old".ti OR "16 years old".ti OR "17 years old".ti OR "18 years old".ti) AND ((exp *"Injury"/ AND exp *"Musculoskeletal System"/) OR "orthopedic trauma".ti,ab OR "orthopedic traumas".ti,ab OR "orthopaedic trauma".ti,ab OR "orthopaedic traumas".ti,ab OR "orthopedic injury".ti,ab OR "orthopedic injuries".ti,ab OR "orthopaedic injury".ti,ab OR "orthopaedic injuries".ti,ab OR "orthopedic surgery".ti,ab OR "orthopedic surgeries".ti,ab OR "orthopaedic surgery".ti,ab OR "orthopaedic surgeries".ti,ab OR "orthopedic intervention".ti,ab OR "orthopedic interventions".ti,ab OR "orthopaedic intervention".ti,ab OR "orthopaedic interventions".ti,ab OR "orthopedic trauma".ti,ab OR "orthopedic procedures".ti,ab OR "orthopaedic procedure".ti,ab OR "orthopaedic procedures".ti,ab OR exp *"Arm Injury"/ OR exp *"Back Injury"/ OR exp *"Fracture"/ OR exp *"Hand Injury"/ OR exp *"Hip Injury"/ OR exp *"Joint Dislocations"/ OR exp *"Leg Injury"/ OR exp *"Neck Injury"/ OR exp *"Shoulder Injury"/ OR exp *"Sprain"/ OR exp *"Tendon Injury"/ OR "Ankle Fractures".ti,ab OR "Ankle Injuries".ti,ab OR "Arm Injuries".ti,ab OR "Back Injuries".ti,ab OR "Bone Diastasis".ti,ab OR "Elbow Fractures".ti,ab OR "Elbow Injuries".ti,ab OR "Femoral Fractures".ti,ab OR "Fibula Fractures".ti,ab OR "Finger Injuries".ti,ab OR "Foot Injuries".ti,ab OR "Forearm Injuries".ti,ab OR "Fracture".ti,ab OR "Fracture Dislocation".ti,ab OR "Fractures".ti,ab OR "Hand Injuries".ti,ab OR "Hip Dislocation".ti,ab OR "Hip Fractures".ti,ab OR "Hip Injuries".ti,ab OR "Humeral Fractures".ti,ab OR "Intra-Articular Fractures".ti,ab OR "Joint Dislocations".ti,ab OR "Knee Dislocation".ti,ab OR "Knee Fractures".ti,ab OR "Knee Injuries".ti,ab OR "Leg Injuries".ti,ab OR "Medial Tibial Stress Syndrome".ti,ab OR "Muscle Diastasis".ti,ab OR "Neck Injuries".ti,ab OR "Osteoporotic Fractures".ti,ab OR "Patellar Dislocation".ti,ab OR "Periprosthetic Fractures".ti,ab OR "Radius Fractures".ti,ab OR "Rib Fractures".ti,ab OR "Rotator Cuff Injuries".ti,ab OR "Shoulder Dislocation".ti,ab OR "Shoulder Fractures".ti,ab OR "Shoulder Impingement Syndrome".ti,ab OR "Shoulder Injuries".ti,ab OR "Skull Fractures".ti,ab OR "Spinal Fractures".ti,ab OR "Spinal Injuries".ti,ab OR "Sprain".ti,ab OR "Tendinopathy".ti,ab OR "Tendon Injuries".ti,ab OR "Tibial Fractures".ti,ab OR "Tibial Meniscus Injuries".ti,ab OR "Traumatic Amputation".ti,ab OR "Traumatic Multiple Amputations".ti,ab OR "Ulna Fractures".ti,ab OR "Whiplash Injuries".ti,ab OR "Wrist Fractures".ti,ab OR "Wrist Injuries".ti,ab OR "Ankle Fracture".ti,ab OR "Ankle Injury".ti,ab OR "Arm Injury".ti,ab OR "Back Injury".ti,ab OR "Elbow Fracture".ti,ab OR "Elbow Injury".ti,ab OR "Femoral Fracture".ti,ab OR "Fibula Fracture".ti,ab OR "Finger Injury".ti,ab OR "Foot Injury".ti,ab OR "Forearm Injury".ti,ab OR "Fracture Dislocations".ti,ab OR "Hand Injury".ti,ab OR "Hip Dislocations".ti,ab OR "Hip Fracture".ti,ab OR "Hip Injury".ti,ab OR "Humeral Fracture".ti,ab OR "Humeral Fracture".ti,ab OR "Intra-Articular Fracture".ti,ab OR "Joint Dislocation".ti,ab OR "Knee Dislocations".ti,ab OR "Knee Fracture".ti,ab OR "Knee Injury".ti,ab OR "Leg Injury".ti,ab OR "Neck Injury".ti,ab OR "Osteoporotic Fracture".ti,ab OR "Patellar Dislocations".ti,ab OR "Periprosthetic Fracture".ti,ab OR "Radius Fracture".ti,ab OR "Rib Fracture".ti,ab OR "Rotator Cuff Injury".ti,ab OR "Shoulder Dislocations".ti,ab OR "Shoulder Fracture".ti,ab OR "Shoulder Impingement".ti,ab OR "Shoulder Injury".ti,ab OR "Skull Fracture".ti,ab OR "Spinal Fracture".ti,ab OR "Spinal Injury".ti,ab OR "Sprains".ti,ab OR "Tendinopathies".ti,ab OR "Tendon Injury".ti,ab OR "Tibial Fracture".ti,ab OR "Tibial Meniscus Injury".ti,ab OR "Traumatic Amputations".ti,ab OR "Traumatic Multiple Amputation".ti,ab OR "Ulna Fracture".ti,ab OR "Whiplash Injury".ti,ab OR "Wrist Fracture".ti,ab OR "Wrist Injury".ti,ab OR exp *"Orthopedics"/ OR exp *"Orthopedic Surgery"/ OR "Acetabuloplast*".ti,ab OR "Acetabuloplasty".ti,ab OR "Alveolar Bone Graft*".ti,ab OR "Alveolar Bone Grafting".ti,ab OR "Ankle Replacement".ti,ab OR "Anterior Cruciate Ligament Reconstruction".ti,ab OR "Arthrodesis".ti,ab OR "Arthroplast*".ti,ab OR "Arthroplasty".ti,ab OR "Arthroscop*".ti,ab OR "Arthroscopy".ti,ab OR "Bone Lengthening".ti,ab OR "Bone Transplant*".ti,ab OR "Bone Transplantation".ti,ab OR "Bone-Patellar Tendon-Bone Graft*".ti,ab OR "Bone-Patellar Tendon-Bone Grafting".ti,ab OR "Cementoplast*".ti,ab OR "Cementoplasty".ti,ab OR "Disarticulation".ti,ab OR "Diskectom*".ti,ab OR "Diskectomy".ti,ab OR "Distraction Osteogenesis".ti,ab OR "Elbow Replacement".ti,ab OR "Finger Replacement".ti,ab OR "Fracture Fixation".ti,ab OR "Genioplast*".ti,ab OR "Genioplasty".ti,ab OR "Hemiarthroplast*".ti,ab OR "Hemiarthroplasty".ti,ab OR "Hemipelvectom*".ti,ab OR "Hemipelvectomy".ti,ab OR "Hip Replacement".ti,ab OR "Ilizarov Technique".ti,ab OR "Joint Capsule Release".ti,ab OR "Knee Replacement".ti,ab OR "Kyphoplast*".ti,ab OR "Kyphoplasty".ti,ab OR "Laminectom*".ti,ab OR "Laminectomy".ti,ab OR "Le Fort Osteotom*".ti,ab OR "Le Fort Osteotomy".ti,ab OR "Limb Salvage".ti,ab OR "Mandibular Osteotom*".ti,ab OR "Mandibular Osteotomy".ti,ab OR "Mandibular Reconstruction".ti,ab OR "Maxillary Osteotom*".ti,ab OR "Maxillary Osteotomy".ti,ab OR "Meniscectom*".ti,ab OR "Meniscectomy".ti,ab OR "Open Fracture Reduction".ti,ab OR "Orthognathic Surgical Procedure".ti,ab OR "Orthognathic Surgical Procedures".ti,ab OR "Osteotom*".ti,ab OR "Osteotomy".ti,ab OR "Posterior Cruciate Ligament Reconstruction".ti,ab OR "Replacement Arthroplast*".ti,ab OR "Replacement Arthroplasty".ti,ab OR "Sagittal Split Ramus Osteotom*".ti,ab OR "Sagittal Split Ramus Osteotomy".ti,ab OR "Shoulder Replacement".ti,ab OR "Sinus Floor Augmentation".ti,ab OR "Spinal Fusion".ti,ab OR "Surgical Amputation".ti,ab OR "Synovectom*".ti,ab OR "Synovectomy".ti,ab OR "Tendon Transfer".ti,ab OR "Tenodesis".ti,ab OR "Tenotom*".ti,ab OR "Tenotomy".ti,ab OR "Total Disc Replacement".ti,ab OR "Traction".ti,ab OR "Ulnar Collateral Ligament Reconstruction".ti,ab OR "Vertebroplast*".ti,ab OR "Vertebroplasty".ti,ab OR exp *"Orthopedic Equipment"/ OR "orthopedic".ti,ab OR "orthopedics".ti,ab OR "orthopedic*".ti,ab OR "orthopaedic".ti,ab OR "orthopaedics".ti,ab OR "orthopaedic*".ti,ab))

**PsycINFO**

((TI("posttraumatic stress disorder" OR "Moral Injuries" OR "Moral Injury" OR "Post Traumatic Neuroses" OR "Post Traumatic Neurosis" OR "Post Traumatic Stress" OR "Post Traumatic Stress Disorder" OR "Post Traumatic Stress Disorders" OR "Posttraumatic Neuroses" OR "Posttraumatic Neurosis" OR "Posttraumatic Stress" OR "Posttraumatic Stress Disorder" OR "Posttraumatic Stress Disorders" OR (("PTSD" NEAR/10 "stress") OR ("PTSS" NEAR/10 "stress")) OR (("Moral" NEAR/6 "Injuries") OR ("Moral" NEAR/6 "Injury") OR ("Post" NEAR/6 "Traumatic" NEAR/6 "Neuroses") OR ("Post" NEAR/6 "Traumatic" NEAR/6 "Neurosis") OR ("Post" NEAR/6 "Traumatic" NEAR/6 "Stress") OR ("Post" NEAR/6 "Traumatic" NEAR/6 "Stress" NEAR/6 "Disorder") OR ("Post" NEAR/6 "Traumatic" NEAR/6 "Stress" NEAR/6 "Disorders") OR ("Posttraumatic" NEAR/6 "Neuroses") OR ("Posttraumatic" NEAR/6 "Neurosis") OR ("Posttraumatic" NEAR/6 "Stress") OR ("Posttraumatic" NEAR/6 "Stress" NEAR/6 "Disorder") OR ("Posttraumatic" NEAR/6 "Stress" NEAR/6 "Disorders"))) OR SU("posttraumatic stress disorder" OR "Moral Injuries" OR "Moral Injury" OR "Post Traumatic Neuroses" OR "Post Traumatic Neurosis" OR "Post Traumatic Stress" OR "Post Traumatic Stress Disorder" OR "Post Traumatic Stress Disorders" OR "Posttraumatic Neuroses" OR "Posttraumatic Neurosis" OR "Posttraumatic Stress" OR "Posttraumatic Stress Disorder" OR "Posttraumatic Stress Disorders" OR (("PTSD" NEAR/10 "stress") OR ("PTSS" NEAR/10 "stress")) OR (("Moral" NEAR/6 "Injuries") OR ("Moral" NEAR/6 "Injury") OR ("Post" NEAR/6 "Traumatic" NEAR/6 "Neuroses") OR ("Post" NEAR/6 "Traumatic" NEAR/6 "Neurosis") OR ("Post" NEAR/6 "Traumatic" NEAR/6 "Stress") OR ("Post" NEAR/6 "Traumatic" NEAR/6 "Stress" NEAR/6 "Disorder") OR ("Post" NEAR/6 "Traumatic" NEAR/6 "Stress" NEAR/6 "Disorders") OR ("Posttraumatic" NEAR/6 "Neuroses") OR ("Posttraumatic" NEAR/6 "Neurosis") OR ("Posttraumatic" NEAR/6 "Stress") OR ("Posttraumatic" NEAR/6 "Stress" NEAR/6 "Disorder") OR ("Posttraumatic" NEAR/6 "Stress" NEAR/6 "Disorders"))) OR DE("posttraumatic stress disorder" OR "Moral Injuries" OR "Moral Injury" OR "Post Traumatic Neuroses" OR "Post Traumatic Neurosis" OR "Post Traumatic Stress" OR "Post Traumatic Stress Disorder" OR "Post Traumatic Stress Disorders" OR "Posttraumatic Neuroses" OR "Posttraumatic Neurosis" OR "Posttraumatic Stress" OR "Posttraumatic Stress Disorder" OR "Posttraumatic Stress Disorders" OR (("PTSD" NEAR/10 "stress") OR ("PTSS" NEAR/10 "stress")) OR (("Moral" NEAR/6 "Injuries") OR ("Moral" NEAR/6 "Injury") OR ("Post" NEAR/6 "Traumatic" NEAR/6 "Neuroses") OR ("Post" NEAR/6 "Traumatic" NEAR/6 "Neurosis") OR ("Post" NEAR/6 "Traumatic" NEAR/6 "Stress") OR ("Post" NEAR/6 "Traumatic" NEAR/6 "Stress" NEAR/6 "Disorder") OR ("Post" NEAR/6 "Traumatic" NEAR/6 "Stress" NEAR/6 "Disorders") OR ("Posttraumatic" NEAR/6 "Neuroses") OR ("Posttraumatic" NEAR/6 "Neurosis") OR ("Posttraumatic" NEAR/6 "Stress") OR ("Posttraumatic" NEAR/6 "Stress" NEAR/6 "Disorder") OR ("Posttraumatic" NEAR/6 "Stress" NEAR/6 "Disorders"))) OR MJ("posttraumatic stress disorder" OR "Moral Injuries" OR "Moral Injury" OR "Post Traumatic Neuroses" OR "Post Traumatic Neurosis" OR "Post Traumatic Stress" OR "Post Traumatic Stress Disorder" OR "Post Traumatic Stress Disorders" OR "Posttraumatic Neuroses" OR "Posttraumatic Neurosis" OR "Posttraumatic Stress" OR "Posttraumatic Stress Disorder" OR "Posttraumatic Stress Disorders" OR (("PTSD" NEAR/10 "stress") OR ("PTSS" NEAR/10 "stress")) OR (("Moral" NEAR/6 "Injuries") OR ("Moral" NEAR/6 "Injury") OR ("Post" NEAR/6 "Traumatic" NEAR/6 "Neuroses") OR ("Post" NEAR/6 "Traumatic" NEAR/6 "Neurosis") OR ("Post" NEAR/6 "Traumatic" NEAR/6 "Stress") OR ("Post" NEAR/6 "Traumatic" NEAR/6 "Stress" NEAR/6 "Disorder") OR ("Post" NEAR/6 "Traumatic" NEAR/6 "Stress" NEAR/6 "Disorders") OR ("Posttraumatic" NEAR/6 "Neuroses") OR ("Posttraumatic" NEAR/6 "Neurosis") OR ("Posttraumatic" NEAR/6 "Stress") OR ("Posttraumatic" NEAR/6 "Stress" NEAR/6 "Disorder") OR ("Posttraumatic" NEAR/6 "Stress" NEAR/6 "Disorders"))) OR MA("posttraumatic stress disorder" OR "Moral Injuries" OR "Moral Injury" OR "Post Traumatic Neuroses" OR "Post Traumatic Neurosis" OR "Post Traumatic Stress" OR "Post Traumatic Stress Disorder" OR "Post Traumatic Stress Disorders" OR "Posttraumatic Neuroses" OR "Posttraumatic Neurosis" OR "Posttraumatic Stress" OR "Posttraumatic Stress Disorder" OR "Posttraumatic Stress Disorders" OR (("PTSD" NEAR/10 "stress") OR ("PTSS" NEAR/10 "stress")) OR (("Moral" NEAR/6 "Injuries") OR ("Moral" NEAR/6 "Injury") OR ("Post" NEAR/6 "Traumatic" NEAR/6 "Neuroses") OR ("Post" NEAR/6 "Traumatic" NEAR/6 "Neurosis") OR ("Post" NEAR/6 "Traumatic" NEAR/6 "Stress") OR ("Post" NEAR/6 "Traumatic" NEAR/6 "Stress" NEAR/6 "Disorder") OR ("Post" NEAR/6 "Traumatic" NEAR/6 "Stress" NEAR/6 "Disorders") OR ("Posttraumatic" NEAR/6 "Neuroses") OR ("Posttraumatic" NEAR/6 "Neurosis") OR ("Posttraumatic" NEAR/6 "Stress") OR ("Posttraumatic" NEAR/6 "Stress" NEAR/6 "Disorder") OR ("Posttraumatic" NEAR/6 "Stress" NEAR/6 "Disorders"))) OR AB("posttraumatic stress disorder" OR "Moral Injuries" OR "Moral Injury" OR "Post Traumatic Neuroses" OR "Post Traumatic Neurosis" OR "Post Traumatic Stress" OR "Post Traumatic Stress Disorder" OR "Post Traumatic Stress Disorders" OR "Posttraumatic Neuroses" OR "Posttraumatic Neurosis" OR "Posttraumatic Stress" OR "Posttraumatic Stress Disorder" OR "Posttraumatic Stress Disorders" OR (("PTSD" NEAR/10 "stress") OR ("PTSS" NEAR/10 "stress")) OR (("Moral" NEAR/6 "Injuries") OR ("Moral" NEAR/6 "Injury") OR ("Post" NEAR/6 "Traumatic" NEAR/6 "Neuroses") OR ("Post" NEAR/6 "Traumatic" NEAR/6 "Neurosis") OR ("Post" NEAR/6 "Traumatic" NEAR/6 "Stress") OR ("Post" NEAR/6 "Traumatic" NEAR/6 "Stress" NEAR/6 "Disorder") OR ("Post" NEAR/6 "Traumatic" NEAR/6 "Stress" NEAR/6 "Disorders") OR ("Posttraumatic" NEAR/6 "Neuroses") OR ("Posttraumatic" NEAR/6 "Neurosis") OR ("Posttraumatic" NEAR/6 "Stress") OR ("Posttraumatic" NEAR/6 "Stress" NEAR/6 "Disorder") OR ("Posttraumatic" NEAR/6 "Stress" NEAR/6 "Disorders")))) AND (TI("child" OR "children" OR exp "Infant" OR "infant" OR "infants" OR "infancy" OR "newborn" OR "newborns" OR "new-born" OR "new-borns" OR "neonate" OR "neonates" OR "neonatal" OR "neo-nate" OR "neo-nates" OR "neo-natal" OR "neonatology" OR "NICU" OR "premature" OR "prematures" OR "pre-mature" OR "pre-matures" OR "preterm" OR "pre-term" OR "postnatal" OR "post-natal" OR "baby" OR "babies" OR "suckling" OR "sucklings" OR "toddler" OR "toddlers" OR "childhood" OR "schoolchild" OR "schoolchildren" OR "childcare" OR "child-care" OR "youngster" OR "youngsters" OR "preschool" OR "pre-school" OR "kid" OR "kids" OR "boy" OR "boys" OR "girl" OR "girls" OR exp "Adolescent" OR "adolescent" OR "adolescents" OR "adolescence" OR "pre-adolescent" OR "pre-adolescents" OR "pre-adolescence" OR "schoolage" OR "schoolboy" OR "schoolboys" OR "schoolgirl" OR "schoolgirls" OR "pre-puber" OR "pre-puberty" OR "prepuber" OR "prepubers" OR "prepuberty" OR "puber" OR "puberty" OR "puberal" OR "teenager" OR "teenagers" OR "teens" OR "youth" OR "youths" OR "underaged" OR "under-aged" OR exp "Pediatrics" OR "Pediatric" OR "Pediatrics" OR "Paediatric" OR "Paediatrics" **OR** "child" OR children* OR schoolchild* OR "infant" OR "infants" OR "infancy" OR adolesc* OR pediat* OR paediat* OR neonat* OR toddler* OR "teen" OR "teens" OR teenager* OR preteen* OR newborn* OR postneonat* OR postnatal* OR "puberty" OR preschool* OR suckling* OR "juvenile" OR "new born" OR "new borns" OR new-born* OR neo-nat* OR neonat* OR perinat* OR underag* OR "under age" OR "under aged" OR youth* OR kinder* OR pubescen* OR prepubescen* OR "prepuberty" OR "school age" OR "schoolage" OR "school ages" OR schoolage*) OR SU("child" OR "children" OR exp "Infant" OR "infant" OR "infants" OR "infancy" OR "newborn" OR "newborns" OR "new-born" OR "new-borns" OR "neonate" OR "neonates" OR "neonatal" OR "neo-nate" OR "neo-nates" OR "neo-natal" OR "neonatology" OR "NICU" OR "premature" OR "prematures" OR "pre-mature" OR "pre-matures" OR "preterm" OR "pre-term" OR "postnatal" OR "post-natal" OR "baby" OR "babies" OR "suckling" OR "sucklings" OR "toddler" OR "toddlers" OR "childhood" OR "schoolchild" OR "schoolchildren" OR "childcare" OR "child-care" OR "youngster" OR "youngsters" OR "preschool" OR "pre-school" OR "kid" OR "kids" OR "boy" OR "boys" OR "girl" OR "girls" OR exp "Adolescent" OR "adolescent" OR "adolescents" OR "adolescence" OR "pre-adolescent" OR "pre-adolescents" OR "pre-adolescence" OR "schoolage" OR "schoolboy" OR "schoolboys" OR "schoolgirl" OR "schoolgirls" OR "pre-puber" OR "pre-puberty" OR "prepuber" OR "prepubers" OR "prepuberty" OR "puber" OR "puberty" OR "puberal" OR "teenager" OR "teenagers" OR "teens" OR "youth" OR "youths" OR "underaged" OR "under-aged" OR exp "Pediatrics" OR "Pediatric" OR "Pediatrics" OR "Paediatric" OR "Paediatrics" **OR** "child" OR children* OR schoolchild* OR "infant" OR "infants" OR "infancy" OR adolesc* OR pediat* OR paediat* OR neonat* OR toddler* OR "teen" OR "teens" OR teenager* OR preteen* OR newborn* OR postneonat* OR postnatal* OR "puberty" OR preschool* OR suckling* OR "juvenile" OR "new born" OR "new borns" OR new-born* OR neo-nat* OR neonat* OR perinat* OR underag* OR "under age" OR "under aged" OR youth* OR kinder* OR pubescen* OR prepubescen* OR "prepuberty" OR "school age" OR "schoolage" OR "school ages" OR schoolage*) OR DE("child" OR "children" OR exp "Infant" OR "infant" OR "infants" OR "infancy" OR "newborn" OR "newborns" OR "new-born" OR "new-borns" OR "neonate" OR "neonates" OR "neonatal" OR "neo-nate" OR "neo-nates" OR "neo-natal" OR "neonatology" OR "NICU" OR "premature" OR "prematures" OR "pre-mature" OR "pre-matures" OR "preterm" OR "pre-term" OR "postnatal" OR "post-natal" OR "baby" OR "babies" OR "suckling" OR "sucklings" OR "toddler" OR "toddlers" OR "childhood" OR "schoolchild" OR "schoolchildren" OR "childcare" OR "child-care" OR "youngster" OR "youngsters" OR "preschool" OR "pre-school" OR "kid" OR "kids" OR "boy" OR "boys" OR "girl" OR "girls" OR exp "Adolescent" OR "adolescent" OR "adolescents" OR "adolescence" OR "pre-adolescent" OR "pre-adolescents" OR "pre-adolescence" OR "schoolage" OR "schoolboy" OR "schoolboys" OR "schoolgirl" OR "schoolgirls" OR "pre-puber" OR "pre-puberty" OR "prepuber" OR "prepubers" OR "prepuberty" OR "puber" OR "puberty" OR "puberal" OR "teenager" OR "teenagers" OR "teens" OR "youth" OR "youths" OR "underaged" OR "under-aged" OR exp "Pediatrics" OR "Pediatric" OR "Pediatrics" OR "Paediatric" OR "Paediatrics" **OR** "child" OR children* OR schoolchild* OR "infant" OR "infants" OR "infancy" OR adolesc* OR pediat* OR paediat* OR neonat* OR toddler* OR "teen" OR "teens" OR teenager* OR preteen* OR newborn* OR postneonat* OR postnatal* OR "puberty" OR preschool* OR suckling* OR "juvenile" OR "new born" OR "new borns" OR new-born* OR neo-nat* OR neonat* OR perinat* OR underag* OR "under age" OR "under aged" OR youth* OR kinder* OR pubescen* OR prepubescen* OR "prepuberty" OR "school age" OR "schoolage" OR "school ages" OR schoolage*) OR MJ("child" OR "children" OR exp "Infant" OR "infant" OR "infants" OR "infancy" OR "newborn" OR "newborns" OR "new-born" OR "new-borns" OR "neonate" OR "neonates" OR "neonatal" OR "neo-nate" OR "neo-nates" OR "neo-natal" OR "neonatology" OR "NICU" OR "premature" OR "prematures" OR "pre-mature" OR "pre-matures" OR "preterm" OR "pre-term" OR "postnatal" OR "post-natal" OR "baby" OR "babies" OR "suckling" OR "sucklings" OR "toddler" OR "toddlers" OR "childhood" OR "schoolchild" OR "schoolchildren" OR "childcare" OR "child-care" OR "youngster" OR "youngsters" OR "preschool" OR "pre-school" OR "kid" OR "kids" OR "boy" OR "boys" OR "girl" OR "girls" OR exp "Adolescent" OR "adolescent" OR "adolescents" OR "adolescence" OR "pre-adolescent" OR "pre-adolescents" OR "pre-adolescence" OR "schoolage" OR "schoolboy" OR "schoolboys" OR "schoolgirl" OR "schoolgirls" OR "pre-puber" OR "pre-puberty" OR "prepuber" OR "prepubers" OR "prepuberty" OR "puber" OR "puberty" OR "puberal" OR "teenager" OR "teenagers" OR "teens" OR "youth" OR "youths" OR "underaged" OR "under-aged" OR exp "Pediatrics" OR "Pediatric" OR "Pediatrics" OR "Paediatric" OR "Paediatrics" **OR** "child" OR children* OR schoolchild* OR "infant" OR "infants" OR "infancy" OR adolesc* OR pediat* OR paediat* OR neonat* OR toddler* OR "teen" OR "teens" OR teenager* OR preteen* OR newborn* OR postneonat* OR postnatal* OR "puberty" OR preschool* OR suckling* OR "juvenile" OR "new born" OR "new borns" OR new-born* OR neo-nat* OR neonat* OR perinat* OR underag* OR "under age" OR "under aged" OR youth* OR kinder* OR pubescen* OR prepubescen* OR "prepuberty" OR "school age" OR "schoolage" OR "school ages" OR schoolage*) OR MA("child" OR "children" OR exp "Infant" OR "infant" OR "infants" OR "infancy" OR "newborn" OR "newborns" OR "new-born" OR "new-borns" OR "neonate" OR "neonates" OR "neonatal" OR "neo-nate" OR "neo-nates" OR "neo-natal" OR "neonatology" OR "NICU" OR "premature" OR "prematures" OR "pre-mature" OR "pre-matures" OR "preterm" OR "pre-term" OR "postnatal" OR "post-natal" OR "baby" OR "babies" OR "suckling" OR "sucklings" OR "toddler" OR "toddlers" OR "childhood" OR "schoolchild" OR "schoolchildren" OR "childcare" OR "child-care" OR "youngster" OR "youngsters" OR "preschool" OR "pre-school" OR "kid" OR "kids" OR "boy" OR "boys" OR "girl" OR "girls" OR exp "Adolescent" OR "adolescent" OR "adolescents" OR "adolescence" OR "pre-adolescent" OR "pre-adolescents" OR "pre-adolescence" OR "schoolage" OR "schoolboy" OR "schoolboys" OR "schoolgirl" OR "schoolgirls" OR "pre-puber" OR "pre-puberty" OR "prepuber" OR "prepubers" OR "prepuberty" OR "puber" OR "puberty" OR "puberal" OR "teenager" OR "teenagers" OR "teens" OR "youth" OR "youths" OR "underaged" OR "under-aged" OR exp "Pediatrics" OR "Pediatric" OR "Pediatrics" OR "Paediatric" OR "Paediatrics" **OR** "child" OR children* OR schoolchild* OR "infant" OR "infants" OR "infancy" OR adolesc* OR pediat* OR paediat* OR neonat* OR toddler* OR "teen" OR "teens" OR teenager* OR preteen* OR newborn* OR postneonat* OR postnatal* OR "puberty" OR preschool* OR suckling* OR "juvenile" OR "new born" OR "new borns" OR new-born* OR neo-nat* OR neonat* OR perinat* OR underag* OR "under age" OR "under aged" OR youth* OR kinder* OR pubescen* OR prepubescen* OR "prepuberty" OR "school age" OR "schoolage" OR "school ages" OR schoolage*) OR AB("child" OR "children" OR exp "Infant" OR "infant" OR "infants" OR "infancy" OR "newborn" OR "newborns" OR "new-born" OR "new-borns" OR "neonate" OR "neonates" OR "neonatal" OR "neo-nate" OR "neo-nates" OR "neo-natal" OR "neonatology" OR "NICU" OR "premature" OR "prematures" OR "pre-mature" OR "pre-matures" OR "preterm" OR "pre-term" OR "postnatal" OR "post-natal" OR "baby" OR "babies" OR "suckling" OR "sucklings" OR "toddler" OR "toddlers" OR "childhood" OR "schoolchild" OR "schoolchildren" OR "childcare" OR "child-care" OR "youngster" OR "youngsters" OR "preschool" OR "pre-school" OR "kid" OR "kids" OR "boy" OR "boys" OR "girl" OR "girls" OR exp "Adolescent" OR "adolescent" OR "adolescents" OR "adolescence" OR "pre-adolescent" OR "pre-adolescents" OR "pre-adolescence" OR "schoolage" OR "schoolboy" OR "schoolboys" OR "schoolgirl" OR "schoolgirls" OR "pre-puber" OR "pre-puberty" OR "prepuber" OR "prepubers" OR "prepuberty" OR "puber" OR "puberty" OR "puberal" OR "teenager" OR "teenagers" OR "teens" OR "youth" OR "youths" OR "underaged" OR "under-aged" OR exp "Pediatrics" OR "Pediatric" OR "Pediatrics" OR "Paediatric" OR "Paediatrics" **OR** "child" OR children* OR schoolchild* OR "infant" OR "infants" OR "infancy" OR adolesc* OR pediat* OR paediat* OR neonat* OR toddler* OR "teen" OR "teens" OR teenager* OR preteen* OR newborn* OR postneonat* OR postnatal* OR "puberty" OR preschool* OR suckling* OR "juvenile" OR "new born" OR "new borns" OR new-born* OR neo-nat* OR neonat* OR perinat* OR underag* OR "under age" OR "under aged" OR youth* OR kinder* OR pubescen* OR prepubescen* OR "prepuberty" OR "school age" OR "schoolage" OR "school ages" OR schoolage*) OR TI("one year old" OR "two year old" OR "three year old" OR "four year old" OR "five year old" OR "six year old" OR "seven year old" OR "eight year old" OR "nine year old" OR "ten year old" OR "eleven year old" OR "twelve year old" OR "thirteen year old" OR "fourteen year old" OR "fifteen year old" OR "sixteen year old" OR "seventeen year old" OR "eighteen year old" OR "1 year old" OR "2 year old" OR "3 year old" OR "4 year old" OR "5 year old" OR "6 year old" OR "7 year old" OR "8 year old" OR "9 year old" OR "10 year old" OR "11 year old" OR "12 year old" OR "13 year old" OR "14 year old" OR "15 year old" OR "16 year old" OR "17 year old" OR "18 year old" OR "two years old" OR "three years old" OR "four years old" OR "five years old" OR "six years old" OR "seven years old" OR "eight years old" OR "nine years old" OR "ten years old" OR "eleven years old" OR "twelve years old" OR "thirteen years old" OR "fourteen years old" OR "fifteen years old" OR "sixteen years old" OR "seventeen years old" OR "eighteen years old" OR "2 years old" OR "3 years old" OR "4 years old" OR "5 years old" OR "6 years old" OR "7 years old" OR "8 years old" OR "9 years old" OR "10 years old" OR "11 years old" OR "12 years old" OR "13 years old" OR "14 years old" OR "15 years old" OR "16 years old" OR "17 years old" OR "18 years old")) AND (TI("orthopedic trauma" OR "orthopedic traumas" OR "orthopaedic trauma" OR "orthopaedic traumas" OR "orthopedic injury" OR "orthopedic injuries" OR "orthopaedic injury" OR "orthopaedic injuries" OR "orthopedic surgery" OR "orthopedic surgeries" OR "orthopaedic surgery" OR "orthopaedic surgeries" OR "orthopedic intervention" OR "orthopedic interventions" OR "orthopaedic intervention" OR "orthopaedic interventions" OR "orthopedic trauma" OR "orthopedic procedures" OR "orthopaedic procedure" OR "orthopaedic procedures" OR "Arm Injury" OR "Back Injury" OR "Fracture" OR "Hand Injury" OR "Hip Injury" OR "Joint Dislocations" OR "Leg Injury" OR "Neck Injury" OR "Shoulder Injury" OR "Sprain" OR "Tendon Injury" OR "Ankle Fractures" OR "Ankle Injuries" OR "Arm Injuries" OR "Back Injuries" OR "Bone Diastasis" OR "Elbow Fractures" OR "Elbow Injuries" OR "Femoral Fractures" OR "Fibula Fractures" OR "Finger Injuries" OR "Foot Injuries" OR "Forearm Injuries" OR "Fracture" OR "Fracture Dislocation" OR "Fractures" OR "Hand Injuries" OR "Hip Dislocation" OR "Hip Fractures" OR "Hip Injuries" OR "Humeral Fractures" OR "Intra-Articular Fractures" OR "Joint Dislocations" OR "Knee Dislocation" OR "Knee Fractures" OR "Knee Injuries" OR "Leg Injuries" OR "Medial Tibial Stress Syndrome" OR "Muscle Diastasis" OR "Neck Injuries" OR "Osteoporotic Fractures" OR "Patellar Dislocation" OR "Periprosthetic Fractures" OR "Radius Fractures" OR "Rib Fractures" OR "Rotator Cuff Injuries" OR "Shoulder Dislocation" OR "Shoulder Fractures" OR "Shoulder Impingement Syndrome" OR "Shoulder Injuries" OR "Skull Fractures" OR "Spinal Fractures" OR "Spinal Injuries" OR "Sprain" OR "Tendinopathy" OR "Tendon Injuries" OR "Tibial Fractures" OR "Tibial Meniscus Injuries" OR "Traumatic Amputation" OR "Traumatic Multiple Amputations" OR "Ulna Fractures" OR "Whiplash Injuries" OR "Wrist Fractures" OR "Wrist Injuries" OR "Ankle Fracture" OR "Ankle Injury" OR "Arm Injury" OR "Back Injury" OR "Elbow Fracture" OR "Elbow Injury" OR "Femoral Fracture" OR "Fibula Fracture" OR "Finger Injury" OR "Foot Injury" OR "Forearm Injury" OR "Fracture Dislocations" OR "Hand Injury" OR "Hip Dislocations" OR "Hip Fracture" OR "Hip Injury" OR "Humeral Fracture" OR "Humeral Fracture" OR "Intra-Articular Fracture" OR "Joint Dislocation" OR "Knee Dislocations" OR "Knee Fracture" OR "Knee Injury" OR "Leg Injury" OR "Neck Injury" OR "Osteoporotic Fracture" OR "Patellar Dislocations" OR "Periprosthetic Fracture" OR "Radius Fracture" OR "Rib Fracture" OR "Rotator Cuff Injury" OR "Shoulder Dislocations" OR "Shoulder Fracture" OR "Shoulder Impingement" OR "Shoulder Injury" OR "Skull Fracture" OR "Spinal Fracture" OR "Spinal Injury" OR "Sprains" OR "Tendinopathies" OR "Tendon Injury" OR "Tibial Fracture" OR "Tibial Meniscus Injury" OR "Traumatic Amputations" OR "Traumatic Multiple Amputation" OR "Ulna Fracture" OR "Whiplash Injury" OR "Wrist Fracture" OR "Wrist Injury" OR "Orthopedics" OR "Orthopedic Surgery" OR "Acetabuloplast*" OR "Acetabuloplasty" OR "Alveolar Bone Graft*" OR "Alveolar Bone Grafting" OR "Ankle Replacement" OR "Anterior Cruciate Ligament Reconstruction" OR "Arthrodesis" OR "Arthroplast*" OR "Arthroplasty" OR "Arthroscop*" OR "Arthroscopy" OR "Bone Lengthening" OR "Bone Transplant*" OR "Bone Transplantation" OR "Bone-Patellar Tendon-Bone Graft*" OR "Bone-Patellar Tendon-Bone Grafting" OR "Cementoplast*" OR "Cementoplasty" OR "Disarticulation" OR "Diskectom*" OR "Diskectomy" OR "Distraction Osteogenesis" OR "Elbow Replacement" OR "Finger Replacement" OR "Fracture Fixation" OR "Genioplast*" OR "Genioplasty" OR "Hemiarthroplast*" OR "Hemiarthroplasty" OR "Hemipelvectom*" OR "Hemipelvectomy" OR "Hip Replacement" OR "Ilizarov Technique" OR "Joint Capsule Release" OR "Knee Replacement" OR "Kyphoplast*" OR "Kyphoplasty" OR "Laminectom*" OR "Laminectomy" OR "Le Fort Osteotom*" OR "Le Fort Osteotomy" OR "Limb Salvage" OR "Mandibular Osteotom*" OR "Mandibular Osteotomy" OR "Mandibular Reconstruction" OR "Maxillary Osteotom*" OR "Maxillary Osteotomy" OR "Meniscectom*" OR "Meniscectomy" OR "Open Fracture Reduction" OR "Orthognathic Surgical Procedure" OR "Orthognathic Surgical Procedures" OR "Osteotom*" OR "Osteotomy" OR "Posterior Cruciate Ligament Reconstruction" OR "Replacement Arthroplast*" OR "Replacement Arthroplasty" OR "Sagittal Split Ramus Osteotom*" OR "Sagittal Split Ramus Osteotomy" OR "Shoulder Replacement" OR "Sinus Floor Augmentation" OR "Spinal Fusion" OR "Surgical Amputation" OR "Synovectom*" OR "Synovectomy" OR "Tendon Transfer" OR "Tenodesis" OR "Tenotom*" OR "Tenotomy" OR "Total Disc Replacement" OR "Traction" OR "Ulnar Collateral Ligament Reconstruction" OR "Vertebroplast*" OR "Vertebroplasty" OR "Orthopedic Equipment" OR "orthopedic" OR "orthopedics" OR "orthopedic*" OR "orthopaedic" OR "orthopaedics" OR "orthopaedic*") OR SU("orthopedic trauma" OR "orthopedic traumas" OR "orthopaedic trauma" OR "orthopaedic traumas" OR "orthopedic injury" OR "orthopedic injuries" OR "orthopaedic injury" OR "orthopaedic injuries" OR "orthopedic surgery" OR "orthopedic surgeries" OR "orthopaedic surgery" OR "orthopaedic surgeries" OR "orthopedic intervention" OR "orthopedic interventions" OR "orthopaedic intervention" OR "orthopaedic interventions" OR "orthopedic trauma" OR "orthopedic procedures" OR "orthopaedic procedure" OR "orthopaedic procedures" OR "Arm Injury" OR "Back Injury" OR "Fracture" OR "Hand Injury" OR "Hip Injury" OR "Joint Dislocations" OR "Leg Injury" OR "Neck Injury" OR "Shoulder Injury" OR "Sprain" OR "Tendon Injury" OR "Ankle Fractures" OR "Ankle Injuries" OR "Arm Injuries" OR "Back Injuries" OR "Bone Diastasis" OR "Elbow Fractures" OR "Elbow Injuries" OR "Femoral Fractures" OR "Fibula Fractures" OR "Finger Injuries" OR "Foot Injuries" OR "Forearm Injuries" OR "Fracture" OR "Fracture Dislocation" OR "Fractures" OR "Hand Injuries" OR "Hip Dislocation" OR "Hip Fractures" OR "Hip Injuries" OR "Humeral Fractures" OR "Intra-Articular Fractures" OR "Joint Dislocations" OR "Knee Dislocation" OR "Knee Fractures" OR "Knee Injuries" OR "Leg Injuries" OR "Medial Tibial Stress Syndrome" OR "Muscle Diastasis" OR "Neck Injuries" OR "Osteoporotic Fractures" OR "Patellar Dislocation" OR "Periprosthetic Fractures" OR "Radius Fractures" OR "Rib Fractures" OR "Rotator Cuff Injuries" OR "Shoulder Dislocation" OR "Shoulder Fractures" OR "Shoulder Impingement Syndrome" OR "Shoulder Injuries" OR "Skull Fractures" OR "Spinal Fractures" OR "Spinal Injuries" OR "Sprain" OR "Tendinopathy" OR "Tendon Injuries" OR "Tibial Fractures" OR "Tibial Meniscus Injuries" OR "Traumatic Amputation" OR "Traumatic Multiple Amputations" OR "Ulna Fractures" OR "Whiplash Injuries" OR "Wrist Fractures" OR "Wrist Injuries" OR "Ankle Fracture" OR "Ankle Injury" OR "Arm Injury" OR "Back Injury" OR "Elbow Fracture" OR "Elbow Injury" OR "Femoral Fracture" OR "Fibula Fracture" OR "Finger Injury" OR "Foot Injury" OR "Forearm Injury" OR "Fracture Dislocations" OR "Hand Injury" OR "Hip Dislocations" OR "Hip Fracture" OR "Hip Injury" OR "Humeral Fracture" OR "Humeral Fracture" OR "Intra-Articular Fracture" OR "Joint Dislocation" OR "Knee Dislocations" OR "Knee Fracture" OR "Knee Injury" OR "Leg Injury" OR "Neck Injury" OR "Osteoporotic Fracture" OR "Patellar Dislocations" OR "Periprosthetic Fracture" OR "Radius Fracture" OR "Rib Fracture" OR "Rotator Cuff Injury" OR "Shoulder Dislocations" OR "Shoulder Fracture" OR "Shoulder Impingement" OR "Shoulder Injury" OR "Skull Fracture" OR "Spinal Fracture" OR "Spinal Injury" OR "Sprains" OR "Tendinopathies" OR "Tendon Injury" OR "Tibial Fracture" OR "Tibial Meniscus Injury" OR "Traumatic Amputations" OR "Traumatic Multiple Amputation" OR "Ulna Fracture" OR "Whiplash Injury" OR "Wrist Fracture" OR "Wrist Injury" OR "Orthopedics" OR "Orthopedic Surgery" OR "Acetabuloplast*" OR "Acetabuloplasty" OR "Alveolar Bone Graft*" OR "Alveolar Bone Grafting" OR "Ankle Replacement" OR "Anterior Cruciate Ligament Reconstruction" OR "Arthrodesis" OR "Arthroplast*" OR "Arthroplasty" OR "Arthroscop*" OR "Arthroscopy" OR "Bone Lengthening" OR "Bone Transplant*" OR "Bone Transplantation" OR "Bone-Patellar Tendon-Bone Graft*" OR "Bone-Patellar Tendon-Bone Grafting" OR "Cementoplast*" OR "Cementoplasty" OR "Disarticulation" OR "Diskectom*" OR "Diskectomy" OR "Distraction Osteogenesis" OR "Elbow Replacement" OR "Finger Replacement" OR "Fracture Fixation" OR "Genioplast*" OR "Genioplasty" OR "Hemiarthroplast*" OR "Hemiarthroplasty" OR "Hemipelvectom*" OR "Hemipelvectomy" OR "Hip Replacement" OR "Ilizarov Technique" OR "Joint Capsule Release" OR "Knee Replacement" OR "Kyphoplast*" OR "Kyphoplasty" OR "Laminectom*" OR "Laminectomy" OR "Le Fort Osteotom*" OR "Le Fort Osteotomy" OR "Limb Salvage" OR "Mandibular Osteotom*" OR "Mandibular Osteotomy" OR "Mandibular Reconstruction" OR "Maxillary Osteotom*" OR "Maxillary Osteotomy" OR "Meniscectom*" OR "Meniscectomy" OR "Open Fracture Reduction" OR "Orthognathic Surgical Procedure" OR "Orthognathic Surgical Procedures" OR "Osteotom*" OR "Osteotomy" OR "Posterior Cruciate Ligament Reconstruction" OR "Replacement Arthroplast*" OR "Replacement Arthroplasty" OR "Sagittal Split Ramus Osteotom*" OR "Sagittal Split Ramus Osteotomy" OR "Shoulder Replacement" OR "Sinus Floor Augmentation" OR "Spinal Fusion" OR "Surgical Amputation" OR "Synovectom*" OR "Synovectomy" OR "Tendon Transfer" OR "Tenodesis" OR "Tenotom*" OR "Tenotomy" OR "Total Disc Replacement" OR "Traction" OR "Ulnar Collateral Ligament Reconstruction" OR "Vertebroplast*" OR "Vertebroplasty" OR "Orthopedic Equipment" OR "orthopedic" OR "orthopedics" OR "orthopedic*" OR "orthopaedic" OR "orthopaedics" OR "orthopaedic*") OR DE("orthopedic trauma" OR "orthopedic traumas" OR "orthopaedic trauma" OR "orthopaedic traumas" OR "orthopedic injury" OR "orthopedic injuries" OR "orthopaedic injury" OR "orthopaedic injuries" OR "orthopedic surgery" OR "orthopedic surgeries" OR "orthopaedic surgery" OR "orthopaedic surgeries" OR "orthopedic intervention" OR "orthopedic interventions" OR "orthopaedic intervention" OR "orthopaedic interventions" OR "orthopedic trauma" OR "orthopedic procedures" OR "orthopaedic procedure" OR "orthopaedic procedures" OR "Arm Injury" OR "Back Injury" OR "Fracture" OR "Hand Injury" OR "Hip Injury" OR "Joint Dislocations" OR "Leg Injury" OR "Neck Injury" OR "Shoulder Injury" OR "Sprain" OR "Tendon Injury" OR "Ankle Fractures" OR "Ankle Injuries" OR "Arm Injuries" OR "Back Injuries" OR "Bone Diastasis" OR "Elbow Fractures" OR "Elbow Injuries" OR "Femoral Fractures" OR "Fibula Fractures" OR "Finger Injuries" OR "Foot Injuries" OR "Forearm Injuries" OR "Fracture" OR "Fracture Dislocation" OR "Fractures" OR "Hand Injuries" OR "Hip Dislocation" OR "Hip Fractures" OR "Hip Injuries" OR "Humeral Fractures" OR "Intra-Articular Fractures" OR "Joint Dislocations" OR "Knee Dislocation" OR "Knee Fractures" OR "Knee Injuries" OR "Leg Injuries" OR "Medial Tibial Stress Syndrome" OR "Muscle Diastasis" OR "Neck Injuries" OR "Osteoporotic Fractures" OR "Patellar Dislocation" OR "Periprosthetic Fractures" OR "Radius Fractures" OR "Rib Fractures" OR "Rotator Cuff Injuries" OR "Shoulder Dislocation" OR "Shoulder Fractures" OR "Shoulder Impingement Syndrome" OR "Shoulder Injuries" OR "Skull Fractures" OR "Spinal Fractures" OR "Spinal Injuries" OR "Sprain" OR "Tendinopathy" OR "Tendon Injuries" OR "Tibial Fractures" OR "Tibial Meniscus Injuries" OR "Traumatic Amputation" OR "Traumatic Multiple Amputations" OR "Ulna Fractures" OR "Whiplash Injuries" OR "Wrist Fractures" OR "Wrist Injuries" OR "Ankle Fracture" OR "Ankle Injury" OR "Arm Injury" OR "Back Injury" OR "Elbow Fracture" OR "Elbow Injury" OR "Femoral Fracture" OR "Fibula Fracture" OR "Finger Injury" OR "Foot Injury" OR "Forearm Injury" OR "Fracture Dislocations" OR "Hand Injury" OR "Hip Dislocations" OR "Hip Fracture" OR "Hip Injury" OR "Humeral Fracture" OR "Humeral Fracture" OR "Intra-Articular Fracture" OR "Joint Dislocation" OR "Knee Dislocations" OR "Knee Fracture" OR "Knee Injury" OR "Leg Injury" OR "Neck Injury" OR "Osteoporotic Fracture" OR "Patellar Dislocations" OR "Periprosthetic Fracture" OR "Radius Fracture" OR "Rib Fracture" OR "Rotator Cuff Injury" OR "Shoulder Dislocations" OR "Shoulder Fracture" OR "Shoulder Impingement" OR "Shoulder Injury" OR "Skull Fracture" OR "Spinal Fracture" OR "Spinal Injury" OR "Sprains" OR "Tendinopathies" OR "Tendon Injury" OR "Tibial Fracture" OR "Tibial Meniscus Injury" OR "Traumatic Amputations" OR "Traumatic Multiple Amputation" OR "Ulna Fracture" OR "Whiplash Injury" OR "Wrist Fracture" OR "Wrist Injury" OR "Orthopedics" OR "Orthopedic Surgery" OR "Acetabuloplast*" OR "Acetabuloplasty" OR "Alveolar Bone Graft*" OR "Alveolar Bone Grafting" OR "Ankle Replacement" OR "Anterior Cruciate Ligament Reconstruction" OR "Arthrodesis" OR "Arthroplast*" OR "Arthroplasty" OR "Arthroscop*" OR "Arthroscopy" OR "Bone Lengthening" OR "Bone Transplant*" OR "Bone Transplantation" OR "Bone-Patellar Tendon-Bone Graft*" OR "Bone-Patellar Tendon-Bone Grafting" OR "Cementoplast*" OR "Cementoplasty" OR "Disarticulation" OR "Diskectom*" OR "Diskectomy" OR "Distraction Osteogenesis" OR "Elbow Replacement" OR "Finger Replacement" OR "Fracture Fixation" OR "Genioplast*" OR "Genioplasty" OR "Hemiarthroplast*" OR "Hemiarthroplasty" OR "Hemipelvectom*" OR "Hemipelvectomy" OR "Hip Replacement" OR "Ilizarov Technique" OR "Joint Capsule Release" OR "Knee Replacement" OR "Kyphoplast*" OR "Kyphoplasty" OR "Laminectom*" OR "Laminectomy" OR "Le Fort Osteotom*" OR "Le Fort Osteotomy" OR "Limb Salvage" OR "Mandibular Osteotom*" OR "Mandibular Osteotomy" OR "Mandibular Reconstruction" OR "Maxillary Osteotom*" OR "Maxillary Osteotomy" OR "Meniscectom*" OR "Meniscectomy" OR "Open Fracture Reduction" OR "Orthognathic Surgical Procedure" OR "Orthognathic Surgical Procedures" OR "Osteotom*" OR "Osteotomy" OR "Posterior Cruciate Ligament Reconstruction" OR "Replacement Arthroplast*" OR "Replacement Arthroplasty" OR "Sagittal Split Ramus Osteotom*" OR "Sagittal Split Ramus Osteotomy" OR "Shoulder Replacement" OR "Sinus Floor Augmentation" OR "Spinal Fusion" OR "Surgical Amputation" OR "Synovectom*" OR "Synovectomy" OR "Tendon Transfer" OR "Tenodesis" OR "Tenotom*" OR "Tenotomy" OR "Total Disc Replacement" OR "Traction" OR "Ulnar Collateral Ligament Reconstruction" OR "Vertebroplast*" OR "Vertebroplasty" OR "Orthopedic Equipment" OR "orthopedic" OR "orthopedics" OR "orthopedic*" OR "orthopaedic" OR "orthopaedics" OR "orthopaedic*") OR MJ("orthopedic trauma" OR "orthopedic traumas" OR "orthopaedic trauma" OR "orthopaedic traumas" OR "orthopedic injury" OR "orthopedic injuries" OR "orthopaedic injury" OR "orthopaedic injuries" OR "orthopedic surgery" OR "orthopedic surgeries" OR "orthopaedic surgery" OR "orthopaedic surgeries" OR "orthopedic intervention" OR "orthopedic interventions" OR "orthopaedic intervention" OR "orthopaedic interventions" OR "orthopedic trauma" OR "orthopedic procedures" OR "orthopaedic procedure" OR "orthopaedic procedures" OR "Arm Injury" OR "Back Injury" OR "Fracture" OR "Hand Injury" OR "Hip Injury" OR "Joint Dislocations" OR "Leg Injury" OR "Neck Injury" OR "Shoulder Injury" OR "Sprain" OR "Tendon Injury" OR "Ankle Fractures" OR "Ankle Injuries" OR "Arm Injuries" OR "Back Injuries" OR "Bone Diastasis" OR "Elbow Fractures" OR "Elbow Injuries" OR "Femoral Fractures" OR "Fibula Fractures" OR "Finger Injuries" OR "Foot Injuries" OR "Forearm Injuries" OR "Fracture" OR "Fracture Dislocation" OR "Fractures" OR "Hand Injuries" OR "Hip Dislocation" OR "Hip Fractures" OR "Hip Injuries" OR "Humeral Fractures" OR "Intra-Articular Fractures" OR "Joint Dislocations" OR "Knee Dislocation" OR "Knee Fractures" OR "Knee Injuries" OR "Leg Injuries" OR "Medial Tibial Stress Syndrome" OR "Muscle Diastasis" OR "Neck Injuries" OR "Osteoporotic Fractures" OR "Patellar Dislocation" OR "Periprosthetic Fractures" OR "Radius Fractures" OR "Rib Fractures" OR "Rotator Cuff Injuries" OR "Shoulder Dislocation" OR "Shoulder Fractures" OR "Shoulder Impingement Syndrome" OR "Shoulder Injuries" OR "Skull Fractures" OR "Spinal Fractures" OR "Spinal Injuries" OR "Sprain" OR "Tendinopathy" OR "Tendon Injuries" OR "Tibial Fractures" OR "Tibial Meniscus Injuries" OR "Traumatic Amputation" OR "Traumatic Multiple Amputations" OR "Ulna Fractures" OR "Whiplash Injuries" OR "Wrist Fractures" OR "Wrist Injuries" OR "Ankle Fracture" OR "Ankle Injury" OR "Arm Injury" OR "Back Injury" OR "Elbow Fracture" OR "Elbow Injury" OR "Femoral Fracture" OR "Fibula Fracture" OR "Finger Injury" OR "Foot Injury" OR "Forearm Injury" OR "Fracture Dislocations" OR "Hand Injury" OR "Hip Dislocations" OR "Hip Fracture" OR "Hip Injury" OR "Humeral Fracture" OR "Humeral Fracture" OR "Intra-Articular Fracture" OR "Joint Dislocation" OR "Knee Dislocations" OR "Knee Fracture" OR "Knee Injury" OR "Leg Injury" OR "Neck Injury" OR "Osteoporotic Fracture" OR "Patellar Dislocations" OR "Periprosthetic Fracture" OR "Radius Fracture" OR "Rib Fracture" OR "Rotator Cuff Injury" OR "Shoulder Dislocations" OR "Shoulder Fracture" OR "Shoulder Impingement" OR "Shoulder Injury" OR "Skull Fracture" OR "Spinal Fracture" OR "Spinal Injury" OR "Sprains" OR "Tendinopathies" OR "Tendon Injury" OR "Tibial Fracture" OR "Tibial Meniscus Injury" OR "Traumatic Amputations" OR "Traumatic Multiple Amputation" OR "Ulna Fracture" OR "Whiplash Injury" OR "Wrist Fracture" OR "Wrist Injury" OR "Orthopedics" OR "Orthopedic Surgery" OR "Acetabuloplast*" OR "Acetabuloplasty" OR "Alveolar Bone Graft*" OR "Alveolar Bone Grafting" OR "Ankle Replacement" OR "Anterior Cruciate Ligament Reconstruction" OR "Arthrodesis" OR "Arthroplast*" OR "Arthroplasty" OR "Arthroscop*" OR "Arthroscopy" OR "Bone Lengthening" OR "Bone Transplant*" OR "Bone Transplantation" OR "Bone-Patellar Tendon-Bone Graft*" OR "Bone-Patellar Tendon-Bone Grafting" OR "Cementoplast*" OR "Cementoplasty" OR "Disarticulation" OR "Diskectom*" OR "Diskectomy" OR "Distraction Osteogenesis" OR "Elbow Replacement" OR "Finger Replacement" OR "Fracture Fixation" OR "Genioplast*" OR "Genioplasty" OR "Hemiarthroplast*" OR "Hemiarthroplasty" OR "Hemipelvectom*" OR "Hemipelvectomy" OR "Hip Replacement" OR "Ilizarov Technique" OR "Joint Capsule Release" OR "Knee Replacement" OR "Kyphoplast*" OR "Kyphoplasty" OR "Laminectom*" OR "Laminectomy" OR "Le Fort Osteotom*" OR "Le Fort Osteotomy" OR "Limb Salvage" OR "Mandibular Osteotom*" OR "Mandibular Osteotomy" OR "Mandibular Reconstruction" OR "Maxillary Osteotom*" OR "Maxillary Osteotomy" OR "Meniscectom*" OR "Meniscectomy" OR "Open Fracture Reduction" OR "Orthognathic Surgical Procedure" OR "Orthognathic Surgical Procedures" OR "Osteotom*" OR "Osteotomy" OR "Posterior Cruciate Ligament Reconstruction" OR "Replacement Arthroplast*" OR "Replacement Arthroplasty" OR "Sagittal Split Ramus Osteotom*" OR "Sagittal Split Ramus Osteotomy" OR "Shoulder Replacement" OR "Sinus Floor Augmentation" OR "Spinal Fusion" OR "Surgical Amputation" OR "Synovectom*" OR "Synovectomy" OR "Tendon Transfer" OR "Tenodesis" OR "Tenotom*" OR "Tenotomy" OR "Total Disc Replacement" OR "Traction" OR "Ulnar Collateral Ligament Reconstruction" OR "Vertebroplast*" OR "Vertebroplasty" OR "Orthopedic Equipment" OR "orthopedic" OR "orthopedics" OR "orthopedic*" OR "orthopaedic" OR "orthopaedics" OR "orthopaedic*") OR MA("orthopedic trauma" OR "orthopedic traumas" OR "orthopaedic trauma" OR "orthopaedic traumas" OR "orthopedic injury" OR "orthopedic injuries" OR "orthopaedic injury" OR "orthopaedic injuries" OR "orthopedic surgery" OR "orthopedic surgeries" OR "orthopaedic surgery" OR "orthopaedic surgeries" OR "orthopedic intervention" OR "orthopedic interventions" OR "orthopaedic intervention" OR "orthopaedic interventions" OR "orthopedic trauma" OR "orthopedic procedures" OR "orthopaedic procedure" OR "orthopaedic procedures" OR "Arm Injury" OR "Back Injury" OR "Fracture" OR "Hand Injury" OR "Hip Injury" OR "Joint Dislocations" OR "Leg Injury" OR "Neck Injury" OR "Shoulder Injury" OR "Sprain" OR "Tendon Injury" OR "Ankle Fractures" OR "Ankle Injuries" OR "Arm Injuries" OR "Back Injuries" OR "Bone Diastasis" OR "Elbow Fractures" OR "Elbow Injuries" OR "Femoral Fractures" OR "Fibula Fractures" OR "Finger Injuries" OR "Foot Injuries" OR "Forearm Injuries" OR "Fracture" OR "Fracture Dislocation" OR "Fractures" OR "Hand Injuries" OR "Hip Dislocation" OR "Hip Fractures" OR "Hip Injuries" OR "Humeral Fractures" OR "Intra-Articular Fractures" OR "Joint Dislocations" OR "Knee Dislocation" OR "Knee Fractures" OR "Knee Injuries" OR "Leg Injuries" OR "Medial Tibial Stress Syndrome" OR "Muscle Diastasis" OR "Neck Injuries" OR "Osteoporotic Fractures" OR "Patellar Dislocation" OR "Periprosthetic Fractures" OR "Radius Fractures" OR "Rib Fractures" OR "Rotator Cuff Injuries" OR "Shoulder Dislocation" OR "Shoulder Fractures" OR "Shoulder Impingement Syndrome" OR "Shoulder Injuries" OR "Skull Fractures" OR "Spinal Fractures" OR "Spinal Injuries" OR "Sprain" OR "Tendinopathy" OR "Tendon Injuries" OR "Tibial Fractures" OR "Tibial Meniscus Injuries" OR "Traumatic Amputation" OR "Traumatic Multiple Amputations" OR "Ulna Fractures" OR "Whiplash Injuries" OR "Wrist Fractures" OR "Wrist Injuries" OR "Ankle Fracture" OR "Ankle Injury" OR "Arm Injury" OR "Back Injury" OR "Elbow Fracture" OR "Elbow Injury" OR "Femoral Fracture" OR "Fibula Fracture" OR "Finger Injury" OR "Foot Injury" OR "Forearm Injury" OR "Fracture Dislocations" OR "Hand Injury" OR "Hip Dislocations" OR "Hip Fracture" OR "Hip Injury" OR "Humeral Fracture" OR "Humeral Fracture" OR "Intra-Articular Fracture" OR "Joint Dislocation" OR "Knee Dislocations" OR "Knee Fracture" OR "Knee Injury" OR "Leg Injury" OR "Neck Injury" OR "Osteoporotic Fracture" OR "Patellar Dislocations" OR "Periprosthetic Fracture" OR "Radius Fracture" OR "Rib Fracture" OR "Rotator Cuff Injury" OR "Shoulder Dislocations" OR "Shoulder Fracture" OR "Shoulder Impingement" OR "Shoulder Injury" OR "Skull Fracture" OR "Spinal Fracture" OR "Spinal Injury" OR "Sprains" OR "Tendinopathies" OR "Tendon Injury" OR "Tibial Fracture" OR "Tibial Meniscus Injury" OR "Traumatic Amputations" OR "Traumatic Multiple Amputation" OR "Ulna Fracture" OR "Whiplash Injury" OR "Wrist Fracture" OR "Wrist Injury" OR "Orthopedics" OR "Orthopedic Surgery" OR "Acetabuloplast*" OR "Acetabuloplasty" OR "Alveolar Bone Graft*" OR "Alveolar Bone Grafting" OR "Ankle Replacement" OR "Anterior Cruciate Ligament Reconstruction" OR "Arthrodesis" OR "Arthroplast*" OR "Arthroplasty" OR "Arthroscop*" OR "Arthroscopy" OR "Bone Lengthening" OR "Bone Transplant*" OR "Bone Transplantation" OR "Bone-Patellar Tendon-Bone Graft*" OR "Bone-Patellar Tendon-Bone Grafting" OR "Cementoplast*" OR "Cementoplasty" OR "Disarticulation" OR "Diskectom*" OR "Diskectomy" OR "Distraction Osteogenesis" OR "Elbow Replacement" OR "Finger Replacement" OR "Fracture Fixation" OR "Genioplast*" OR "Genioplasty" OR "Hemiarthroplast*" OR "Hemiarthroplasty" OR "Hemipelvectom*" OR "Hemipelvectomy" OR "Hip Replacement" OR "Ilizarov Technique" OR "Joint Capsule Release" OR "Knee Replacement" OR "Kyphoplast*" OR "Kyphoplasty" OR "Laminectom*" OR "Laminectomy" OR "Le Fort Osteotom*" OR "Le Fort Osteotomy" OR "Limb Salvage" OR "Mandibular Osteotom*" OR "Mandibular Osteotomy" OR "Mandibular Reconstruction" OR "Maxillary Osteotom*" OR "Maxillary Osteotomy" OR "Meniscectom*" OR "Meniscectomy" OR "Open Fracture Reduction" OR "Orthognathic Surgical Procedure" OR "Orthognathic Surgical Procedures" OR "Osteotom*" OR "Osteotomy" OR "Posterior Cruciate Ligament Reconstruction" OR "Replacement Arthroplast*" OR "Replacement Arthroplasty" OR "Sagittal Split Ramus Osteotom*" OR "Sagittal Split Ramus Osteotomy" OR "Shoulder Replacement" OR "Sinus Floor Augmentation" OR "Spinal Fusion" OR "Surgical Amputation" OR "Synovectom*" OR "Synovectomy" OR "Tendon Transfer" OR "Tenodesis" OR "Tenotom*" OR "Tenotomy" OR "Total Disc Replacement" OR "Traction" OR "Ulnar Collateral Ligament Reconstruction" OR "Vertebroplast*" OR "Vertebroplasty" OR "Orthopedic Equipment" OR "orthopedic" OR "orthopedics" OR "orthopedic*" OR "orthopaedic" OR "orthopaedics" OR "orthopaedic*") OR AB("orthopedic trauma" OR "orthopedic traumas" OR "orthopaedic trauma" OR "orthopaedic traumas" OR "orthopedic injury" OR "orthopedic injuries" OR "orthopaedic injury" OR "orthopaedic injuries" OR "orthopedic surgery" OR "orthopedic surgeries" OR "orthopaedic surgery" OR "orthopaedic surgeries" OR "orthopedic intervention" OR "orthopedic interventions" OR "orthopaedic intervention" OR "orthopaedic interventions" OR "orthopedic trauma" OR "orthopedic procedures" OR "orthopaedic procedure" OR "orthopaedic procedures" OR "Arm Injury" OR "Back Injury" OR "Fracture" OR "Hand Injury" OR "Hip Injury" OR "Joint Dislocations" OR "Leg Injury" OR "Neck Injury" OR "Shoulder Injury" OR "Sprain" OR "Tendon Injury" OR "Ankle Fractures" OR "Ankle Injuries" OR "Arm Injuries" OR "Back Injuries" OR "Bone Diastasis" OR "Elbow Fractures" OR "Elbow Injuries" OR "Femoral Fractures" OR "Fibula Fractures" OR "Finger Injuries" OR "Foot Injuries" OR "Forearm Injuries" OR "Fracture" OR "Fracture Dislocation" OR "Fractures" OR "Hand Injuries" OR "Hip Dislocation" OR "Hip Fractures" OR "Hip Injuries" OR "Humeral Fractures" OR "Intra-Articular Fractures" OR "Joint Dislocations" OR "Knee Dislocation" OR "Knee Fractures" OR "Knee Injuries" OR "Leg Injuries" OR "Medial Tibial Stress Syndrome" OR "Muscle Diastasis" OR "Neck Injuries" OR "Osteoporotic Fractures" OR "Patellar Dislocation" OR "Periprosthetic Fractures" OR "Radius Fractures" OR "Rib Fractures" OR "Rotator Cuff Injuries" OR "Shoulder Dislocation" OR "Shoulder Fractures" OR "Shoulder Impingement Syndrome" OR "Shoulder Injuries" OR "Skull Fractures" OR "Spinal Fractures" OR "Spinal Injuries" OR "Sprain" OR "Tendinopathy" OR "Tendon Injuries" OR "Tibial Fractures" OR "Tibial Meniscus Injuries" OR "Traumatic Amputation" OR "Traumatic Multiple Amputations" OR "Ulna Fractures" OR "Whiplash Injuries" OR "Wrist Fractures" OR "Wrist Injuries" OR "Ankle Fracture" OR "Ankle Injury" OR "Arm Injury" OR "Back Injury" OR "Elbow Fracture" OR "Elbow Injury" OR "Femoral Fracture" OR "Fibula Fracture" OR "Finger Injury" OR "Foot Injury" OR "Forearm Injury" OR "Fracture Dislocations" OR "Hand Injury" OR "Hip Dislocations" OR "Hip Fracture" OR "Hip Injury" OR "Humeral Fracture" OR "Humeral Fracture" OR "Intra-Articular Fracture" OR "Joint Dislocation" OR "Knee Dislocations" OR "Knee Fracture" OR "Knee Injury" OR "Leg Injury" OR "Neck Injury" OR "Osteoporotic Fracture" OR "Patellar Dislocations" OR "Periprosthetic Fracture" OR "Radius Fracture" OR "Rib Fracture" OR "Rotator Cuff Injury" OR "Shoulder Dislocations" OR "Shoulder Fracture" OR "Shoulder Impingement" OR "Shoulder Injury" OR "Skull Fracture" OR "Spinal Fracture" OR "Spinal Injury" OR "Sprains" OR "Tendinopathies" OR "Tendon Injury" OR "Tibial Fracture" OR "Tibial Meniscus Injury" OR "Traumatic Amputations" OR "Traumatic Multiple Amputation" OR "Ulna Fracture" OR "Whiplash Injury" OR "Wrist Fracture" OR "Wrist Injury" OR "Orthopedics" OR "Orthopedic Surgery" OR "Acetabuloplast*" OR "Acetabuloplasty" OR "Alveolar Bone Graft*" OR "Alveolar Bone Grafting" OR "Ankle Replacement" OR "Anterior Cruciate Ligament Reconstruction" OR "Arthrodesis" OR "Arthroplast*" OR "Arthroplasty" OR "Arthroscop*" OR "Arthroscopy" OR "Bone Lengthening" OR "Bone Transplant*" OR "Bone Transplantation" OR "Bone-Patellar Tendon-Bone Graft*" OR "Bone-Patellar Tendon-Bone Grafting" OR "Cementoplast*" OR "Cementoplasty" OR "Disarticulation" OR "Diskectom*" OR "Diskectomy" OR "Distraction Osteogenesis" OR "Elbow Replacement" OR "Finger Replacement" OR "Fracture Fixation" OR "Genioplast*" OR "Genioplasty" OR "Hemiarthroplast*" OR "Hemiarthroplasty" OR "Hemipelvectom*" OR "Hemipelvectomy" OR "Hip Replacement" OR "Ilizarov Technique" OR "Joint Capsule Release" OR "Knee Replacement" OR "Kyphoplast*" OR "Kyphoplasty" OR "Laminectom*" OR "Laminectomy" OR "Le Fort Osteotom*" OR "Le Fort Osteotomy" OR "Limb Salvage" OR "Mandibular Osteotom*" OR "Mandibular Osteotomy" OR "Mandibular Reconstruction" OR "Maxillary Osteotom*" OR "Maxillary Osteotomy" OR "Meniscectom*" OR "Meniscectomy" OR "Open Fracture Reduction" OR "Orthognathic Surgical Procedure" OR "Orthognathic Surgical Procedures" OR "Osteotom*" OR "Osteotomy" OR "Posterior Cruciate Ligament Reconstruction" OR "Replacement Arthroplast*" OR "Replacement Arthroplasty" OR "Sagittal Split Ramus Osteotom*" OR "Sagittal Split Ramus Osteotomy" OR "Shoulder Replacement" OR "Sinus Floor Augmentation" OR "Spinal Fusion" OR "Surgical Amputation" OR "Synovectom*" OR "Synovectomy" OR "Tendon Transfer" OR "Tenodesis" OR "Tenotom*" OR "Tenotomy" OR "Total Disc Replacement" OR "Traction" OR "Ulnar Collateral Ligament Reconstruction" OR "Vertebroplast*" OR "Vertebroplasty" OR "Orthopedic Equipment" OR "orthopedic" OR "orthopedics" OR "orthopedic*" OR "orthopaedic" OR "orthopaedics" OR "orthopaedic*")))

**PubMed**

(("Stress Disorders, Post-Traumatic"[Mesh] OR "Moral Injuries"[tw] OR "Moral Injury"[tw] OR "Post Traumatic Neuroses"[tw] OR "Post Traumatic Neurosis"[tw] OR "Post Traumatic Stress"[tw] OR "Post Traumatic Stress Disorder"[tw] OR "Post Traumatic Stress Disorders"[tw] OR "Posttraumatic Neuroses"[tw] OR "Posttraumatic Neurosis"[tw] OR "Posttraumatic Stress"[tw] OR "Posttraumatic Stress Disorder"[tw] OR "Posttraumatic Stress Disorders"[tw] OR "PTSD stress"[title/abstract:~10] OR "PTSS stress"[title/abstract:~10] OR "Moral Injuries"[title/abstract:~6] OR "Moral Injury"[title/abstract:~6] OR "Post Traumatic Neuroses"[title/abstract:~6] OR "Post Traumatic Neurosis"[title/abstract:~6] OR "Post Traumatic Stress"[title/abstract:~6] OR "Post Traumatic Stress Disorder"[title/abstract:~6] OR "Post Traumatic Stress Disorders"[title/abstract:~6] OR "Posttraumatic Neuroses"[title/abstract:~6] OR "Posttraumatic Neurosis"[title/abstract:~6] OR "Posttraumatic Stress"[title/abstract:~6] OR "Posttraumatic Stress Disorder"[title/abstract:~6] OR "Posttraumatic Stress Disorders"[title/abstract:~6]) AND ("Child"[Mesh] OR "child"[tw] OR "children"[tw] OR "Infant"[Mesh] OR "infant"[tw] OR "infants"[tw] OR "infancy"[tw] OR "newborn"[tw] OR "newborns"[tw] OR "new-born"[tw] OR "new-borns"[tw] OR "neonate"[tw] OR "neonates"[tw] OR "neonatal"[tw] OR "neo-nate"[tw] OR "neo-nates"[tw] OR "neo-natal"[tw] OR "neonatology"[tw] OR "NICU"[ti] OR "premature"[tw] OR "prematures"[tw] OR "pre-mature"[tw] OR "pre-matures"[tw] OR "preterm"[tw] OR "pre-term"[tw] OR "postnatal"[tw] OR "post-natal"[tw] OR "baby"[tw] OR "babies"[tw] OR "suckling"[tw] OR "sucklings"[tw] OR "toddler"[tw] OR "toddlers"[tw] OR "childhood"[tw] OR "schoolchild"[tw] OR "schoolchildren"[tw] OR "childcare"[tw] OR "child-care"[tw] OR "youngster"[tw] OR "youngsters"[tw] OR "preschool"[tw] OR "pre-school"[tw] OR "kid"[tw] OR "kids"[tw] OR "boy"[tw] OR "boys"[tw] OR "girl"[tw] OR "girls"[tw] OR "Adolescent"[Mesh] OR "adolescent"[tw] OR "adolescents"[tw] OR "adolescence"[tw] OR "pre-adolescent"[tw] OR "pre-adolescents"[tw] OR "pre-adolescence"[tw] OR "schoolage"[tw] OR "schoolboy"[tw] OR "schoolboys"[tw] OR "schoolgirl"[tw] OR "schoolgirls"[tw] OR "pre-puber"[tw] OR "pre-puberty"[tw] OR "prepuber"[tw] OR "prepubers"[tw] OR "prepuberty"[tw] OR "puber"[tw] OR "puberty"[tw] OR "puberal"[tw] OR "teenager"[tw] OR "teenagers"[tw] OR "teens"[tw] OR "youth"[tw] OR "youths"[tw] OR "underaged"[tw] OR "under-aged"[tw] OR "Pediatrics"[Mesh] OR "Pediatric"[tw] OR "Pediatrics"[tw] OR "Paediatric"[tw] OR "Paediatrics"[tw] OR children*[tw] OR schoolchild*[tw] OR "infant"[tw] OR "infants"[tw] OR "infancy"[tw] OR adolesc*[tw] OR pediat*[tw] OR paediat*[tw] OR neonat*[tw] OR toddler*[tw] OR "teen"[tw] OR "teens"[tw] OR teenager*[tw] OR preteen*[tw] OR newborn*[tw] OR postneonat*[tw] OR postnatal*[tw] OR "puberty"[tw] OR preschool*[tw] OR suckling*[tw] OR "juvenile"[tw] OR "new born"[tw] OR "new borns"[tw] OR new-born*[tw] OR neo-nat*[tw] OR neonat*[tw] OR perinat*[tw] OR underag*[tw] OR "under age"[tw] OR "under aged"[tw] OR youth*[tw] OR kinder*[tw] OR pubescen*[tw] OR prepubescen*[tw] OR "prepuberty"[tw] OR "school age"[tw] OR "schoolage"[tw] OR "school ages"[tw] OR schoolage*[tw] OR "one year old"[ti] OR "two year old"[ti] OR "three year old"[ti] OR "four year old"[ti] OR "five year old"[ti] OR "six year old"[ti] OR "seven year old"[ti] OR "eight year old"[ti] OR "nine year old"[ti] OR "ten year old"[ti] OR "eleven year old"[ti] OR "twelve year old"[ti] OR "thirteen year old"[ti] OR "fourteen year old"[ti] OR "fifteen year old"[ti] OR "sixteen year old"[ti] OR "seventeen year old"[ti] OR "eighteen year old"[ti] OR "1 year old"[ti] OR "2 year old"[ti] OR "3 year old"[ti] OR "4 year old"[ti] OR "5 year old"[ti] OR "6 year old"[ti] OR "7 year old"[ti] OR "8 year old"[ti] OR "9 year old"[ti] OR "10 year old"[ti] OR "11 year old"[ti] OR "12 year old"[ti] OR "13 year old"[ti] OR "14 year old"[ti] OR "15 year old"[ti] OR "16 year old"[ti] OR "17 year old"[ti] OR "18 year old"[ti] OR "two years old"[ti] OR "three years old"[ti] OR "four years old"[ti] OR "five years old"[ti] OR "six years old"[ti] OR "seven years old"[ti] OR "eight years old"[ti] OR "nine years old"[ti] OR "ten years old"[ti] OR "eleven years old"[ti] OR "twelve years old"[ti] OR "thirteen years old"[ti] OR "fourteen years old"[ti] OR "fifteen years old"[ti] OR "sixteen years old"[title:~0] OR "seventeen years old"[ti] OR "eighteen years old"[ti] OR "2 years old"[ti] OR "3 years old"[ti] OR "4 years old"[ti] OR "5 years old"[ti] OR "6 years old"[ti] OR "7 years old"[ti] OR "8 years old"[ti] OR "9 years old"[ti] OR "10 years old"[ti] OR "11 years old"[ti] OR "12 years old"[ti] OR "13 years old"[ti] OR "14 years old"[ti] OR "15 years old"[ti] OR "16 years old"[ti] OR "17 years old"[ti] OR "18 years old"[ti]) NOT 27885969[pmid] AND (("Wounds and Injuries"[Mesh] AND "Musculoskeletal System"[Mesh]) OR "orthopedic trauma"[tw] OR "orthopedic traumas"[tw] OR "orthopaedic trauma"[tw] OR "orthopaedic traumas"[tw] OR "orthopedic injury"[tw] OR "orthopedic injuries"[tw] OR "orthopaedic injury"[tw] OR "orthopaedic injuries"[tw] OR "orthopedic surgery"[tw] OR "orthopedic surgeries"[tw] OR "orthopaedic surgery"[tw] OR "orthopaedic surgeries"[tw] OR "orthopedic intervention"[tw] OR "orthopedic interventions"[tw] OR "orthopaedic intervention"[tw] OR "orthopaedic interventions"[tw] OR "orthopedic trauma"[tw] OR "orthopedic procedures"[tw] OR "orthopaedic procedure"[tw] OR "orthopaedic procedures"[tw] OR "Amputation, Traumatic"[mesh] OR "Arm Injuries"[mesh] OR "Back Injuries"[mesh] OR "Fractures, Bone"[mesh] OR "Hand Injuries"[mesh] OR "Hip Injuries"[mesh] OR "Joint Dislocations"[mesh] OR "Leg Injuries"[mesh] OR "Neck Injuries"[mesh] OR "Shoulder Injuries"[mesh] OR "Sprains and Strains"[mesh] OR "Tendon Injuries"[mesh] OR "Ankle Fractures"[tw] OR "Ankle Injuries"[tw] OR "Arm Injuries"[tw] OR "Back Injuries"[tw] OR "Bone Diastasis"[tw] OR "Elbow Fractures"[tw] OR "Elbow Injuries"[tw] OR "Femoral Fractures"[tw] OR "Fibula Fractures"[tw] OR "Finger Injuries"[tw] OR "Foot Injuries"[tw] OR "Forearm Injuries"[tw] OR "Fracture"[tw] OR "Fracture Dislocation"[tw] OR "Fractures"[tw] OR "Hand Injuries"[tw] OR "Hip Dislocation"[tw] OR "Hip Fractures"[tw] OR "Hip Injuries"[tw] OR "Humeral Fractures"[tw] OR "Intra-Articular Fractures"[tw] OR "Joint Dislocations"[tw] OR "Knee Dislocation"[tw] OR "Knee Fractures"[tw] OR "Knee Injuries"[tw] OR "Leg Injuries"[tw] OR "Medial Tibial Stress Syndrome"[tw] OR "Muscle Diastasis"[tw] OR "Neck Injuries"[tw] OR "Osteoporotic Fractures"[tw] OR "Patellar Dislocation"[tw] OR "Periprosthetic Fractures"[tw] OR "Radius Fractures"[tw] OR "Rib Fractures"[tw] OR "Rotator Cuff Injuries"[tw] OR "Shoulder Dislocation"[tw] OR "Shoulder Fractures"[tw] OR "Shoulder Impingement Syndrome"[tw] OR "Shoulder Injuries"[tw] OR "Skull Fractures"[tw] OR "Spinal Fractures"[tw] OR "Spinal Injuries"[tw] OR "Sprain"[tw] OR "Tendinopathy"[tw] OR "Tendon Injuries"[tw] OR "Tibial Fractures"[tw] OR "Tibial Meniscus Injuries"[tw] OR "Traumatic Amputation"[tw] OR "Traumatic Multiple Amputations"[title/abstract:~0] OR "Ulna Fractures"[tw] OR "Whiplash Injuries"[tw] OR "Wrist Fractures"[tw] OR "Wrist Injuries"[tw] OR "Ankle Fracture"[tw] OR "Ankle Injury"[tw] OR "Arm Injury"[tw] OR "Back Injury"[tw] OR "Elbow Fracture"[tw] OR "Elbow Injury"[tw] OR "Femoral Fracture"[tw] OR "Fibula Fracture"[tw] OR "Finger Injury"[tw] OR "Foot Injury"[tw] OR "Forearm Injury"[tw] OR "Fracture Dislocations"[tw] OR "Hand Injury"[tw] OR "Hip Dislocations"[tw] OR "Hip Fracture"[tw] OR "Hip Injury"[tw] OR "Humeral Fracture"[tw] OR "Humeral Fracture"[tw] OR "Intra-Articular Fracture"[tw] OR "Joint Dislocation"[tw] OR "Knee Dislocations"[tw] OR "Knee Fracture"[tw] OR "Knee Injury"[tw] OR "Leg Injury"[tw] OR "Neck Injury"[tw] OR "Osteoporotic Fracture"[tw] OR "Patellar Dislocations"[tw] OR "Periprosthetic Fracture"[tw] OR "Radius Fracture"[tw] OR "Rib Fracture"[tw] OR "Rotator Cuff Injury"[tw] OR "Shoulder Dislocations"[tw] OR "Shoulder Fracture"[tw] OR "Shoulder Impingement"[tw] OR "Shoulder Injury"[tw] OR "Skull Fracture"[tw] OR "Spinal Fracture"[tw] OR "Spinal Injury"[tw] OR "Sprains"[tw] OR "Tendinopathies"[tw] OR "Tendon Injury"[tw] OR "Tibial Fracture"[tw] OR "Tibial Meniscus Injury"[tw] OR "Traumatic Amputations"[tw] OR "Traumatic Multiple Amputation"[title/abstract:~0] OR "Ulna Fracture"[tw] OR "Whiplash Injury"[tw] OR "Wrist Fracture"[tw] OR "Wrist Injury"[tw] OR "Orthopedics"[Mesh] OR "Orthopedic Procedures"[Mesh] OR "Acetabuloplast*"[tw] OR "Acetabuloplasty"[tw] OR "Alveolar Bone Graft*"[tw] OR "Alveolar Bone Grafting"[tw] OR "Ankle Replacement"[tw] OR "Anterior Cruciate Ligament Reconstruction"[tw] OR "Arthrodesis"[tw] OR "Arthroplast*"[tw] OR "Arthroplasty"[tw] OR "Arthroscop*"[tw] OR "Arthroscopy"[tw] OR "Bone Lengthening"[tw] OR "Bone Transplant*"[tw] OR "Bone Transplantation"[tw] OR "Bone-Patellar Tendon-Bone Graft*"[tw] OR "Bone-Patellar Tendon-Bone Grafting"[tw] OR "Cementoplast*"[tw] OR "Cementoplasty"[tw] OR "Disarticulation"[tw] OR "Diskectom*"[tw] OR "Diskectomy"[tw] OR "Distraction Osteogenesis"[tw] OR "Elbow Replacement"[tw] OR "Finger Replacement"[tw] OR "Fracture Fixation"[tw] OR "Genioplast*"[tw] OR "Genioplasty"[tw] OR "Hemiarthroplast*"[tw] OR "Hemiarthroplasty"[tw] OR "Hemipelvectom*"[tw] OR "Hemipelvectomy"[tw] OR "Hip Replacement"[tw] OR "Ilizarov Technique"[tw] OR "Joint Capsule Release"[tw] OR "Knee Replacement"[tw] OR "Kyphoplast*"[tw] OR "Kyphoplasty"[tw] OR "Laminectom*"[tw] OR "Laminectomy"[tw] OR "Le Fort Osteotom*"[tw] OR "Le Fort Osteotomy"[tw] OR "Limb Salvage"[tw] OR "Mandibular Osteotom*"[tw] OR "Mandibular Osteotomy"[tw] OR "Mandibular Reconstruction"[tw] OR "Maxillary Osteotom*"[tw] OR "Maxillary Osteotomy"[tw] OR "Meniscectom*"[tw] OR "Meniscectomy"[tw] OR "Open Fracture Reduction"[tw] OR "Orthognathic Surgical Procedure"[tw] OR "Orthognathic Surgical Procedures"[tw] OR "Osteotom*"[tw] OR "Osteotomy"[tw] OR "Posterior Cruciate Ligament Reconstruction"[tw] OR "Replacement Arthroplast*"[tw] OR "Replacement Arthroplasty"[tw] OR "Sagittal Split Ramus Osteotom*"[tw] OR "Sagittal Split Ramus Osteotomy"[tw] OR "Shoulder Replacement"[tw] OR "Sinus Floor Augmentation"[tw] OR "Spinal Fusion"[tw] OR "Surgical Amputation"[tw] OR "Synovectom*"[tw] OR "Synovectomy"[tw] OR "Tendon Transfer"[tw] OR "Tenodesis"[tw] OR "Tenotom*"[tw] OR "Tenotomy"[tw] OR "Total Disc Replacement"[tw] OR "Traction"[tw] OR "Ulnar Collateral Ligament Reconstruction"[tw] OR "Vertebroplast*"[tw] OR "Vertebroplasty"[tw] OR "Orthopedic Equipment"[Mesh] OR "orthopedic"[tw] OR "orthopedics"[tw] OR "orthopedic*"[tw] OR "orthopaedic"[tw] OR "orthopaedics"[tw] OR "orthopaedic*"[tw]))

**Web of Science**

((TI=("posttraumatic stress disorder" OR "Moral Injuries" OR "Moral Injury" OR "Post Traumatic Neuroses" OR "Post Traumatic Neurosis" OR "Post Traumatic Stress" OR "Post Traumatic Stress Disorder" OR "Post Traumatic Stress Disorders" OR "Posttraumatic Neuroses" OR "Posttraumatic Neurosis" OR "Posttraumatic Stress" OR "Posttraumatic Stress Disorder" OR "Posttraumatic Stress Disorders" OR (("PTSD" NEAR/10 "stress") OR ("PTSS" NEAR/10 "stress")) OR (("Moral" NEAR/6 "Injuries") OR ("Moral" NEAR/6 "Injury") OR ("Post" NEAR/6 "Traumatic" NEAR/6 "Neuroses") OR ("Post" NEAR/6 "Traumatic" NEAR/6 "Neurosis") OR ("Post" NEAR/6 "Traumatic" NEAR/6 "Stress") OR ("Post" NEAR/6 "Traumatic" NEAR/6 "Stress" NEAR/6 "Disorder") OR ("Post" NEAR/6 "Traumatic" NEAR/6 "Stress" NEAR/6 "Disorders") OR ("Posttraumatic" NEAR/6 "Neuroses") OR ("Posttraumatic" NEAR/6 "Neurosis") OR ("Posttraumatic" NEAR/6 "Stress") OR ("Posttraumatic" NEAR/6 "Stress" NEAR/6 "Disorder") OR ("Posttraumatic" NEAR/6 "Stress" NEAR/6 "Disorders"))) OR AK=("posttraumatic stress disorder" OR "Moral Injuries" OR "Moral Injury" OR "Post Traumatic Neuroses" OR "Post Traumatic Neurosis" OR "Post Traumatic Stress" OR "Post Traumatic Stress Disorder" OR "Post Traumatic Stress Disorders" OR "Posttraumatic Neuroses" OR "Posttraumatic Neurosis" OR "Posttraumatic Stress" OR "Posttraumatic Stress Disorder" OR "Posttraumatic Stress Disorders" OR (("PTSD" NEAR/10 "stress") OR ("PTSS" NEAR/10 "stress")) OR (("Moral" NEAR/6 "Injuries") OR ("Moral" NEAR/6 "Injury") OR ("Post" NEAR/6 "Traumatic" NEAR/6 "Neuroses") OR ("Post" NEAR/6 "Traumatic" NEAR/6 "Neurosis") OR ("Post" NEAR/6 "Traumatic" NEAR/6 "Stress") OR ("Post" NEAR/6 "Traumatic" NEAR/6 "Stress" NEAR/6 "Disorder") OR ("Post" NEAR/6 "Traumatic" NEAR/6 "Stress" NEAR/6 "Disorders") OR ("Posttraumatic" NEAR/6 "Neuroses") OR ("Posttraumatic" NEAR/6 "Neurosis") OR ("Posttraumatic" NEAR/6 "Stress") OR ("Posttraumatic" NEAR/6 "Stress" NEAR/6 "Disorder") OR ("Posttraumatic" NEAR/6 "Stress" NEAR/6 "Disorders"))) OR AB=("posttraumatic stress disorder" OR "Moral Injuries" OR "Moral Injury" OR "Post Traumatic Neuroses" OR "Post Traumatic Neurosis" OR "Post Traumatic Stress" OR "Post Traumatic Stress Disorder" OR "Post Traumatic Stress Disorders" OR "Posttraumatic Neuroses" OR "Posttraumatic Neurosis" OR "Posttraumatic Stress" OR "Posttraumatic Stress Disorder" OR "Posttraumatic Stress Disorders" OR (("PTSD" NEAR/10 "stress") OR ("PTSS" NEAR/10 "stress")) OR (("Moral" NEAR/6 "Injuries") OR ("Moral" NEAR/6 "Injury") OR ("Post" NEAR/6 "Traumatic" NEAR/6 "Neuroses") OR ("Post" NEAR/6 "Traumatic" NEAR/6 "Neurosis") OR ("Post" NEAR/6 "Traumatic" NEAR/6 "Stress") OR ("Post" NEAR/6 "Traumatic" NEAR/6 "Stress" NEAR/6 "Disorder") OR ("Post" NEAR/6 "Traumatic" NEAR/6 "Stress" NEAR/6 "Disorders") OR ("Posttraumatic" NEAR/6 "Neuroses") OR ("Posttraumatic" NEAR/6 "Neurosis") OR ("Posttraumatic" NEAR/6 "Stress") OR ("Posttraumatic" NEAR/6 "Stress" NEAR/6 "Disorder") OR ("Posttraumatic" NEAR/6 "Stress" NEAR/6 "Disorders")))) AND (TI=("Child" OR "child" OR "children" OR exp "Infant" OR "infant" OR "infants" OR "infancy" OR "newborn" OR "newborns" OR "new-born" OR "new-borns" OR "neonate" OR "neonates" OR "neonatal" OR "neo-nate" OR "neo-nates" OR "neo-natal" OR "neonatology" OR "NICU" OR "premature" OR "prematures" OR "pre-mature" OR "pre-matures" OR "preterm" OR "pre-term" OR "postnatal" OR "post-natal" OR "baby" OR "babies" OR "suckling" OR "sucklings" OR "toddler" OR "toddlers" OR "childhood" OR "schoolchild" OR "schoolchildren" OR "childcare" OR "child-care" OR "youngster" OR "youngsters" OR "preschool" OR "pre-school" OR "kid" OR "kids" OR "boy" OR "boys" OR "girl" OR "girls" OR exp "Adolescent" OR "adolescent" OR "adolescents" OR "adolescence" OR "pre-adolescent" OR "pre-adolescents" OR "pre-adolescence" OR "schoolage" OR "schoolboy" OR "schoolboys" OR "schoolgirl" OR "schoolgirls" OR "pre-puber" OR "pre-puberty" OR "prepuber" OR "prepubers" OR "prepuberty" OR "puber" OR "puberty" OR "puberal" OR "teenager" OR "teenagers" OR "teens" OR "youth" OR "youths" OR "underaged" OR "under-aged" OR exp "Pediatrics" OR "Pediatric" OR "Pediatrics" OR "Paediatric" OR "Paediatrics" **OR** "child" OR children* OR schoolchild* OR "infant" OR "infants" OR "infancy" OR adolesc* OR pediat* OR paediat* OR neonat* OR toddler* OR "teen" OR "teens" OR teenager* OR preteen* OR newborn* OR postneonat* OR postnatal* OR "puberty" OR preschool* OR suckling* OR "juvenile" OR "new born" OR "new borns" OR new-born* OR neo-nat* OR neonat* OR perinat* OR underag* OR "under age" OR "under aged" OR youth* OR kinder* OR pubescen* OR prepubescen* OR "prepuberty" OR "school age" OR "schoolage" OR "school ages" OR schoolage*) OR AK=("Child" OR "child" OR "children" OR exp "Infant" OR "infant" OR "infants" OR "infancy" OR "newborn" OR "newborns" OR "new-born" OR "new-borns" OR "neonate" OR "neonates" OR "neonatal" OR "neo-nate" OR "neo-nates" OR "neo-natal" OR "neonatology" OR "NICU" OR "premature" OR "prematures" OR "pre-mature" OR "pre-matures" OR "preterm" OR "pre-term" OR "postnatal" OR "post-natal" OR "baby" OR "babies" OR "suckling" OR "sucklings" OR "toddler" OR "toddlers" OR "childhood" OR "schoolchild" OR "schoolchildren" OR "childcare" OR "child-care" OR "youngster" OR "youngsters" OR "preschool" OR "pre-school" OR "kid" OR "kids" OR "boy" OR "boys" OR "girl" OR "girls" OR exp "Adolescent" OR "adolescent" OR "adolescents" OR "adolescence" OR "pre-adolescent" OR "pre-adolescents" OR "pre-adolescence" OR "schoolage" OR "schoolboy" OR "schoolboys" OR "schoolgirl" OR "schoolgirls" OR "pre-puber" OR "pre-puberty" OR "prepuber" OR "prepubers" OR "prepuberty" OR "puber" OR "puberty" OR "puberal" OR "teenager" OR "teenagers" OR "teens" OR "youth" OR "youths" OR "underaged" OR "under-aged" OR exp "Pediatrics" OR "Pediatric" OR "Pediatrics" OR "Paediatric" OR "Paediatrics" **OR** "child" OR children* OR schoolchild* OR "infant" OR "infants" OR "infancy" OR adolesc* OR pediat* OR paediat* OR neonat* OR toddler* OR "teen" OR "teens" OR teenager* OR preteen* OR newborn* OR postneonat* OR postnatal* OR "puberty" OR preschool* OR suckling* OR "juvenile" OR "new born" OR "new borns" OR new-born* OR neo-nat* OR neonat* OR perinat* OR underag* OR "under age" OR "under aged" OR youth* OR kinder* OR pubescen* OR prepubescen* OR "prepuberty" OR "school age" OR "schoolage" OR "school ages" OR schoolage*) OR AB=("Child" OR "child" OR "children" OR exp "Infant" OR "infant" OR "infants" OR "infancy" OR "newborn" OR "newborns" OR "new-born" OR "new-borns" OR "neonate" OR "neonates" OR "neonatal" OR "neo-nate" OR "neo-nates" OR "neo-natal" OR "neonatology" OR "NICU" OR "premature" OR "prematures" OR "pre-mature" OR "pre-matures" OR "preterm" OR "pre-term" OR "postnatal" OR "post-natal" OR "baby" OR "babies" OR "suckling" OR "sucklings" OR "toddler" OR "toddlers" OR "childhood" OR "schoolchild" OR "schoolchildren" OR "childcare" OR "child-care" OR "youngster" OR "youngsters" OR "preschool" OR "pre-school" OR "kid" OR "kids" OR "boy" OR "boys" OR "girl" OR "girls" OR exp "Adolescent" OR "adolescent" OR "adolescents" OR "adolescence" OR "pre-adolescent" OR "pre-adolescents" OR "pre-adolescence" OR "schoolage" OR "schoolboy" OR "schoolboys" OR "schoolgirl" OR "schoolgirls" OR "pre-puber" OR "pre-puberty" OR "prepuber" OR "prepubers" OR "prepuberty" OR "puber" OR "puberty" OR "puberal" OR "teenager" OR "teenagers" OR "teens" OR "youth" OR "youths" OR "underaged" OR "under-aged" OR exp "Pediatrics" OR "Pediatric" OR "Pediatrics" OR "Paediatric" OR "Paediatrics" **OR** "child" OR children* OR schoolchild* OR "infant" OR "infants" OR "infancy" OR adolesc* OR pediat* OR paediat* OR neonat* OR toddler* OR "teen" OR "teens" OR teenager* OR preteen* OR newborn* OR postneonat* OR postnatal* OR "puberty" OR preschool* OR suckling* OR "juvenile" OR "new born" OR "new borns" OR new-born* OR neo-nat* OR neonat* OR perinat* OR underag* OR "under age" OR "under aged" OR youth* OR kinder* OR pubescen* OR prepubescen* OR "prepuberty" OR "school age" OR "schoolage" OR "school ages" OR schoolage*) OR TI=("one year old" OR "two year old" OR "three year old" OR "four year old" OR "five year old" OR "six year old" OR "seven year old" OR "eight year old" OR "nine year old" OR "ten year old" OR "eleven year old" OR "twelve year old" OR "thirteen year old" OR "fourteen year old" OR "fifteen year old" OR "sixteen year old" OR "seventeen year old" OR "eighteen year old" OR "1 year old" OR "2 year old" OR "3 year old" OR "4 year old" OR "5 year old" OR "6 year old" OR "7 year old" OR "8 year old" OR "9 year old" OR "10 year old" OR "11 year old" OR "12 year old" OR "13 year old" OR "14 year old" OR "15 year old" OR "16 year old" OR "17 year old" OR "18 year old" OR "two years old" OR "three years old" OR "four years old" OR "five years old" OR "six years old" OR "seven years old" OR "eight years old" OR "nine years old" OR "ten years old" OR "eleven years old" OR "twelve years old" OR "thirteen years old" OR "fourteen years old" OR "fifteen years old" OR "sixteen years old" OR "seventeen years old" OR "eighteen years old" OR "2 years old" OR "3 years old" OR "4 years old" OR "5 years old" OR "6 years old" OR "7 years old" OR "8 years old" OR "9 years old" OR "10 years old" OR "11 years old" OR "12 years old" OR "13 years old" OR "14 years old" OR "15 years old" OR "16 years old" OR "17 years old" OR "18 years old")) AND (TI=("orthopedic trauma" OR "orthopedic traumas" OR "orthopaedic trauma" OR "orthopaedic traumas" OR "orthopedic injury" OR "orthopedic injuries" OR "orthopaedic injury" OR "orthopaedic injuries" OR "orthopedic surgery" OR "orthopedic surgeries" OR "orthopaedic surgery" OR "orthopaedic surgeries" OR "orthopedic intervention" OR "orthopedic interventions" OR "orthopaedic intervention" OR "orthopaedic interventions" OR "orthopedic trauma" OR "orthopedic procedures" OR "orthopaedic procedure" OR "orthopaedic procedures" OR "Arm Injury" OR "Back Injury" OR "Fracture" OR "Hand Injury" OR "Hip Injury" OR "Joint Dislocations" OR "Leg Injury" OR "Neck Injury" OR "Shoulder Injury" OR "Sprain" OR "Tendon Injury" OR "Ankle Fractures" OR "Ankle Injuries" OR "Arm Injuries" OR "Back Injuries" OR "Bone Diastasis" OR "Elbow Fractures" OR "Elbow Injuries" OR "Femoral Fractures" OR "Fibula Fractures" OR "Finger Injuries" OR "Foot Injuries" OR "Forearm Injuries" OR "Fracture" OR "Fracture Dislocation" OR "Fractures" OR "Hand Injuries" OR "Hip Dislocation" OR "Hip Fractures" OR "Hip Injuries" OR "Humeral Fractures" OR "Intra-Articular Fractures" OR "Joint Dislocations" OR "Knee Dislocation" OR "Knee Fractures" OR "Knee Injuries" OR "Leg Injuries" OR "Medial Tibial Stress Syndrome" OR "Muscle Diastasis" OR "Neck Injuries" OR "Osteoporotic Fractures" OR "Patellar Dislocation" OR "Periprosthetic Fractures" OR "Radius Fractures" OR "Rib Fractures" OR "Rotator Cuff Injuries" OR "Shoulder Dislocation" OR "Shoulder Fractures" OR "Shoulder Impingement Syndrome" OR "Shoulder Injuries" OR "Skull Fractures" OR "Spinal Fractures" OR "Spinal Injuries" OR "Sprain" OR "Tendinopathy" OR "Tendon Injuries" OR "Tibial Fractures" OR "Tibial Meniscus Injuries" OR "Traumatic Amputation" OR "Traumatic Multiple Amputations" OR "Ulna Fractures" OR "Whiplash Injuries" OR "Wrist Fractures" OR "Wrist Injuries" OR "Ankle Fracture" OR "Ankle Injury" OR "Arm Injury" OR "Back Injury" OR "Elbow Fracture" OR "Elbow Injury" OR "Femoral Fracture" OR "Fibula Fracture" OR "Finger Injury" OR "Foot Injury" OR "Forearm Injury" OR "Fracture Dislocations" OR "Hand Injury" OR "Hip Dislocations" OR "Hip Fracture" OR "Hip Injury" OR "Humeral Fracture" OR "Humeral Fracture" OR "Intra-Articular Fracture" OR "Joint Dislocation" OR "Knee Dislocations" OR "Knee Fracture" OR "Knee Injury" OR "Leg Injury" OR "Neck Injury" OR "Osteoporotic Fracture" OR "Patellar Dislocations" OR "Periprosthetic Fracture" OR "Radius Fracture" OR "Rib Fracture" OR "Rotator Cuff Injury" OR "Shoulder Dislocations" OR "Shoulder Fracture" OR "Shoulder Impingement" OR "Shoulder Injury" OR "Skull Fracture" OR "Spinal Fracture" OR "Spinal Injury" OR "Sprains" OR "Tendinopathies" OR "Tendon Injury" OR "Tibial Fracture" OR "Tibial Meniscus Injury" OR "Traumatic Amputations" OR "Traumatic Multiple Amputation" OR "Ulna Fracture" OR "Whiplash Injury" OR "Wrist Fracture" OR "Wrist Injury" OR "Orthopedics" OR "Orthopedic Surgery" OR "Acetabuloplast*" OR "Acetabuloplasty" OR "Alveolar Bone Graft*" OR "Alveolar Bone Grafting" OR "Ankle Replacement" OR "Anterior Cruciate Ligament Reconstruction" OR "Arthrodesis" OR "Arthroplast*" OR "Arthroplasty" OR "Arthroscop*" OR "Arthroscopy" OR "Bone Lengthening" OR "Bone Transplant*" OR "Bone Transplantation" OR "Bone-Patellar Tendon-Bone Graft*" OR "Bone-Patellar Tendon-Bone Grafting" OR "Cementoplast*" OR "Cementoplasty" OR "Disarticulation" OR "Diskectom*" OR "Diskectomy" OR "Distraction Osteogenesis" OR "Elbow Replacement" OR "Finger Replacement" OR "Fracture Fixation" OR "Genioplast*" OR "Genioplasty" OR "Hemiarthroplast*" OR "Hemiarthroplasty" OR "Hemipelvectom*" OR "Hemipelvectomy" OR "Hip Replacement" OR "Ilizarov Technique" OR "Joint Capsule Release" OR "Knee Replacement" OR "Kyphoplast*" OR "Kyphoplasty" OR "Laminectom*" OR "Laminectomy" OR "Le Fort Osteotom*" OR "Le Fort Osteotomy" OR "Limb Salvage" OR "Mandibular Osteotom*" OR "Mandibular Osteotomy" OR "Mandibular Reconstruction" OR "Maxillary Osteotom*" OR "Maxillary Osteotomy" OR "Meniscectom*" OR "Meniscectomy" OR "Open Fracture Reduction" OR "Orthognathic Surgical Procedure" OR "Orthognathic Surgical Procedures" OR "Osteotom*" OR "Osteotomy" OR "Posterior Cruciate Ligament Reconstruction" OR "Replacement Arthroplast*" OR "Replacement Arthroplasty" OR "Sagittal Split Ramus Osteotom*" OR "Sagittal Split Ramus Osteotomy" OR "Shoulder Replacement" OR "Sinus Floor Augmentation" OR "Spinal Fusion" OR "Surgical Amputation" OR "Synovectom*" OR "Synovectomy" OR "Tendon Transfer" OR "Tenodesis" OR "Tenotom*" OR "Tenotomy" OR "Total Disc Replacement" OR "Traction" OR "Ulnar Collateral Ligament Reconstruction" OR "Vertebroplast*" OR "Vertebroplasty" OR "Orthopedic Equipment" OR "orthopedic" OR "orthopedics" OR "orthopedic*" OR "orthopaedic" OR "orthopaedics" OR "orthopaedic*") OR AK=("orthopedic trauma" OR "orthopedic traumas" OR "orthopaedic trauma" OR "orthopaedic traumas" OR "orthopedic injury" OR "orthopedic injuries" OR "orthopaedic injury" OR "orthopaedic injuries" OR "orthopedic surgery" OR "orthopedic surgeries" OR "orthopaedic surgery" OR "orthopaedic surgeries" OR "orthopedic intervention" OR "orthopedic interventions" OR "orthopaedic intervention" OR "orthopaedic interventions" OR "orthopedic trauma" OR "orthopedic procedures" OR "orthopaedic procedure" OR "orthopaedic procedures" OR "Arm Injury" OR "Back Injury" OR "Fracture" OR "Hand Injury" OR "Hip Injury" OR "Joint Dislocations" OR "Leg Injury" OR "Neck Injury" OR "Shoulder Injury" OR "Sprain" OR "Tendon Injury" OR "Ankle Fractures" OR "Ankle Injuries" OR "Arm Injuries" OR "Back Injuries" OR "Bone Diastasis" OR "Elbow Fractures" OR "Elbow Injuries" OR "Femoral Fractures" OR "Fibula Fractures" OR "Finger Injuries" OR "Foot Injuries" OR "Forearm Injuries" OR "Fracture" OR "Fracture Dislocation" OR "Fractures" OR "Hand Injuries" OR "Hip Dislocation" OR "Hip Fractures" OR "Hip Injuries" OR "Humeral Fractures" OR "Intra-Articular Fractures" OR "Joint Dislocations" OR "Knee Dislocation" OR "Knee Fractures" OR "Knee Injuries" OR "Leg Injuries" OR "Medial Tibial Stress Syndrome" OR "Muscle Diastasis" OR "Neck Injuries" OR "Osteoporotic Fractures" OR "Patellar Dislocation" OR "Periprosthetic Fractures" OR "Radius Fractures" OR "Rib Fractures" OR "Rotator Cuff Injuries" OR "Shoulder Dislocation" OR "Shoulder Fractures" OR "Shoulder Impingement Syndrome" OR "Shoulder Injuries" OR "Skull Fractures" OR "Spinal Fractures" OR "Spinal Injuries" OR "Sprain" OR "Tendinopathy" OR "Tendon Injuries" OR "Tibial Fractures" OR "Tibial Meniscus Injuries" OR "Traumatic Amputation" OR "Traumatic Multiple Amputations" OR "Ulna Fractures" OR "Whiplash Injuries" OR "Wrist Fractures" OR "Wrist Injuries" OR "Ankle Fracture" OR "Ankle Injury" OR "Arm Injury" OR "Back Injury" OR "Elbow Fracture" OR "Elbow Injury" OR "Femoral Fracture" OR "Fibula Fracture" OR "Finger Injury" OR "Foot Injury" OR "Forearm Injury" OR "Fracture Dislocations" OR "Hand Injury" OR "Hip Dislocations" OR "Hip Fracture" OR "Hip Injury" OR "Humeral Fracture" OR "Humeral Fracture" OR "Intra-Articular Fracture" OR "Joint Dislocation" OR "Knee Dislocations" OR "Knee Fracture" OR "Knee Injury" OR "Leg Injury" OR "Neck Injury" OR "Osteoporotic Fracture" OR "Patellar Dislocations" OR "Periprosthetic Fracture" OR "Radius Fracture" OR "Rib Fracture" OR "Rotator Cuff Injury" OR "Shoulder Dislocations" OR "Shoulder Fracture" OR "Shoulder Impingement" OR "Shoulder Injury" OR "Skull Fracture" OR "Spinal Fracture" OR "Spinal Injury" OR "Sprains" OR "Tendinopathies" OR "Tendon Injury" OR "Tibial Fracture" OR "Tibial Meniscus Injury" OR "Traumatic Amputations" OR "Traumatic Multiple Amputation" OR "Ulna Fracture" OR "Whiplash Injury" OR "Wrist Fracture" OR "Wrist Injury" OR "Orthopedics" OR "Orthopedic Surgery" OR "Acetabuloplast*" OR "Acetabuloplasty" OR "Alveolar Bone Graft*" OR "Alveolar Bone Grafting" OR "Ankle Replacement" OR "Anterior Cruciate Ligament Reconstruction" OR "Arthrodesis" OR "Arthroplast*" OR "Arthroplasty" OR "Arthroscop*" OR "Arthroscopy" OR "Bone Lengthening" OR "Bone Transplant*" OR "Bone Transplantation" OR "Bone-Patellar Tendon-Bone Graft*" OR "Bone-Patellar Tendon-Bone Grafting" OR "Cementoplast*" OR "Cementoplasty" OR "Disarticulation" OR "Diskectom*" OR "Diskectomy" OR "Distraction Osteogenesis" OR "Elbow Replacement" OR "Finger Replacement" OR "Fracture Fixation" OR "Genioplast*" OR "Genioplasty" OR "Hemiarthroplast*" OR "Hemiarthroplasty" OR "Hemipelvectom*" OR "Hemipelvectomy" OR "Hip Replacement" OR "Ilizarov Technique" OR "Joint Capsule Release" OR "Knee Replacement" OR "Kyphoplast*" OR "Kyphoplasty" OR "Laminectom*" OR "Laminectomy" OR "Le Fort Osteotom*" OR "Le Fort Osteotomy" OR "Limb Salvage" OR "Mandibular Osteotom*" OR "Mandibular Osteotomy" OR "Mandibular Reconstruction" OR "Maxillary Osteotom*" OR "Maxillary Osteotomy" OR "Meniscectom*" OR "Meniscectomy" OR "Open Fracture Reduction" OR "Orthognathic Surgical Procedure" OR "Orthognathic Surgical Procedures" OR "Osteotom*" OR "Osteotomy" OR "Posterior Cruciate Ligament Reconstruction" OR "Replacement Arthroplast*" OR "Replacement Arthroplasty" OR "Sagittal Split Ramus Osteotom*" OR "Sagittal Split Ramus Osteotomy" OR "Shoulder Replacement" OR "Sinus Floor Augmentation" OR "Spinal Fusion" OR "Surgical Amputation" OR "Synovectom*" OR "Synovectomy" OR "Tendon Transfer" OR "Tenodesis" OR "Tenotom*" OR "Tenotomy" OR "Total Disc Replacement" OR "Traction" OR "Ulnar Collateral Ligament Reconstruction" OR "Vertebroplast*" OR "Vertebroplasty" OR "Orthopedic Equipment" OR "orthopedic" OR "orthopedics" OR "orthopedic*" OR "orthopaedic" OR "orthopaedics" OR "orthopaedic*") OR AB=("orthopedic trauma" OR "orthopedic traumas" OR "orthopaedic trauma" OR "orthopaedic traumas" OR "orthopedic injury" OR "orthopedic injuries" OR "orthopaedic injury" OR "orthopaedic injuries" OR "orthopedic surgery" OR "orthopedic surgeries" OR "orthopaedic surgery" OR "orthopaedic surgeries" OR "orthopedic intervention" OR "orthopedic interventions" OR "orthopaedic intervention" OR "orthopaedic interventions" OR "orthopedic trauma" OR "orthopedic procedures" OR "orthopaedic procedure" OR "orthopaedic procedures" OR "Arm Injury" OR "Back Injury" OR "Fracture" OR "Hand Injury" OR "Hip Injury" OR "Joint Dislocations" OR "Leg Injury" OR "Neck Injury" OR "Shoulder Injury" OR "Sprain" OR "Tendon Injury" OR "Ankle Fractures" OR "Ankle Injuries" OR "Arm Injuries" OR "Back Injuries" OR "Bone Diastasis" OR "Elbow Fractures" OR "Elbow Injuries" OR "Femoral Fractures" OR "Fibula Fractures" OR "Finger Injuries" OR "Foot Injuries" OR "Forearm Injuries" OR "Fracture" OR "Fracture Dislocation" OR "Fractures" OR "Hand Injuries" OR "Hip Dislocation" OR "Hip Fractures" OR "Hip Injuries" OR "Humeral Fractures" OR "Intra-Articular Fractures" OR "Joint Dislocations" OR "Knee Dislocation" OR "Knee Fractures" OR "Knee Injuries" OR "Leg Injuries" OR "Medial Tibial Stress Syndrome" OR "Muscle Diastasis" OR "Neck Injuries" OR "Osteoporotic Fractures" OR "Patellar Dislocation" OR "Periprosthetic Fractures" OR "Radius Fractures" OR "Rib Fractures" OR "Rotator Cuff Injuries" OR "Shoulder Dislocation" OR "Shoulder Fractures" OR "Shoulder Impingement Syndrome" OR "Shoulder Injuries" OR "Skull Fractures" OR "Spinal Fractures" OR "Spinal Injuries" OR "Sprain" OR "Tendinopathy" OR "Tendon Injuries" OR "Tibial Fractures" OR "Tibial Meniscus Injuries" OR "Traumatic Amputation" OR "Traumatic Multiple Amputations" OR "Ulna Fractures" OR "Whiplash Injuries" OR "Wrist Fractures" OR "Wrist Injuries" OR "Ankle Fracture" OR "Ankle Injury" OR "Arm Injury" OR "Back Injury" OR "Elbow Fracture" OR "Elbow Injury" OR "Femoral Fracture" OR "Fibula Fracture" OR "Finger Injury" OR "Foot Injury" OR "Forearm Injury" OR "Fracture Dislocations" OR "Hand Injury" OR "Hip Dislocations" OR "Hip Fracture" OR "Hip Injury" OR "Humeral Fracture" OR "Humeral Fracture" OR "Intra-Articular Fracture" OR "Joint Dislocation" OR "Knee Dislocations" OR "Knee Fracture" OR "Knee Injury" OR "Leg Injury" OR "Neck Injury" OR "Osteoporotic Fracture" OR "Patellar Dislocations" OR "Periprosthetic Fracture" OR "Radius Fracture" OR "Rib Fracture" OR "Rotator Cuff Injury" OR "Shoulder Dislocations" OR "Shoulder Fracture" OR "Shoulder Impingement" OR "Shoulder Injury" OR "Skull Fracture" OR "Spinal Fracture" OR "Spinal Injury" OR "Sprains" OR "Tendinopathies" OR "Tendon Injury" OR "Tibial Fracture" OR "Tibial Meniscus Injury" OR "Traumatic Amputations" OR "Traumatic Multiple Amputation" OR "Ulna Fracture" OR "Whiplash Injury" OR "Wrist Fracture" OR "Wrist Injury" OR "Orthopedics" OR "Orthopedic Surgery" OR "Acetabuloplast*" OR "Acetabuloplasty" OR "Alveolar Bone Graft*" OR "Alveolar Bone Grafting" OR "Ankle Replacement" OR "Anterior Cruciate Ligament Reconstruction" OR "Arthrodesis" OR "Arthroplast*" OR "Arthroplasty" OR "Arthroscop*" OR "Arthroscopy" OR "Bone Lengthening" OR "Bone Transplant*" OR "Bone Transplantation" OR "Bone-Patellar Tendon-Bone Graft*" OR "Bone-Patellar Tendon-Bone Grafting" OR "Cementoplast*" OR "Cementoplasty" OR "Disarticulation" OR "Diskectom*" OR "Diskectomy" OR "Distraction Osteogenesis" OR "Elbow Replacement" OR "Finger Replacement" OR "Fracture Fixation" OR "Genioplast*" OR "Genioplasty" OR "Hemiarthroplast*" OR "Hemiarthroplasty" OR "Hemipelvectom*" OR "Hemipelvectomy" OR "Hip Replacement" OR "Ilizarov Technique" OR "Joint Capsule Release" OR "Knee Replacement" OR "Kyphoplast*" OR "Kyphoplasty" OR "Laminectom*" OR "Laminectomy" OR "Le Fort Osteotom*" OR "Le Fort Osteotomy" OR "Limb Salvage" OR "Mandibular Osteotom*" OR "Mandibular Osteotomy" OR "Mandibular Reconstruction" OR "Maxillary Osteotom*" OR "Maxillary Osteotomy" OR "Meniscectom*" OR "Meniscectomy" OR "Open Fracture Reduction" OR "Orthognathic Surgical Procedure" OR "Orthognathic Surgical Procedures" OR "Osteotom*" OR "Osteotomy" OR "Posterior Cruciate Ligament Reconstruction" OR "Replacement Arthroplast*" OR "Replacement Arthroplasty" OR "Sagittal Split Ramus Osteotom*" OR "Sagittal Split Ramus Osteotomy" OR "Shoulder Replacement" OR "Sinus Floor Augmentation" OR "Spinal Fusion" OR "Surgical Amputation" OR "Synovectom*" OR "Synovectomy" OR "Tendon Transfer" OR "Tenodesis" OR "Tenotom*" OR "Tenotomy" OR "Total Disc Replacement" OR "Traction" OR "Ulnar Collateral Ligament Reconstruction" OR "Vertebroplast*" OR "Vertebroplasty" OR "Orthopedic Equipment" OR "orthopedic" OR "orthopedics" OR "orthopedic*" OR "orthopaedic" OR "orthopaedics" OR "orthopaedic*"))) NOT DT=("meeting abstract")
