# Supplementary material for: Prevalence of posttraumatic stress disorder in paediatric patients following orthopaedic trauma: A systematic review
Source: J Child Orthop. 2026 Feb 18;20(2):103–10. doi: 10.1177/18632521261419773 (PMC12920159; doi:10.1177/18632521261419773)
Supplement: sj-docx-2-cho-10.1177_18632521261419773 – Supplemental material for Prevalence of posttraumatic stress disorder in paediatric patients following orthopaedic trauma: A systematic review [file sj-docx-2-cho-10.1177_18632521261419773.docx]

**Supplementary materials**

**Supplementary Table 1: Quality assessment of included studies**

| Authors | 1 | 2 | 3 | 4 | 5a | 5b | 6a | 6b | 7 | 8 | 9 | 10 | 11 | 12 | Quality |
| --- | --- | --- | --- | --- | --- | --- | --- | --- | --- | --- | --- | --- | --- | --- | --- |
| Al Zomia (2023)^32^ | Yes | Yes | Yes | Yes | No | No | No | Can't tell | No | No | Can't tell | Yes | Yes | No | Fair |
| Hajek (2009)^45^ | Yes | Yes | Yes | Yes | No | No | Can't tell | Yes | Yes | No | Can't tell | Can't tell | Can't tell | Yes | Fair |
| Levi (1999)^17^ | Yes | No | Yes | Yes | No | No | No | Yes | Yes | Can't tell | Can't tell | Yes | Can't tell | Yes | Fair |
| May (2023)^33^ | Yes | Yes | Yes | Yes | No | No | Can't tell | Yes | No | Yes | Yes | Yes | Can't tell | Yes | Good |
| McKinnon (2017)^34^ | Yes | Yes | Yes | Yes | No | No | Yes | Yes | Yes | Yes | Can't tell | Yes | Yes | Yes | Good |
| Messner (2020)^18^ | Yes | Yes | Yes | Can't tell | No | No | Can't tell | Yes | No | No | Can't tell | Yes | Yes | Yes | Fair |
| Sanders (2005)^35^ | Yes | Yes | Yes | Yes | No | No | Can't tell | Yes | No | No | Can't tell | Yes | No | Yes | Fair |
| Subasi (2003)^46^ | Yes | Can't tell | Yes | Yes | No | No | Can't tell | Yes | No | No | Can't tell | Can't tell | Yes | Yes | Fair |
| Vitale (2006)^36^ | Yes | Yes | Yes | Yes | No | No | No | Yes | No | No | Can't tell | Yes | No | No | Fair |
| Wallace (2012)^37^ | No | Yes | Yes | Yes | No | No | Can't tell | Yes | No | No | Can't tell | Yes | Yes | No | Fair |

**Index:** 1) Did the study address a clearly focused issue? 2) Was the cohort recruited in an acceptable way? 3) Was the exposure accurately measured to minimize bias? 4) Was the outcome accurately measured to minimize bias? 5a) Have the authors identified all important confounding factors? 5b) Have they taken account of the confounding factors in the design and/or analysis? 6a) Was the follow up of the subjects complete enough? 6b) Was the follow up of subjects long enough? 7) What are the results of this study? 8) How precise are the results? 9) Do you believe the results? 10) Can the results be applied to the local population? 11) Do the results of this study fit with other available evidence? 12) What are the implications of this study for practice?

**Supplementary Table 2: Prevalence of PTSD across studies, including weighted pooled prevalence**

| Authors | Total patients | Prevalence | Lower CI | Upper CI | Weight | Adjusted weight |
| --- | --- | --- | --- | --- | --- | --- |
| Al Zomia (2023)^32^ | 100 | 5.0% | 1.6% | 11.3% | 16.33 | 14.89 |
| Hajek (2009)^45^ | 99 | 7.0% | 2.3% | 12.7% | 14.21 | 12.95 |
| Levi (1999)^17^ | 59 | 29.4% 1.9% | 15.0%  -1.5% | 38.4%  5.4% | 2.81 | 2.56 |
| May (2023)^33^ | 176 | 14.7% | 10.0% | 21.1% | 12.47 | 11.37 |
| McKinnon (2017)^34^ | 57 | 12.3% | 5.1% | 23.7% | 4.44 | 4.05 |
| Messner (2020)^18^ | 32 | 38.1% | 11.5% | 43.4% | 1.51 | 1.38 |
| Sanders (2005)^35^ | 400 | 33.0% | 28.4% | 37.8% | 17.39 | 15.85 |
| Subasi (2003)^46^ | 58 | 10.3% | 3.9% | 21.2% | 5.13 | 4.68 |
| Vitale (2006)^36^ | 299 | 9.5% | 6.3% | 13.2% | 32.28 | 29.42 |
| Wallace (2012)^37^ | 76 | 32.9% | 22.5% | 44.6% | 3.15 | 2.87 |
| POOLED | **1356** | **14.5%** | -5.1% | 34.1% |  |  |
